# Design, synthesis, biological assessment and molecular modeling studies of novel imidazothiazole-thiazolidinone hybrids as potential anticancer and anti-inflammatory agents

**DOI:** 10.1038/s41598-024-59063-x

**Published:** 2024-04-11

**Authors:** Payal Kamboj, Anjali, Khalid Imtiyaz, Moshahid A. Rizvi, Virendra Nath, Vipin Kumar, Asif Husain, Mohd. Amir

**Affiliations:** 1 Department of Pharmaceutical Chemistry, School of Pharmaceutical Education and Research, Jamia Hamdard, New Delhi, 110062 India; 2 Genome Biology Lab, Department of Biosciences, Jamia Millia Islamia, New Delhi, India; 3 Department of Pharmacy, Central University of Rajasthan, Ajmer, India

**Keywords:** Imidazothiazole, EGFR kinase, Anticancer, Anti-inflammatory, Molecular dynamics, Medicinal chemistry, Pharmacology

## Abstract

A new series of imidazothiazole derivatives bearing thiazolidinone moiety (**4a**-**g** and **5a**-**d**) were designed, synthesized and evaluated for potential epidermal growth factor receptor (EGFR) kinase inhibition, anticancer and anti-inflammatory activity, cardiomyopathy toxicity and hepatotoxicity. Compound **4c** inhibited EGFR kinase at a concentration of 18.35 ± 1.25 µM, whereas standard drug erlotinib showed IC_50_ value of 06.12 ± 0.92 µM. The molecular docking, dynamics simulation and MM-GBSA binding energy calculations revealed strong interaction of compound **4c** with binding site of EGFR. The synthesized compounds were evaluated for their anticancer activity by MTT assay against three human cancer cell lines A549 (Lung), MCF-7 (Breast), HCT116 (Colon), one normal human embryonic kidney cell line HEK293 and also for their EGFR kinase inhibitory activity. Few compounds of the series (**4a**, **4b**, **4c**) showed promising growth inhibition against all the tested cancer cell lines and against EGFR kinase. Among these, compound **4c** was found to be most active and displayed IC_50_ value of 10.74 ± 0.40, 18.73 ± 0.88 against cancer cell lines A549 and MCF7 respectively whereas it showed an IC_50_ value of 96.38 ± 1.79 against HEK293 cell line indicating lesser cytotoxicity for healthy cell. Compounds **4a**, **4b** and **4c** were also examined for their apoptosis inducing potential through AO/EB dual staining assay and it was observed that their antiproliferative activity against A549 cells is mediated via induction of apoptosis. Cardiomyopathy studies showed normal cardiomyocytes with no marked sign of pyknotic nucleus of compounds **4b** and **4c**. Hepatotoxicity studies of compounds **4b** and **4c** also showed normal architecture of hepatocytes. Compounds **4a**-**g** and **5a**-**d** were also evaluated for their *in-vitro* anti-inflammatory activity by protein albumin denaturation assay. Among the tested compounds **4a**-**d** and **5a**-**b** showed promising activity and were selected for *in-vivo* inflammatory activity against carrageenan rat paw edema test. Among these compounds, **4b** was found to be most active in the series showing 84.94% inhibition, whereas the standard drug diclofenac sodium showed 84.57% inhibition. Compound **4b** also showed low ulcerogenic potential and lipid peroxidation. Thus, compounds **4c** and **4b** could be a promising lead compounds for developing anticancer and anti-inflammatory agents with low toxicity and selectivity.

## Introduction

Cancer is characterized by uncontrolled proliferation of cells, often resulting in invasion and metastasis to both nearby and distant tissues and organs. According to various surveys or statistical data, cancer is second among the leading causes of mortality and accounts for millions of deaths every year preceded only by heart diseases^1^. A surge in cancer incidence has a negative impact on the global healthcare system; in contrast, the majority of anticancer medications have limited therapeutic potency due to lack of specificity and significant adverse effects. Therefore, new therapeutic anticancer drugs with the highest efficacy and lowest toxicity are the need of hour to address such issues^2–4^.

Epidermal growth factor receptor (EGFR) is a receptor that is overexpressed or mutated in various types of cancer, making it an attractive target for therapeutic interventions^5^. The abnormal activation of EGFR in cancer cells can lead to uncontrolled cell growth, survival, and metastasis^6^. Therefore, inhibiting EGFR signalling is a strategy aimed at reducing cancer cell proliferation and promoting tumor regression^7^. EGFR is a glycoprotein and a member of the ErbB family of tyrosine kinase receptors, which consist of four member ErbB-1, ErbB-2, ErbB-3 and ErbB-4^8^. When ligand binds to the EGFR, it activates various pathways such as PI3K, JAK-STAT, and RAS that helps in controlling of proliferation, transcription, and cell death^9^. Different cancer types may have distinct EGFR mutations or alterations, which can affect their response to EGFR inhibitors^10^. Thus, EGFR has become a very important target and has been clinically approved in the field of cancer therapy^11^.

Additionally, in a study it was observed that inhibiting EGFR led to a reduction in inflammation. Recent research has highlighted the importance of the EGFR-ERK pathway in various inflammatory conditions^12–17^. One study demonstrated that tris(2-chloroethyl) phosphate (TCEP) induces inflammation in HepG2 cancer cells by activating EGFR, which can be counteracted by inhibiting EGFR^18^. Furthermore, it has been established that the activation of EGFR plays a crucial role in the initiation and progression of respiratory inflammation induced by respiratory syncytial virus (RSV) and non-typeable Haemophilus influenzae (NTHi)^19,20^. Another study reported a crucial regulatory role for EGFR in thrombin-mediated inflammation^21^. Furthermore, a study focusing on obesity-related cardiovascular diseases found that inhibiting EGFR reduced inflammation induced by palmitic acid in cardiac muscle cells^22^.

**Figure 1:**
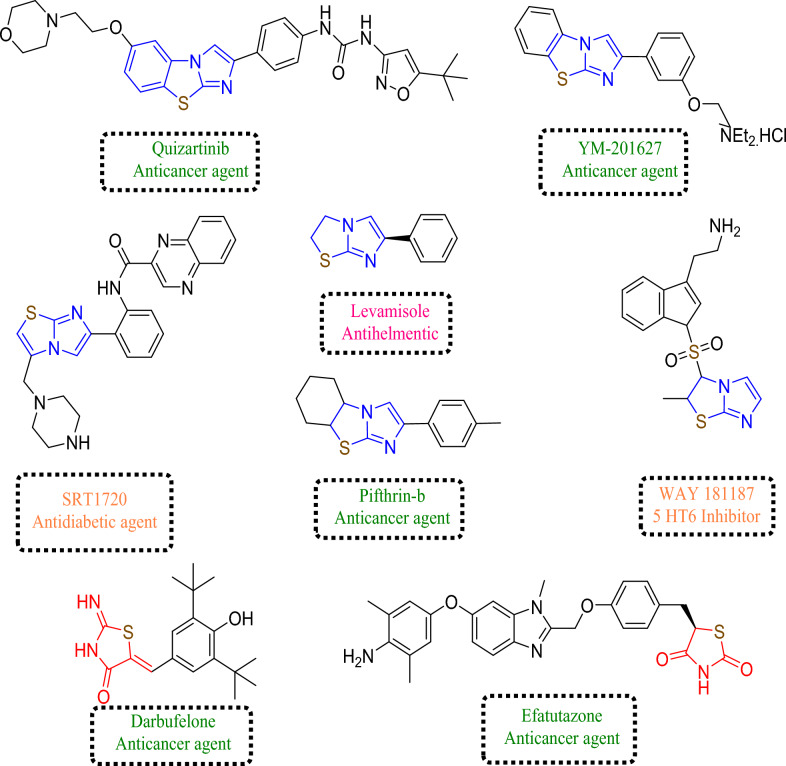
Imidazothiazole and thiazolidinone ring based biologically active marketed and drugs in clinical trial.

**Figure 2:**
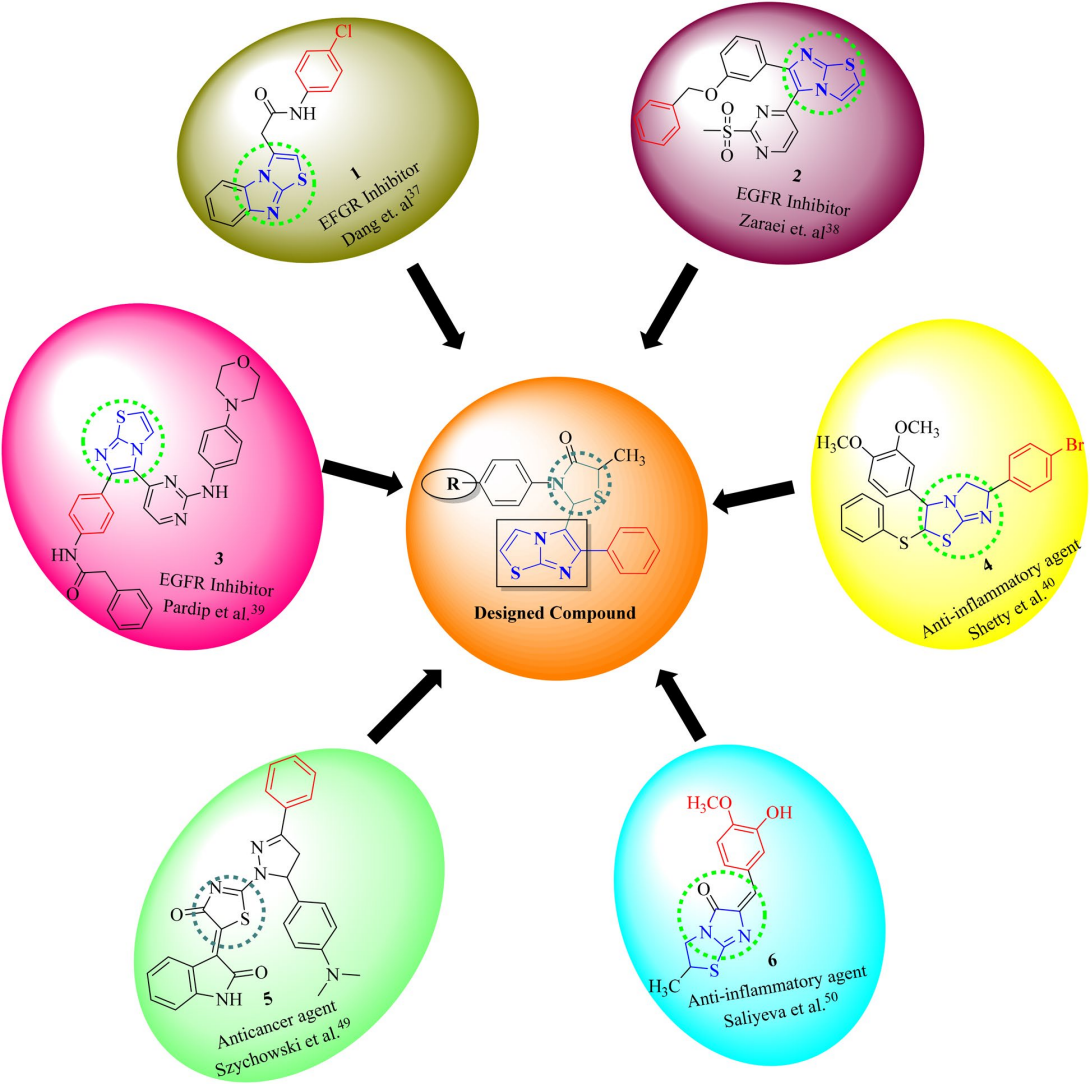
Imidazothiazole and thiazolidinone based anticancer and anti-inflammatory agents.

Imidazothiazole is considered a key scaffold for developing numerous pharmacologically active molecules among broad fused heterocyclic framework^23,24^ and their structural arrangement facilitates the facile binding to receptors and enzymes, resulting in synergistic biological activities such as anticancer^25^, anti-inflammatory^26^, antiviral^27^ antitubercular^28^, and antimicrobial^29^. Various classes of potent biologically active imidazothiazole drugs are available in the market and are in various phases of clinical development such as levamisole, Pifthrin- β, Quizartinib, SRT1720, YM-201627, and WAY-181187 (Fig. 1)^30–36^. Therefore, imidazothiazole have attracted a lot of researchers due to their potent anticancer activity. Deng et al*.*^37^ have reported imidazothiazole derivatives as potent EGFR inhibitors. Compound **1** of the series was found to be potent EGFR inhibitor with an IC_50_ value of 14.05 µmol/ml, it also displayed potent anticancer activity against HeLa cancer cell line with an IC_50_ value of 0.42 µM. Zaraei et al*.*^38^ have reported compound **2**, an imidazothiazole derivative as potent and selective ErbB4 kinase inhibitor displaying an IC_50_ value of 15.24 nM. It also has potent antiproliferative activity with IC_50_ values of 1.02,1.04, and 1.67 µM against MOLT-4 leukaemia, MDA-MB-231 breast and DU-145 prostate cancer cell lines. Pradip et al.^39^ have reported imidazothizole derivative (**3**) as potent EGFR and IGF1R inhibitor. It displayed strong inhibitory activity against EGFR (IC_50_ 35.5 nM) and IGF1R (IC_50_ 52 nM). Compound **4** having an imidazothiazole moiety is also reported as potent anti-inflammatory agent by Shetty et al.^40^ It displayed potent in vivo anti-inflammatory against carrageenan-induced paw edema with 65% inhibition in comparison to standard drug phenylbutazone (Fig. 2).

4-Thiazolidinone moiety also played an important role in drug design and is a core structure in various synthetic pharmaceuticals displaying broad spectrum of a biological activities such as anticancer^41,42^, anti-inflammatory and analgesic^43^, antidiabetic^44^, and antitubercular^45^ Furthermore, there are some pharmaceuticals in the market having thiazolidinone and thiazolidinedione rings attached with other heterocyclic moieties such as darbufelone^46,47^ and efatutazone^48^ (Fig. 1) which exhibited anticancer activity. Furthermore Szychowski et al.^49^ have reported 4-thiazolidinone derivatives as potent anticancer agent. Compound **5** of the series has displayed excellent inhibitory activity (67.34%) against human squamous cell lines (SCC-15) at concertation 100 mM. It also displayed potent ROS generation inhibition in a dose dependent manner. Compound **6** having thiazolidinone moiety was reported by Saliyeva et al.^50^ as potent anti-inflammatory agent. It displayed significant activity with 40.3% inhibition when evaluated against carrageenan-induced paw edema inflammation in albino rats compared to standard drug diclofenac sodium which showed an inhibitory activity of 38.8% (Fig. 2).

On the basis of above observations, it was thought worthwhile to design and synthesize new imidazothiazole analogues possessing a thiazolidinone moiety (Fig. 2) in order to assess their potential EGFR kinase inhibitory, anticancer and anti-inflammatory properties. The newly synthesized compounds were evaluated for EGFR kinase inhibition studies using erlotinib as positive control. These compounds were also subjected to antiproliferative activity assays using three different human cancer cell lines and one normal human cell line. The most potent derivative from this series was further investigated through wound healing assay, nuclear staining, cell apoptosis analysis, and *in-vivo* safety evaluation. Compounds were also evaluated for their *in-vitro* anti-inflammatory activity by protein albumin denaturation assay and few active compounds were selected for their *in-vivo* inflammatory activity against carrageenan rat paw edema test. Furthermore, molecular docking studies were conducted to determine the possible binding pattern of the most active compound, while molecular dynamics simulations were performed to elucidate its stability at the EGFR kinase binding site.

## Result and discussion

### Chemistry

**Scheme 1:**
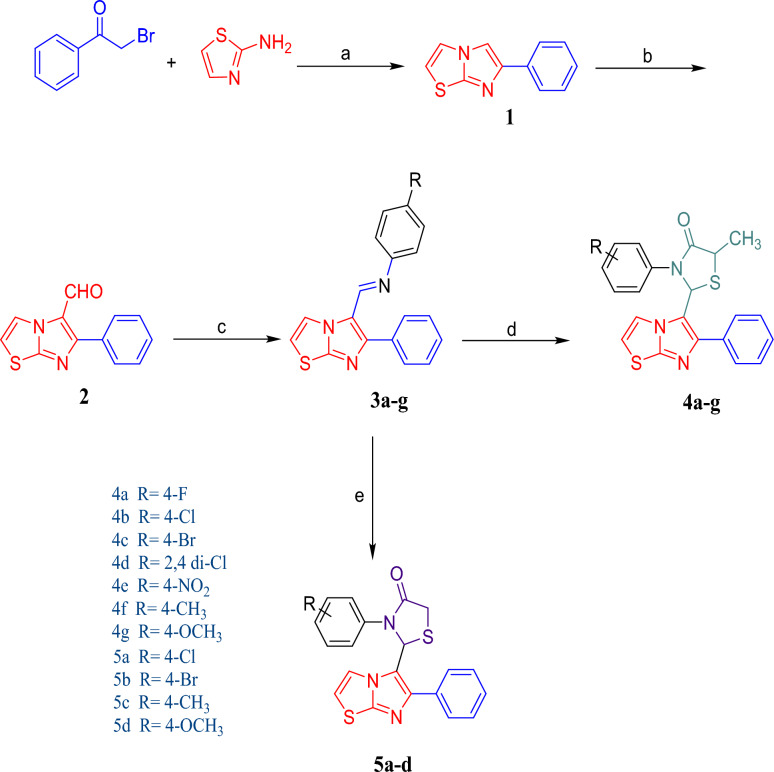
Reagent and Conditions. (**a**) Methanol, 70 °C, Reflux (**b**) CHCl_3,_ DMF, POCl_3_, 120 °C, Reflux (**c**) Substituted anilines, Toluene, PTSA, 120 °C (**d**) Thiolactic acid, 1–4 dioxane (**e**) Thioglycolic acid, 1–4 dioxane.

The synthetic strategy for compounds **4a-g** and **5a-d** were outlined in Scheme 1. Imidazothiazole **(1)** was prepared by reacting phenacyl bromide and aminothiazole in the presence of methanol under reflux condition for 24 h. Imidazothiazole carbaldehyde (**2**) was prepared by Vilsmeier-Haack reaction. 6-Phenylimidazo [2,1-b] thiazole-5-carbaldehyde (**2**) was treated with various substituted anilines for 5–8 h in the presence of toluene and *p*-toluene sulfonic acid as a catalyst to give Schiff bases **(3a-g)**. The target compounds **4a-g** and **5a-d** were synthesized by the reaction of intermediates **3a-g** with thiolactic acid and thioglycolic acid by stirring at room temperature for 16–20 h in the presence of catalytic amount of anhydrous zinc chloride and 1–4 dioxane. Structures of all the synthesized compounds were established on the basis of IR, ^1^H NMR, ^13^C NMR, and mass spectral data.

In the series **4a-g**, formation of thiazolidinone ring was confirmed by appearance of doublet for CH_3_ proton at δ 1.12 whereas quartet of CH proton was observed at δ 3.22. The CH proton of thiazolidinone ring linked to imidazothiazole moiety appeared at δ 6.05 confirming the formation of the compound **4c**. ^13^C NMR spectra (**4c**) showed signals of CH_3_ and CH carbons at δ 20.02 and δ 42.9 respectively. CH carbon of thiazolidinone ring linked to imidazothiazole moiety was observed at δ 66.82. The carbonyl group signal of thiazolidinone moiety appeared at δ 173.43.

Mass spectra of **4c** showed molecular ion peaks at (m/z) 469.9915 [M + H^+^] detected by HRMS (ESI) confirming the formation of compound. In the series **5a-d**, CH_2_ protons of thiazolidinone ring are splitting each other and their signals appeared as doublet at δ 3.48 and δ 3.52. The CH proton of thiazolidinone ring linked to imidazothiazole moiety appeared at δ 5.03 confirming the formation of the compound **5a**. ^13^C NMR spectra (**5a)** showed signal of CH_2_ carbon at δ 33.04, CH carbon of thiazolidinone ring linked to imidazothiazole moiety was observed at δ 56.50. The carbonyl group signal of thiazolidinone moiety appeared at δ 174.38. Mass spectra of **5a** showed molecular ion peaks at (m/z) 412.0283 [M + H^+^] detected by HRMS (ESI) confirming the formation of compound.

## Biological activity

### In vitro EGFR inhibitory activity

**Figure 3:**
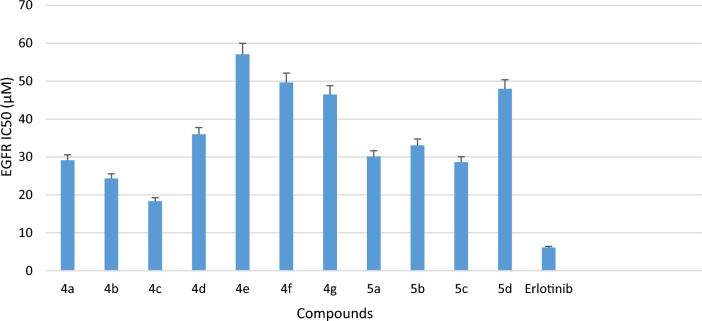
*In-vitro* cytotoxicity demonstrated by compounds **4a-g**, **5a-d** against EGFR kinase and compared with standard drug Erlotinib.

All the newly synthesized compounds **4a-g** and **5a-d** were studied for *in-vitro* EGFR kinase inhibitory activity using solid phase enzyme linked immunosorbent assay (ELISA) method and erlotinib was taken as standard drug. The inhibitory activities (IC_50_) of the compounds are summarized in Table 1, Fig. 3. Among all the tested compounds **4c**, **4b** and **4a** demonstrated potent inhibitory activity with IC_50_ value of 18.35 µM, 24.34 µM and 28.65 µM respectively with significant binding activity to the EGFR kinase. Other compounds of the series exhibited moderate activity with IC_50_ values in the range of 29.13 to 75.41 µM.

**Table 1:** *In-vitro* cytotoxicity against selected cancer lines, normal cell line and EGFR kinase inhibitory activity of synthesized compounds **4a-g** and **5a-d**.

Compd	R	IC_50_ (µM) (Mean ± SD)*				
		EGFR	A549	MCF7	HCT116	HEK293
**4a**	4-F	28.65 ± 1.96	22.52 ± 2.04	22.76 ± 5.50	23.69 ± 0.85	147.39 ± 4.30
**4b**	4-Cl	24.34 ± 1.73	21.06 ± 1.04	29.25 ± 7.10	45.04 ± 1.02	101.03 ± 3.41
**4c**	4-Br	18.35 ± 1.25	10.74 ± 0.40	18.73 ± 0.88	23.22 ± 1.89	96.38 ± 1.79
**4d**	2,4di-Cl	35.97 ± 1.89	31.84 ± 1.10	35.81 ± 1.03	49.50 ± 7.14	180.60 ± 5.74
**4e**	4-NO_2_	57.10 ± 0.38	50.56 ± 0.70	43.56 ± 7.19	89.92 ± 4.89	125.01 ± 5.80
**4f**	4-CH_3_	49.65 ± 4.89	40.22 ± 0.38	33.73 ± 0.84	47.58 ± 8.97	146.63 ± 4.57
**4g**	4-OCH_3_	46.47 ± 7.14	70.12 ± 2.02	52.85 ± 1.65	76.33 ± 5.66	163.73 ± 4.69
**5a**	4- Cl	30.13 ± 4.17	42.72 ± 3.35	35.06 ± 1.25	58.82 ± 3.14	117.40 ± 5.69
**5b**	4-Br	33.07 ± 1.58	35.51 ± 2.15	44.21 ± 0.85	58.61 ± 4.51	104.59 ± 3.50
**5c**	4- CH_3_	29.30 ± 4.61	34.24 ± 2.82	35.79 ± 4.61	59.95 ± 6.44	109.09 ± 5.32
**5d**	4-OCH_3_	47.98 ± 1.97	33.08 10.72	38.06 ± 0.89	65.18 ± 0.90	111.83 ± 0.25
**Erlotinib-**	–	06.12 ± 0.92	08.34 ± 8.09	10.66 ± 5.65	12.54 ± 1.87	–
**Erlotinib-**	–	–	–	–	–	80.15 ± 1.30

*All the experiments were performed in triplicate and the results were expressed as mean standard deviation (Mean ± SD); EGFR: Epidermal growth factor receptor; A549: lung cancer cell line. MCF7: Human breast cancer cell line, HCT116: Human colorectal cancer cell line, HEK293: Human embryonic kidney cell line, Erlotinib is used as standard drug.

## In vitro cytotoxicity

**Figure 4:**
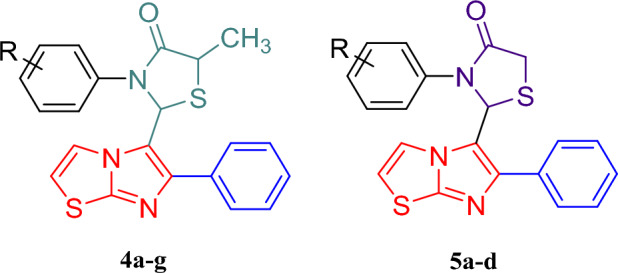
Imidazothiazole-thiazolidinone hybrids.

**Figure 5:**
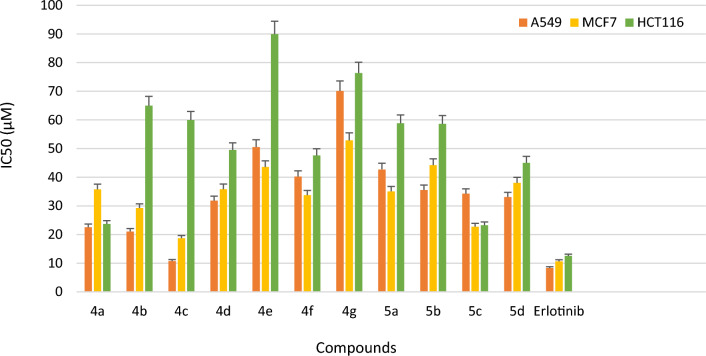
*In-vitro* anticancer activity of compounds **4a-g** and **5a-d** against A549, MCF7 and HCT116 cancer cell lines in comparison with standard drug Erlotinib.

**Figure 6:**
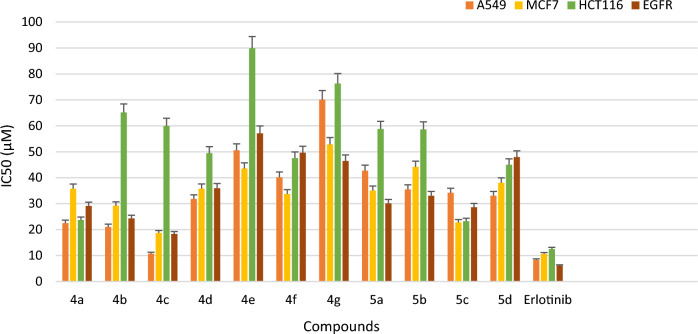
*In-vitro* cytotoxicity against selected cancer cell line A549, MCF7 and HCT116 and EGFR inhibitory activity demonstrated by synthesized derivatives **4a-g** and **5a–d** in comparison with standard drug Erlotinib.

All the synthesized imidazothiazole derivatives linked with thiazolidinone moiety (**4a-g** and **5a-d,** Fig. 4) were evaluated for their *in- vitro* anticancer activities against three selected human cancer cell lines namely non-small lung (A549), breast (MCF7) and colorectal (HCT116) and one normal human embryonic kidney cell line (HEK293) using the 3‐(4,5‐Dimethylthiazol‐2‐yl)-2,5‐Diphenyltetrazolium Bromide (MTT) assay method. All the synthesized compounds (**4a-g** and **5a-d**) exhibited excellent to moderate inhibitory activity compared to standard drug erlotinib with an IC_50_ values in range of 10.74 -76.33 µM against A549, MCF7 and HCT116 cancer cell lines respectively (Table 1, Fig. 5). From the results obtained, it was observed that compound **4c** has showed significant potency with an IC_50_ value of 10.74 µM followed by compounds **4b** and **4a** which showed an IC_50_ value of 21.06 and 22.52 µM respectively compared to standard drug erlotinib (8.34 µM) against A549 cell line. Compound **4c** and **4a** was found active against MCF7 cell line and exhibited good inhibitory activity with an IC_50_ value of 18.73 and 22.76 µM respectively compare to standard drug erlotinib (IC_50_ 10.66 µM). Other derivatives like compounds **4b**, **4f**, **5a**, **5c and 4d** showed moderate activity against MCF-7 cell line with IC_50_ values of 29.25, 33.73, 35.06, 35.79 and 35.81 µM respectively. Activity against the HCT116 cell line was found to be moderate. Compounds **4c** and **4a** were found to be most active in the series exhibiting an IC_50_ value of 23.22 and 23.69 µM respectively compared to standard drug erlotinib (12.54 µM). Rest of the compounds showed moderate activity with an IC_50_ value in the range of 45.04 to 89.90 µM against HCT116 cell line. Thus, it was observed that in all the three tested human cancer cell lines, compounds **4a**, **4b** and **4c** were found to be most potent derivatives against the A549 cell line. It was also noted that compounds **4a**, **4b** and **4c** were also potent against EGFR kinase inhibition (Fig. 6).

**Figure 7:**
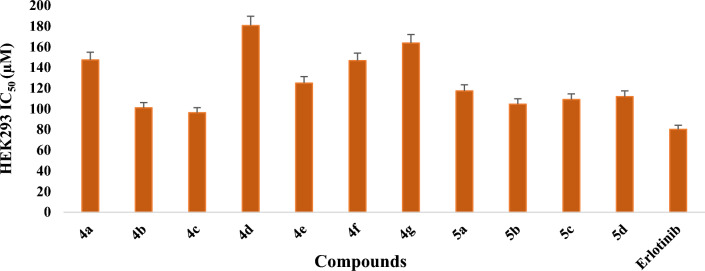
*In-vitro* cytotoxicity demonstrated by compounds **4a-g**, **5a-d** against normal cell line HEK-293 and compared with standard drug Erlotinib.

Furthermore, the evaluation against normal human embryonic kidney cell line (HEK293), demonstrated that the compounds were less toxic as compared with the standard drug erlotinib (Fig. 7). It has been observed that most of the time, IC_50_ values for a cancerous cell line are less, indicating that a lower concentration of drug is needed to kill the cancerous cells, whereas larger values of IC_50_ on a healthy cell line indicate that a higher dose of drug is needed for the death of healthy cells. The lead compound **4c** had IC_50_ value of 10.74 ± 0.40, 18.73 ± 0.88 and 23.22 ± 1.89 µM against A549, MCF7 and HCT116 cancer cell lines respectively, which suggests that **4c** is powerful enough to kill cancerous cells at lower concentrations. On the other hand, compound **4c** was found to be less toxic than the standard drug erlotinib (IC_50_ 80.15 ± 1.30 µM) for HEK293 cell line. For normal cell line, the value of IC_50_ for compound **4c** was found to be 96.38 ± 1.79 µM which indicates lesser cytotoxicity for healthy cell.

### Anti-migratory effect on A549 lung cancer cells

**Figure 8:**
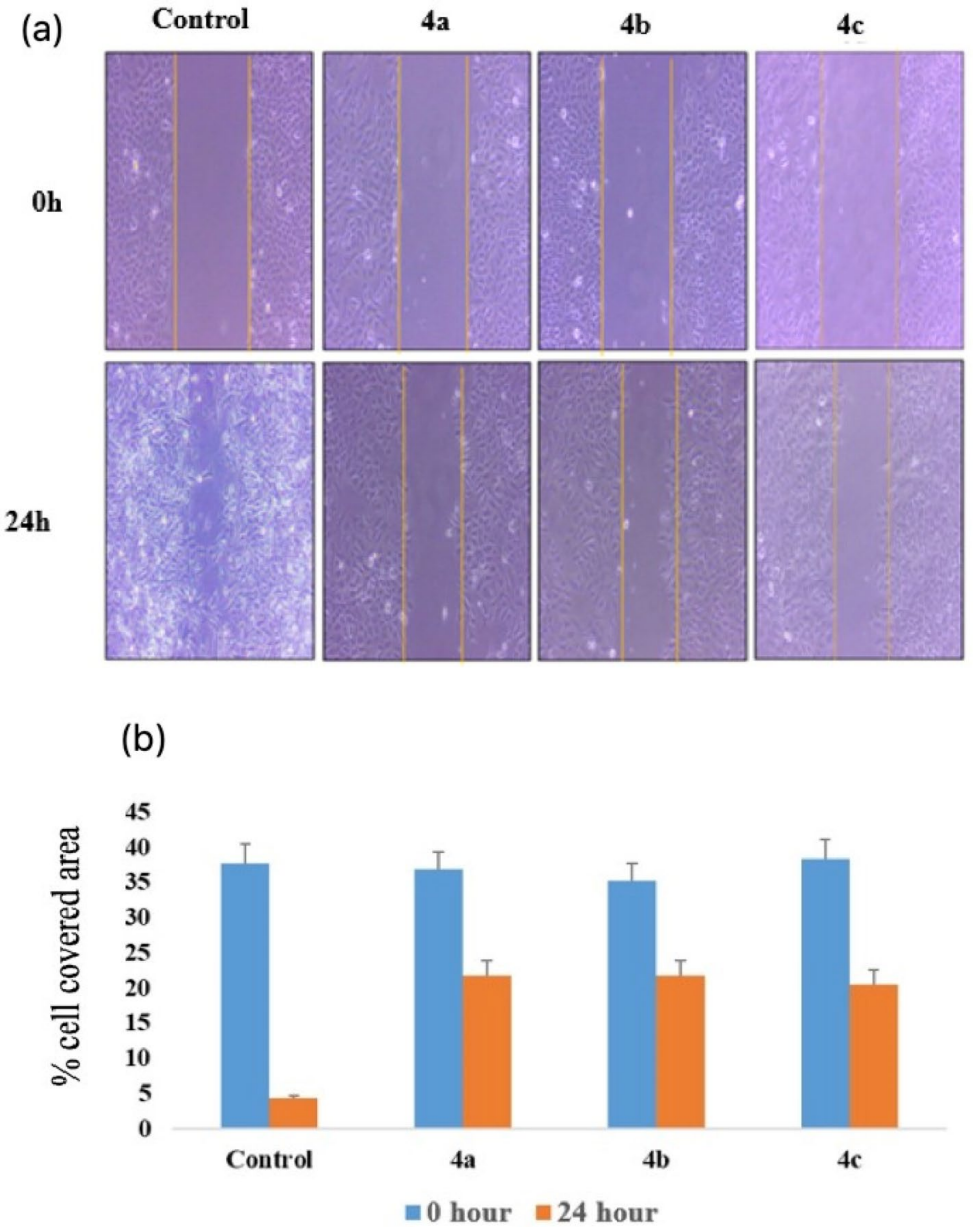
Wound healing assay of compounds **4a**, **4b and 4c** against A549 cells: (**a**) The inhibitory effect of compounds **4a**, **4b** and **4c** on A549 cell migration detected by wound healing assay. (**b**) Representative bar graph showing the cell covered area (%) at 0 and 24 h time intervals after treatment with indicated concentration of compounds **4a**, **4b** and **4c**.

Migration of cells is directly associated with tumor progression and metastasis of cancerous cells. Therefore, wound healing assay was performed to evaluate the migration potential of A549 cells after treatment with compounds **4a**, **4b** and **4c**. An artificial scratch or wound was created on the monolayer of A549 cells using a 200µL sterile pipette tip. The scratch surface was then treated with 30 µM of compounds **4a**, **4b** and **4c** respectively and cells were allowed to migrate. The images of cells to fill up the artificial scratch or wounds were photographed at 0 and 24 h after the treatment and the responses were visualized using phase contrast microscopy. The results obtained revealed a significant suppression in the migration of A549 cells after treatment with compound **4a**, **4b** and **4c** in the wounded area compared to the control group (Fig. 8).

### Nuclear morphological change and nuclear blebbing

**Figure 9:**
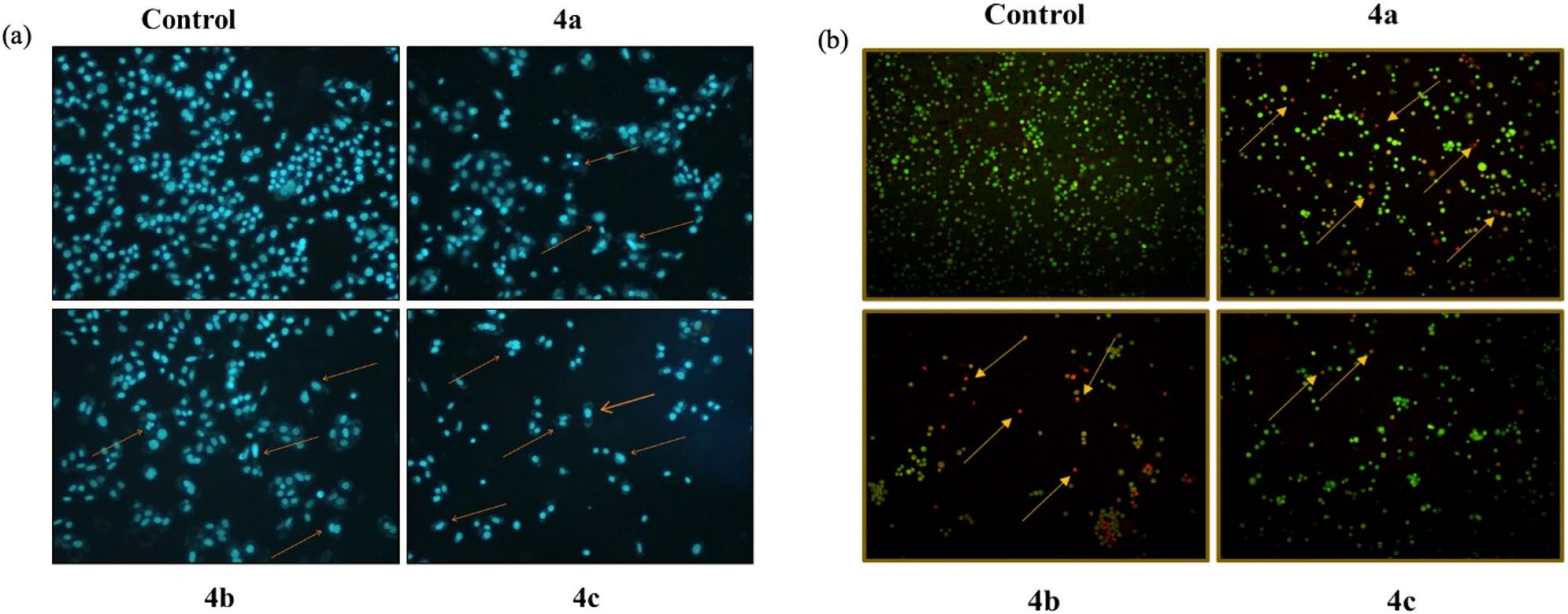
(**a**) DAPI staining of A549 cells after treatment with compounds **4a**, **4b** and **4c**. (**b**) AO/EB dual staining of A549 cells after treatment with compounds **4a, 4b** and **4c**.

A549 cells were treated with compound **4a**, **4b**, **4c and** morphological changes were observed after nuclear staining with 4′,6‐diamidino‐2‐phenylindole (DAPI). The results revealed that, cells lost their polyhedral shape and shrunk (blebbing) in comparison with control cells. The active compounds (**4a**-**c**) exposed cells exhibited chromatin condensation and nuclear fragmentation and margination of nucleus. Morphological results revealed that compound **4a**-**c** reduced cell viability and induced apoptosis in A549 cells (Fig. 9a).

### Early and late apoptotic cells in A549 lung cancer cell line

Apoptosis inducing potential of newly synthesized compounds **4a**, **4b** and **4c** were examined through acridine orange/ethidium bromide (AO/EB) dual staining assay. The results obtained demonstrated that number of apoptotic cells enhanced after treatment of A549 cells with compounds **4a**-**c** as compared with control. An early and late apoptotic along with necrotic cells were also observed in active compounds (**4a**-**c**) treated A549 cells (Fig. 9b). It was concluded that AO/EB staining reduced the antiproliferative activity of compounds **4a**, **4b** and **4c** against A549 cells and it is mediated via induction of apoptosis.

### Acute oral toxicity

Rats were administered compounds **4b** and **4c** at a dose of 500 mg/kg of body weight. No instances of mortality were observed during the course of the experiment. Consequently, it can be concluded that the lethal dose (LD_50_) of the tested compounds is greater than 500 mg/kg body weight. Therefore, the studied compounds **4b** and **4c** displayed an LD_50_ range of > 500 to < 2000 mg/kg, as recommended by the Organization for Economic Co-operation and Development (OECD).

### Cardiomyopathy and hepatotoxicity studies

**Figure 10:**
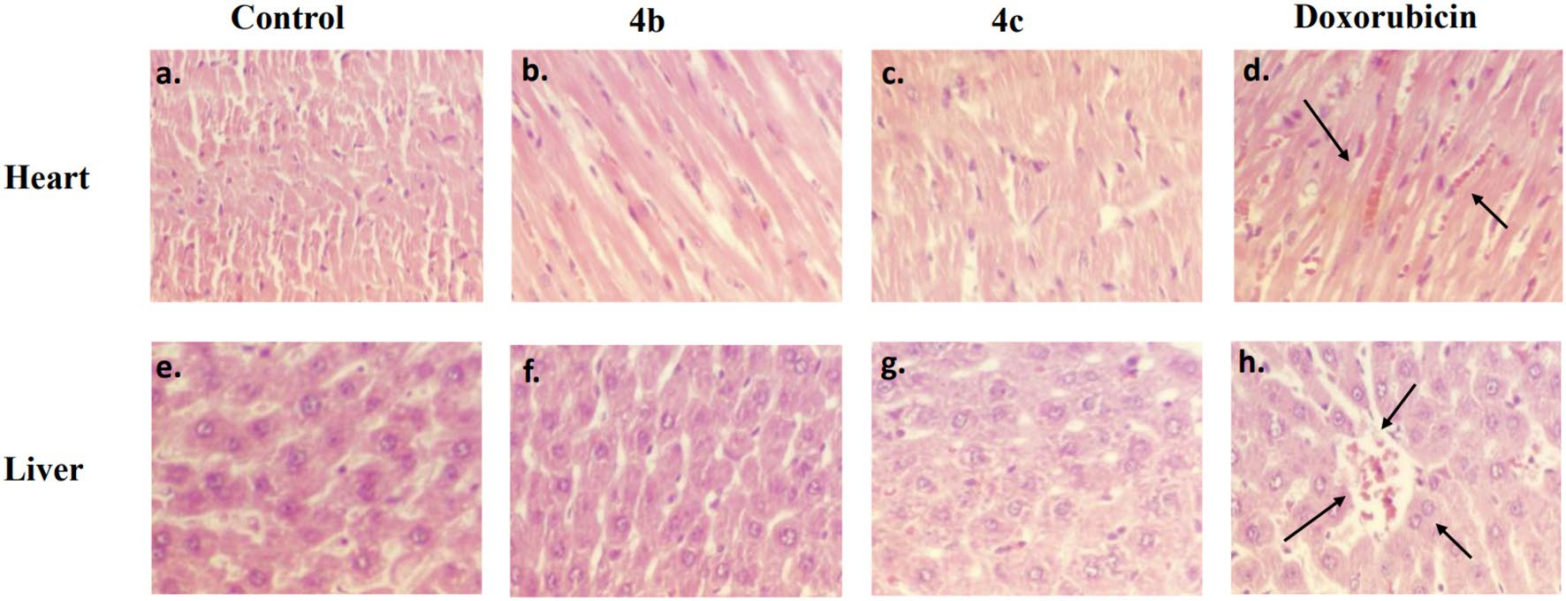
Histopathological images showing the effect of compounds **4b**, **4c** and doxorubicin on the heart (**a**–**d**) and liver (**e**–**h**) tissues compared to the control group. Photomicrograph shows the effect of control, (**b)** and (**c)** a normal architecture of cardiomyocytes, myofibrils, and cardiac fibres with no sign of pyknotic nuclei. (**d**) Signs of cardiomyopathy, loss of cardiac fibrils, myofibrils, and vacuolation can be seen after treatment with doxorubicin. Photomicrograph of control, (**f)** and (**g)** shows normal architecture of hepatocytes, whereas (**h)** shows abnormal hepatocytes after treatment doxorubicin.

Acute toxicity study of most potent compounds **4b** and **4c** were performed. Doxorubicin is used as standard drug and sunflower oil as vehicle control as it is known to inducing cardio and hepatotoxicity in rats. The results obtained from the toxicity study revealed that animals treated with compounds **4b** and **4c** at a dose of 500 mg/kg of body weight does not cause any mortality in any group of animals. At the end of observation period, it was noted that all the animals in the treatment groups survived and there was no severe toxicity and behavioural changes in the treated group animals. From these findings we have concluded that lethal dose (LD_50_) of tested compounds **4b** and **4c** is > 500 mg/kg body weight. Both compounds **4b** and **4c** exhibited oral LD_50_ of > 500 to < 2000 mg/kg which is recommended by OECD (Fig. 10).

### Anti-inflammatory activity

#### Bovine serum albumin (BSA) assay

The synthesized compounds, **4a-g** and **5a-d** were assessed for their anti-inflammatory potential using the protein albumin denaturation inhibition assay. The results obtained are summarized in (Table 2). All the compounds exhibited notable activity, ranging from 62.08% to 84.94%, when compared to the standard drug diclofenac sodium, which demonstrated 84.57% inhibition of denaturation. Based on the data obtained, an analysis of the structure–activity relationship revealed that compounds substituted with electron withdrawing groups at **R** exhibited potent inhibitory activity. Compound **4a**, **4b** and **4c** substituted with flouro, chloro and bromo at the para position of N-phenyl ring attached to thiazolidinone moiety displayed 82.12%, 84.94% and 83.64% of denaturation inhibition respectively. Compounds **5b** and **5c** substituted with bromo and chloro group at **R** position displayed slightly reduced activity showing 76.58% and 79.93% inhibition. However, compound **5c** and **5d** substituted with 4-methyl and 4-methoxy groups at **R** position displayed poor activity with 65.28%, and 59.85% inhibition respectively.

**Table ﻿2:** *In-vitro* anti-inflammatory activity (BSA denaturation inhibition assay).

Compound	R	BSA denaturation inhibition assay	
		Mean absorbance ± SEM	% inhibition of denaturation
**4a**	4-F	0.292 ± 0.001	82.12
**4b**	4-Cl	0.329 ± 0.001	84.94
**4c**	4-Br	0.332 ± 0.006	83.64
**4d**	2,4 di Cl	0.326 ± 0.002	81.97
**4e**	4-NO_2_	0.289 ± 0.012	61.15
**4f**	4-CH3	0.288 ± 0.002	60.59
**4g**	4-OCH_3_	0.291 ± 0.011	62.08
**5a**	4-Cl	0.323 ± 0.002	79.93
**5b**	4-Br	0.317 ± 0.003	76.58
**5c**	4-CH_3_	0.314 ± 0.003	65.28
**5d**	4-OCH_3_	0.287 ± 0.004	59.85
**Diclofenac sodium**	–	0.331 ± 0.002	84.57

### Rat paw edema inhibition assay

The anti-inflammatory activity of the selected compounds **(4a-d and 5a-b),** which showed significant BSA denaturation inhibitory activity was further evaluated for their *in-vivo* inflammatory activity. The compounds and the standard, diclofenac sodium were tested at an oral dose of 0.0314 mmol/kg. The percentage inhibition was calculated after both 3 and 4 h, and since it was found to be more after 4 h, this was made the basis of discussion. The tested compounds showed anti-inflammatory activity ranging from 67.78% to 81.34%, whereas standard drug diclofenac sodium showed 80.32% inhibition after 4 h (Table 3). It was noted that compounds having 4-chloro **(4b),** 4-bromo **(4c)** and 4-fluoro **(4a)** groups at 4th position of N-phenyl ring attached to thiazolidinone moiety displayed excellent activity exhibiting 81.34%, 80.17% and 79.45% inhibition respectively. When 4-chloro (**4b**) group was replaced by 2,4 di chloro group (**4d**) there was sharp decrease in the activity 67.78%. Compounds **5a** and **5b** having 4-chloro and 4-bromo groups at R position of phenyl ring showed slightly reduced (76.24 and 74.78% respectively) anti-inflammatory activity. Thus, it was observed that presence of the electron withdrawing groups in the phenyl ring attached to thiazolidinone moiety improves both anticancer and anti-inflammatory activity. These compounds were also evaluated for their *in-vivo* ulcerogenic and lipid peroxidation activity.

**Table 3:** *In-vivo* anti-inflammatory activity of compounds **4a**, **4b**, **4c**, **4d**, **5a**, **5b** and standard drug diclofenac sodium.

Compound	Increase in Paw volume SEM	% inhibition	Activity relative to standard drug
**4a**	0.141 ± 0.025	79.45	98.91
**4b**	0.128 ± 0.018	81.34	101.27
**4c**	0.136 ± 0.014	80.17	99.82
**4d**	0.173 ± 0.027	67.78	84.39
**5a**	0.163 ± 0.018	76.24	94.92
**5b**	0.133 ± 0.008	74.78	93.10
**Control**	0.686 ± 0.064	–	–
**Diclofenac sodium (Standard drug)**	0.135 ± 0.008	80.32	100

Data were analysed by unpaired student,s *t-test* for n = 6.

### Structure–activity relationship

**Figure 11:**
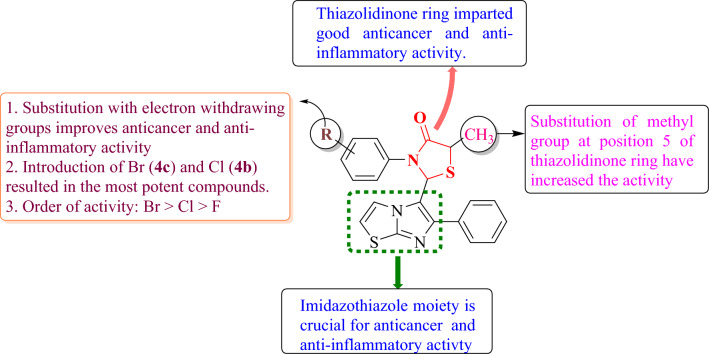
SAR analysis of imidazothiazole-thiazolidinone derivatives.

The structure–activity relationship (SAR) studies of the hybrid molecules containing an imidazothiazole and thiazolidinone moieties and a substituted phenyl ring is depicted in Fig. 11. The synthesized derivatives (**4a-g**, **5a-d**) were subjected to SAR analysis, which revealed that the substitution at *para* position of N-phenyl ring plays a crucial role in determining the anticancer and anti-inflammatory activities. It was observed that the presence of electron-withdrawing groups (Br, Cl, and F) at *para* position of the phenyl ring of thiazolidinone moiety showed significantly enhanced cytotoxicity as compared to the electron donating groups (NO_2_ and OCH_3_). Furthermore, series **4a**-**g** were found to be more potent due to the presence of a methyl group at the 5th position of thiazolidinone moiety when compared to series **5a**-**d** in which methyl group is absent. The presence of methyl group increases the activity due to increase in the lipophilicity and improved membrane penetration. Electron donating inductive effect of the methyl group also leads to differential receptor binding. Therefore, series **5a**-**d** due to absence of a methyl group showed lower activity in comparison to series **4a**-**g**. Thus, it was concluded that compounds substituted with electron-withdrawing groups at *para* position of N-phenyl ring and having a methyl group at 5th position of thiazolidinone moiety were found to be more active.

## Ulcerogenic activity

The Compounds **(4a**-**d**, **5a-b)** which were found potent as anti-inflammatory agents were further screened for their ulcerogenic risk. The compounds and the standard drug were tested at an oral dose of 0.0942 mmol/kg. All the compounds showed reduced ulcerogenic potential and exhibited significant reduction in the severity index (SI 0.416 ± 1.053 to 1.000 ± 0.258) in comparison to standard drug diclofenac sodium (SI 1.667 ± 0.105). Compound **4b** having significant anti-inflammatory activity also showed maximum reduction in severity index (SI 0.416 ± 1.053) (Table 4).

**Table 4:** Ulcerogenic potential and lipid peroxidation studies of compounds **4a**-**d**, **5a**-**b** and standard drug.

Compounds	Mean ulcer severity index ± SEM	Lipid peroxidation Nmol MDA/100 mg tissue
**4a**	1.167 ± 0.210	5.98 ± 0.114
**4b**	0.416 ± 0.153	4.12 ± 0.159
**4c**	0.500 ± 0.182	5.37 ± 0.060
**4d**	1.250 ± 0.281	6.16 ± 0.135
**5a**	1.083 ± 0.238	5.75 ± 0.102
**5b**	1.000 ± 0.258	5.68 ± 0.071
**Control**	0.000	3.33 ± 0.093
**Diclofenac Sodium**	1.667 ± 0.105	6.78 ± 0.073

## Lipid peroxidation

The compounds screened for ulcerogenic potential were also analysed for lipid peroxidation. Lipid peroxidation is measured as nmolMDA/100 mg of gastric mucosa tissue. The tested compounds (**4a-d, 5a-b)** exhibited significant reduction in lipid peroxidation in the range of 4.12 ± 0.159 to 6.16 ± 0.135 nmolMDA/100 mg tissue as compared to standard drug diclofenac sodium which displayed lipid peroxidation of 6.78 ± 0.073 nmolMDA/100 mg tissue. Thus, it was concluded that protection of gastric mucosa might be related to the inhibition of lipid peroxidation.

### Molecular docking studies

The XP (extra precision) mode of docking methodology was employed and done at default parameters, in which OPLS_2005 force field was used. The protein was kept rigid while the ligand was permitted to be flexible in the docking protocol. Each ligand was permitted to fit in the ligand bind domain (LBD) i.e. site where erlotinib was located and the best poses along with the wide range of dock scores were then obtained.

### Structure based computational studies (docking and binding energy calculations)

**Figure 12:**
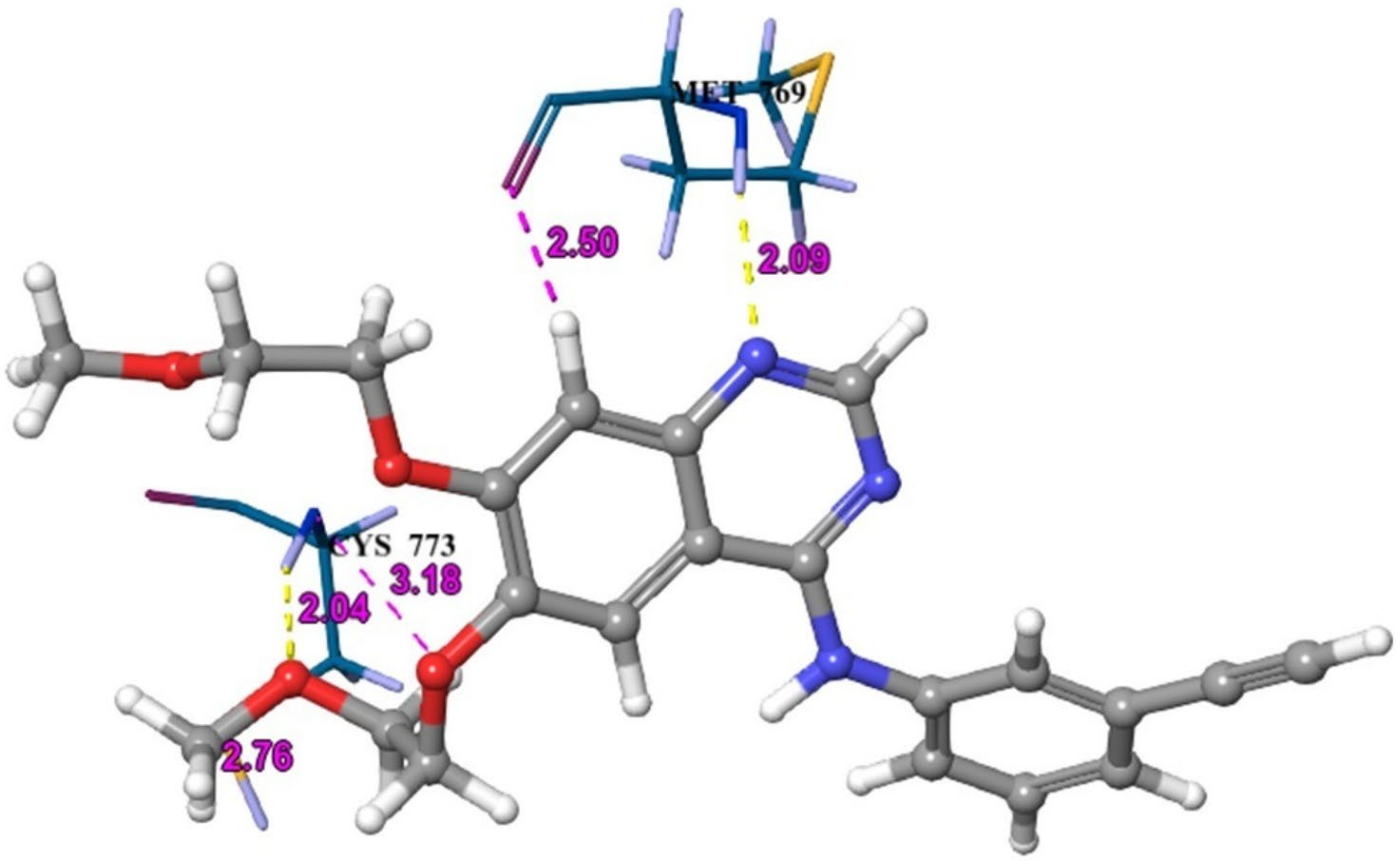
Protein–ligand interaction between 1M17-Erlotinib.

**Figure 13:**
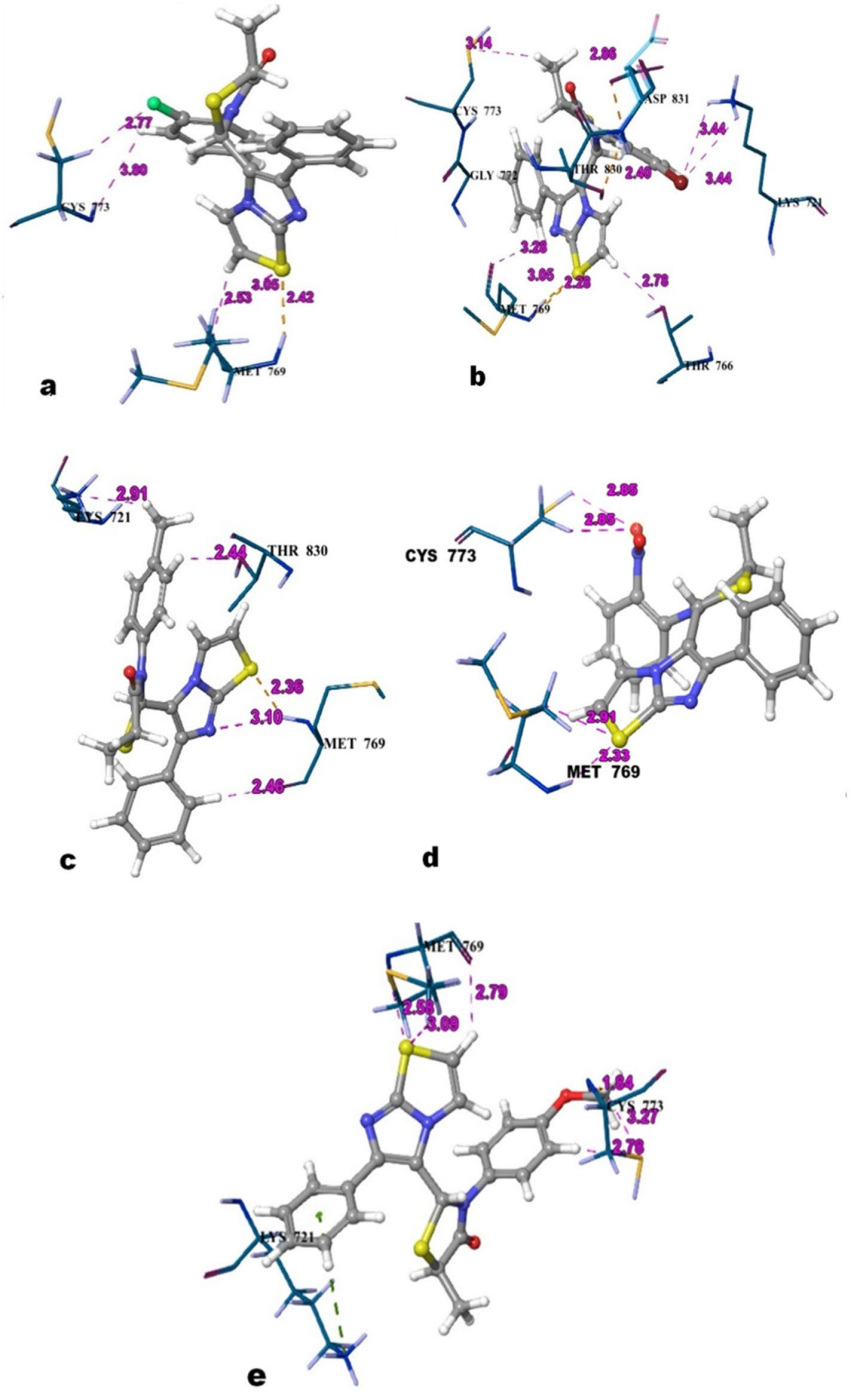
3-Dimensional Protein–ligand contacts between the EGFR (PDB ID 1M17) and best compounds (**a**) **4a** (**b**) **4c** (**c**) **4f** (**d**) **4g** (**e**) **4e**.

**Figure 14:**
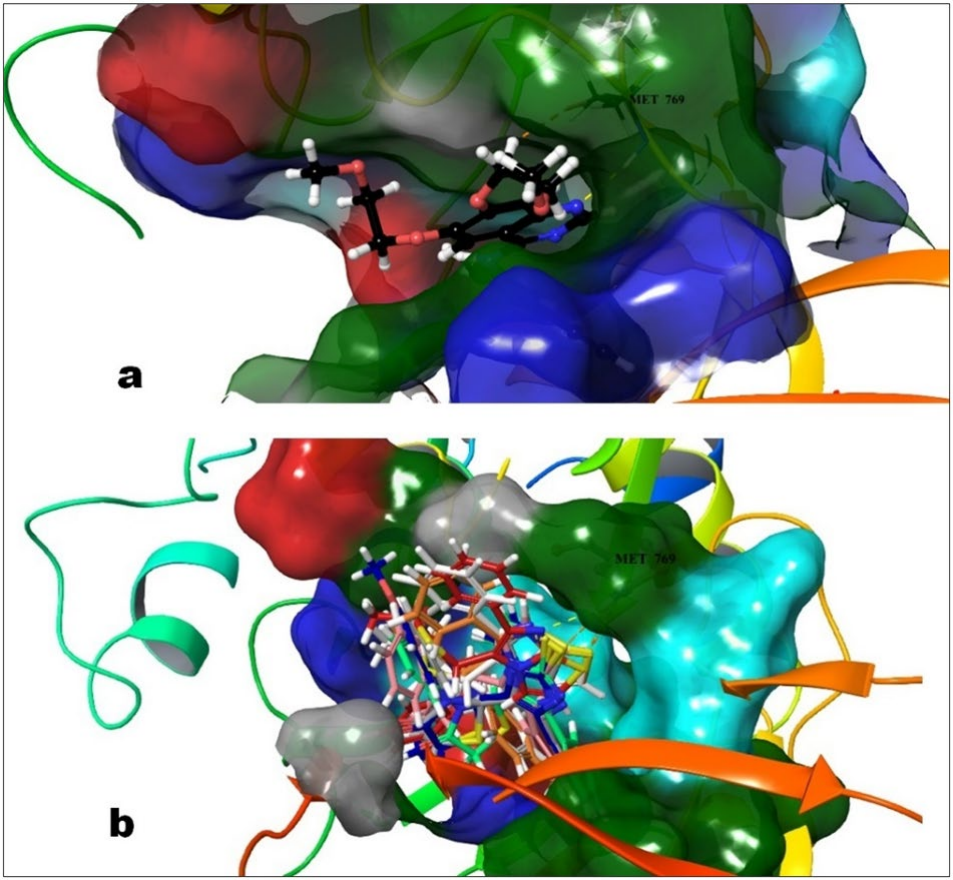
(**a**) The orientation of erlotinib in the ligand binding domain of 1M17; (**b**) The fitness of best oriented compounds in the erlotinib site (**4b**: Blue; **4a**: White; **4c**: Pink; **4d**: Orange; **5b**: Red; **5c**: Spring Green).

The structure based computational approach includes the results of docking as well as the calculated binding energy of the all-synthesized compounds along with the reference i.e. erlotinib with the EGFR protein and cumulated in terms of their dock score, binding energy as and protein–ligand interactions. Erlotinib, the co-crystalized ligand showed the dock score and binding energy and − 61.689 kcal/mol respectively whereas the compounds showed a range of dock score as well as binding energy which was − 3.4 to − 6.7 kcal/mol and − 33.3 to − 62.1 kcal/mol respectively. The compounds having comparable binding energies and P-L interaction profiles have been tabulated (Table 5) and 3-dimentional P-L interactions have been depicted in Figs. 12 and 13. The chief interaction Met769 has been observed and its significance also reported for erlotinib in the literature. The orientation of erlotinib and fitness of best oriented compounds were shown in Fig. 14. The compounds **4c** and **4a** exhibited the hydrogen bonding with Met769, Cys773 as erlotinib while **4b** doesn’t exhibited with Cys773. Other interactions in **4c** such as Thr830, Thr766, Lys721, Asp831 and in **4b** such as Thr830, Lys721 provided the extra stability to the protein ligand complex. Based on the docking scores, and P-L interaction profiles, compounds **4c**, **4a** and **4f** were selected for further evaluation of protein ligand complex stability using 100 nano seconds of Molecular Dynamics Simulation.

**Table 5:** Best compounds with their codes based on the anticancer activity, binding energy, dock scores along with the interactions in docking.

Compounds	Dock score (Kcal/mol)	MM-GBSA binding energy (Kcal/mol)	P-L interactions (H: Hydrogen bond and π: pi–pi interaction)
**4a**	− 5.474	− 40.226	Met769(H), Cys773(H)
**4b**	− 5.842	− 62.117	Met769(H), Lys721(H), Thr830(H)
**4c**	− 5.719	− 40.199	Met769(H), Cys773(H), Thr830(H), Thr766(H), Lys721(H), Asp831(H)
**4d**	− 6.110	− 40.768	Met769(H), Thr830(H), Lys721(H, π)
**4e**	− 6.716	− 33.561	Met769(H), Cys773(H)
**4f**	− 6.716	− 33.561	Met769(H), Thr830(H), Lys721(H, π)
**4g**	− 5.835	− 49.218	Met769(H), Cys773(H), Lys721(π)
**5a**	− 6.409	− 38.449	Met769(H), Cys773(H), Lys721(π)
**5b**	− 5.759	− 34.922	Met769(H)
**5c**	− 3.431	− 33.380	Met769(H), Lys721(H), Phe669 (H)
**5d**	− 4.981	− 38.670	Met769(H)
**Erlotinib**	− 9.083	− 61.689	Met769(H), Cys773(H)

### MD simulation of protein–ligand complex

**Figure 15:**
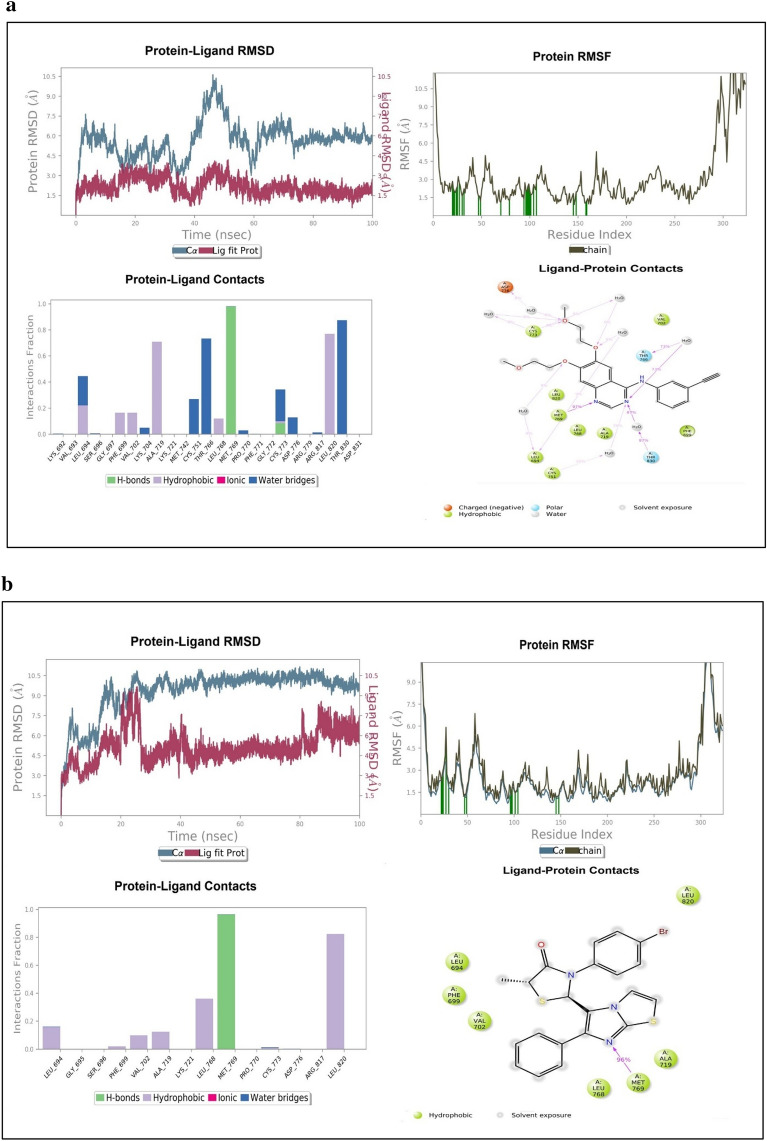

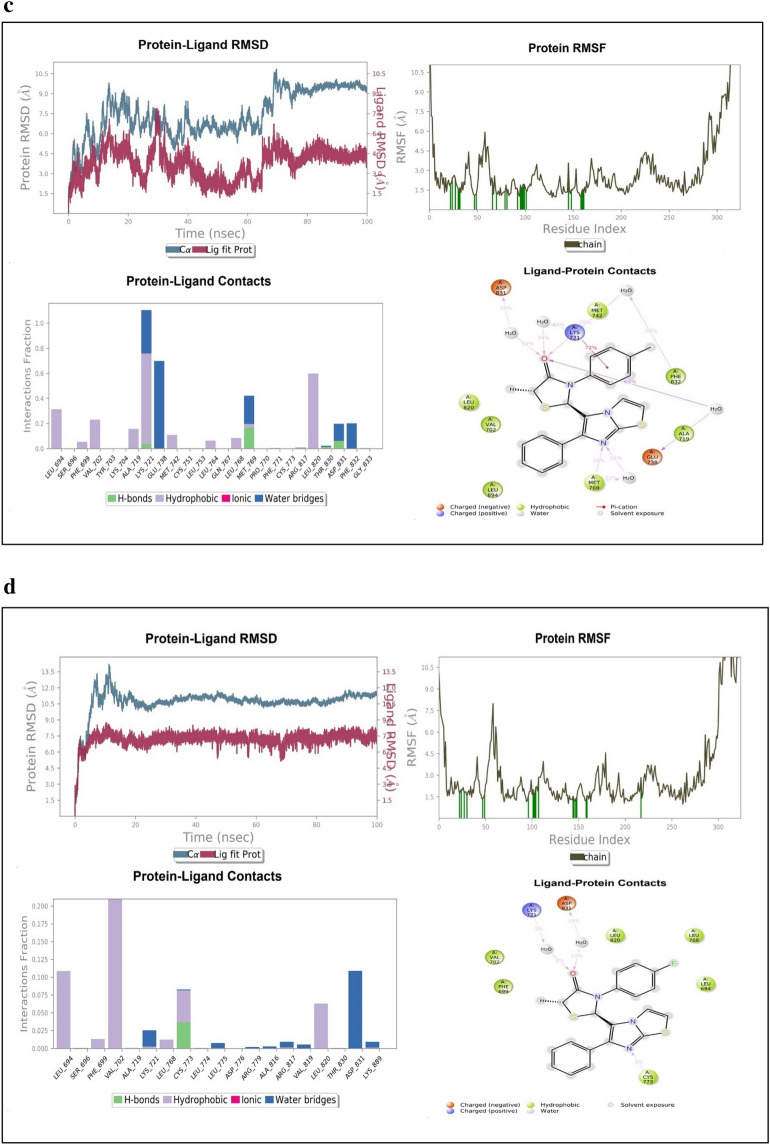
(**a**) MD Simulation studies and Protein–ligand complex patterns between 1M17-Erlotinib. (**b**) MD Simulation studies and Protein–ligand complex patterns between 1M17-4c. (**c**) MD Simulation studies and Protein–ligand complex patterns between 1M17-4f. (**d**) MD Simulation studies and Protein–ligand complex patterns between 1M17-4a.

**Figure 16:**
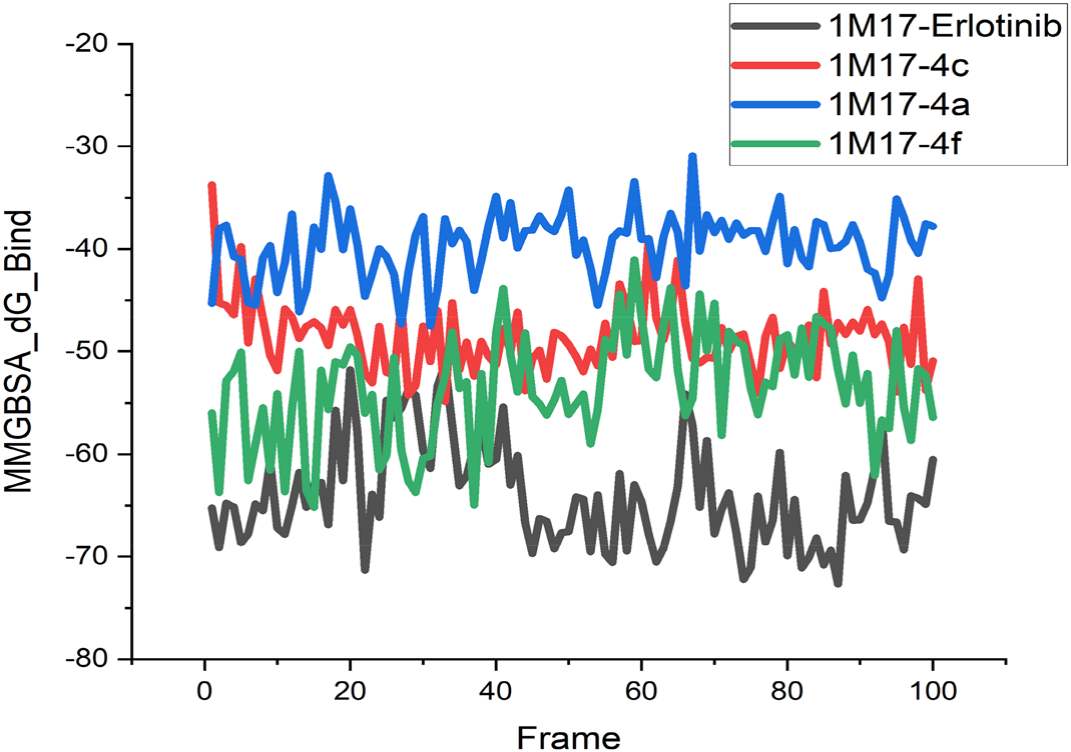
Post-MD Simulation calculated thermal binding energy.

Diverse aspects were evaluated in MD simulation examination, which mainly included the stability strength of protein–ligand by analysing root mean square deviation (RMSD), root mean square fluctuation (RMSF) of backbone and side chain respectively along with the interaction fraction patterns of ligand within the cavity with the duration in 100 ns. The different RMSD value of protein backbone was found in all the complexes in which 1M17-Erlotinib as reference was recorded up to 4.8 Å from 0 to 20 ns, after 20 ns it was approximately at 6–6.5 Å and thereafter, it was average stabilized approximately up to 5.7 Å. In the RMSF trajectory, most of the residues interacted and showed stabilization approximately at 3 Å. Among all the three synthesized derivatives, the complex of 1M17-4c showed a stable RMSD which is recorded approximately at 8 Å throughout the simulation which had greater value of fluctuations but found much consistent throughout the simulation as compared to the reference ligand i.e. erlotinib. RMSF was also found stable around 3 Å as similar to erlotinib. The depiction of RMSD and RMSF of all complexes have been shown in Fig. 15a–d. The interaction fraction patterns have been also obtained in the 100 ns of MD simulation in which erlotinib showed hydrogen bonding with residue Met769 during 97% of the simulation run while Thr830 and Thr766 showed hydrogen bonding through water bridging and have a duration of 87% and 73% of the simulation run respectively. Other interactions which stabilized the ligand in the pocket were also recorded in which Leu820 and Ala719 maximally interacted and formed hydrophobic bonds up to 70–80% of the simulation run. The interaction patterns of erlotinib in the docking simulation was also found the same, as Met769 showed hydrogen bonding having 2.50 Å and 2.19 Å length, which is the key amino acid residue for the inhibition of EGFR. All three compounds i.e., **4a**, **4c** and **4f** showed favourable RMSD and RMSF fluctuation of protein ligand complexes based on the P-L interactions of complexes. Compound **4c** have promising interaction patterns, which was found similar to the erlotinib in which Met769 was interacted 96% throughout the 100 ns simulation while Leu820 and Leu768 interacted hydrophobically around 80% and 38% respectively. While, compound **4a** had not been interacted with Met769 and compound **4f** was found up to 40% interaction with key residue. The docking simulations of Compound **4c** also showed hydrogen bonding with Met769 having 2.28 Å and 3.28 Å bond length. Compound **4a** and **4f** showed interaction patterns with the Met769 but they were not found the similar bonding in the MD simulation studies as compared to the erlotinib and compound **4c**. After 100 ns MD simulation, the binding energy analysis was performed on all protein–ligand complexes using MM-GBSA method. It was found that 4f-1M17 showed relatively lower energy as compared to all P-L complexes except erlotinib-1M17 (reference). Among all complexes, 4c-1M17 was found consistent and stable in the graph so it is inferred that **4c** stabilized the overall conformational dynamics of with 1M17 protein better than 4f-1M17 complex which showed the fluctuations in the conformational energy analysis (Table 6, Fig. 16).

**Table 6:** Post-MD Simulation calculated thermal binding energy, average RMSD and RMSF.

Compd. No	P-L Complex	ΔG _binding_ = Total binding energy (kcal/mol)	Average RMSD (Å)	Average RMSF (Å)
Erlotinib	Erlotinib-1M17	− 64.0373 ± 0.500	5.68577	3.720701
4a	4a-1M17	− 39.5767 ± 0.313	10.65819	3.070343
4c	4c-1M17	− 48.6166 ± 0.339	9.293854	3.423451
4f	4f-1M17	− 53.5654 ± 0.522	7.450217	4.845028

The results retrieved from molecular docking and MD simulation studies has been closely comparable to the biological activity of compound **4a**, **4c** and **4f** Compound **4c** showed the promising anticancer activity against EGFR (IC_50_ 18.35 ± 1.25 µM) and A549 cancer cell line (IC_50_ 10.74 ± 0.40 µM) as lead compound, which showed similar interactions and also duration of interaction as erlotinib i.e. 96% and 97% respectively as compared to the compound **4a** and **4f** in the MD simulation followed by the molecular docking.

## Conclusion

A new series of imidazothiazoles coupled with thiazolidinone moiety were designed and synthesised in multiple steps by reagent-based approach and evaluated for their EGFR kinase inhibitory, anticancer and anti-inflammatory activities. All the synthesized derivatives were evaluated for cytotoxicity against three cancer cell lines A549, MCF7, HCT116 and one normal human embryonic kidney cell line HEK293 which displayed moderate to potent inhibitory activities. Among all the tested compounds, **4c** demonstrated broad‐spectrum anticancer activity with excellent inhibitory potency and showed an IC_50_ value 10.74 ± 0.60 and 18.73 ± 0.88 µM against the cancer line A549 and MCF7 respectively. Compound **4c** was also found to be less toxic than the standard drug erlotinib (IC_50_ 80.15 ± 1.30 µM) against HEK293 cell line and its IC_50_ value was found to be 96.38 ± 1.79 µM which indicated lesser cytotoxicity for healthy cell. Furthermore, compound **4c** inhibited EGFR at 18.35 ± 1.25 µM concentration in comparison to erlotinib as standard drug (06.12 ± 0.92 µM). Moreover, **4c** was also shown to induce apoptosis in A549 cancer cell as evidenced by DAPI and AO/EB dual staining assay. Cardiomyopathy studies showed normal cardiomyocytes with no marked sign of pyknotic nucleus of compound **4b** and **4c**. Hepatotoxicity studies of compound **4b** and **4c** also showed normal architecture of hepatocytes. All the synthesized compounds were tested for their *in-vitro* anti-inflammatory activity by using BSA denaturation inhibition assay and on the basis of activity six compounds (**4a-d**, **5a-b**) showing promising results were selected for *in-vivo* anti-inflammatory activity. Compound **4b** and **4c** showed significant anti-inflammatory activity 81.34% and 80.17% whereas standard drug diclofenac sodium showed 80.32% inhibition. Compound **4b** and **4c** also showed reduced ulcerogenic potential and lipid peroxidation. It was interesting to note that compound **4c** showing potent anticancer activity also showed significant anti-inflammatory activity. SAR studies suggested that substitution at *para* position at N-phenyl ring attached to thiazolidinone moiety with electron withdrawing groups is favourable for both anticancer and anti-inflammatory activities. Molecular docking studies and molecular dynamics simulation studies with the EGFR receptor (PDB ID: 1M17) revealed that compounds **4c** possess strong interaction with the active site of the enzyme receptor by forming strong hydrogen bond with Met769 which is an essential key amino acid residue for the inhibition at the receptor. The 100 ns molecular dynamic simulation and MM-GBSA binding energy calculation proved that **4c** validates stability (RMSD and RMSF) with the protein complex along with the interaction patterns. These compounds thus provided a framework and encouraged us to continue further studies to obtain more potent anticancer and anti-inflammatory agent towards the direction of lead-to-lead optimization studies.

## Experimental

### Chemistry

#### General information

All the reagents and solvents were of laboratory grade and were procured from Merck (Darmstadt, Germany) and S.D Fine chemicals (Delhi, India). To check the advancement of reaction and purity of the compounds, thin layer chromatography (TLC) on pre-coated Aluminium sheets (Silica Gel Merck 60 F254), using various solvents systems, such as Toluene: Ethyl Acetate: Formic Acid in ratio of 5:4:1, Benzene: Acetone (ratio of 8:2) and Ethyl Acetate: Hexane (ratio of 6:4), were used. UV lamp and iodine chamber were used for the visualization of TLC spots. Melting points were recorded in open capillaries using Labtronics Digital Auto Melting Points Apparatus (Haryana, India) and are uncorrected. IR spectra were recorded on Perkin-Elmer 1720 FTIR spectrometer (New York, USA). ^1^H NMR (400 and 500 MHz) and ^13^C-NMR spectra (100 and 125 MHz) were obtained on Bruker Avance- instrument, in CDCI_3_ and DMSO-*d6* solvents. Chemicals shifts (δ) measured relative tetramethylsilane (TMS) as an internal standard are expressed in parts per million (ppm) and J values (coupling constant) were measured in Hz. Mass spectra (ESI–MS) were recorded on synapt G2 HDMS (Waters) and presented as m/z. E lemental analyses (C, H and N) were conducted using a CHNS Vario EL III (Elementar Analysen System GmbH, Germany) and the results are within ± 0.4% of theoretical values.

#### General procedure for the synthesis of compounds (4a-g)

An equimolar solution of Schiff base (**3a-g**) (0.01 mol) and thiolactic acid (0.02 mol) was dissolved in 1,4-dioxane (25 ml). Catalytic amount of anhydrous zinc chloride was added to it and the reaction mixture was stirred at room temperature for 16 to 20 h. The progress of the reaction was monitored by TLC. Upon completion, the reaction mixture was poured into crushed ice. The precipitate thus, obtained was filtered, washed with water, dried and recrystallized from ethanol.

#### 3-(4-Fluorophenyl)-5-methyl-2-(6-phenylimidazo[2,1-b] thiazol-5-yl) thiazolidin-4-one (4a)

White solid, yield: 72%, m.p. 165–167 °C; IR (KBr, υmax cm^−1^): 1687 (C = O), 1560 (C=N), ^1^H NMR (500 MHz, DMSO‐*d6*) δ (ppm): 1.19 (3H, d, J = 7 Hz, CH_3_), 3.45 (1H, q, J = 7 Hz, CH), 6.06 (1H, s, CH), 7.13–7.69 (11H, complex m, 9Ar-H & 2 CH=CH), ^13^C NMR (125 MHz, DMSO‐*d6*): 21.26 (CH_3_), 43.18 (CH), 63.93 (CH of thiazolidinones), 112.17, 121.55, 122.72, 124.15, 125.96, 128.55, 128.77, 129.25, 130.25, 135.06, 138.14, 143.12, 145.41, 146.11, 156.72, 171.33; HRMS-ESI m/z: calcd for C_21_H_16_FN_3_OS_2_ [M + H]^+^ 410.0719, found 410.0715; anal. calcd for C, 61.60; H, 3.94; N, 10.26; found: C, 61.71; H, 4.12; N, 10.35.

#### 3-(4-Chlorophenyl)-5-methyl-2-(6-phenylimidazo[2,1-b] thiazol-5-yl) thiazolidin-4-one (4b)

White powder, yield: 78%, m.p. 177–179 °C; IR (KBr, υmax cm^−1^): 1682 (C = O), 1575 (C=N), ^1^H NMR (500 MHz, DMSO-*d*_*6*_) δ (ppm): 1.11 (3H, d, J = 7 Hz, CH_3_), 3.51 (1H, q, J = 7 Hz, CH), 6.06 (1H, s, CH), 7.17–7.69 (11H, complex m, 9Ar-H & 2 CH = CH), ^13^C NMR (125 MHz, DMSO‐*d6*): δ 21.25, (CH_3_), 47.60 (CH), 60.63(CH of thiazolidinones), 112.89, 120.95, 121.10, 121.42, 124.10, 125.44, 125.95, 128.61, 129.39, 129.84, 131.14, 138.34, 145.82, 152.56, 154.34, 173.47. HRMS-ESI m/z: calcd for C_21_H_16_ClN_3_OS_2_ [M + H]^+^ 426.0427, found 426.0423 [M + 2]^+^427.0392; anal. calcd for C, 59.18; H, 3.79; N, 9.87; found: C, 59.21; H, 3.81; N, 9.75.

#### 3-(4-Bromophenyl)-5-methyl-2-(6-phenylimidazo[2,1-b] thiazol-5-yl) thiazolidin-4-one (4c)

Yellow solid, yield: 78%, m.p. 172–174 °C; IR spectra (KBr, υmax cm^−1^): 1672 (C=O), 1568 (C=N), ^1^H NMR (500 MHz, DMSO‐*d6*): δ (ppm): 1.12 (3H, d, J = 7 Hz, CH_3_), 3.22 (1H, q, J = 7 Hz, CH), 6.05 (1H, s, CH), 7.10 -7.98 (11H, complex m, 9Ar-H & 2 CH=CH), ^13^C NMR, 125 MHz DMSO-*d*_*6*_) 20.02 (CH_3_), 42.92 (CH), 66.82 (CH of thiazolidinones), 117.71, 119.72, 120.33, 121.60, 124.58, 124.96, 125.96, 128.17, 128.52, 129.04, 129.39, 130.29, 137.83, 144.41, 144.90, 178.58. HRMS (ESI) m/z: calcd for C_21_H_16_BrN_3_OS_2_ [M + H]^+^ 469.9918 found 469.9915 [M + 2]^+^470.9897; anal. calcd for C, 53.62; H, 3.43; N, 8.93; found: C, 53.57; H, 3.51; N, 9.11.

#### 3-(2,4-Dichlorophenyl)-5-methyl-2-(6-phenylimidazo[2,1-b]thiazol-5-yl)thiazolidin-4-one (4d)

Yellow solid, yield: 67%, m.p. 151–153 °C; IR (KBr, υmax cm^−1^): 1678 (C=O), 1563 (C=N), ^1^H NMR (500 MHz, DMSO‐*d6*): δ (ppm): 1.18 (3H, d, J = 5 Hz, CH_3_), 4.13 (1H, q, J = 5 Hz, CH), 5.02 (1H, s, CH), 7.03–8.27 (10 H, complex m, 8Ar-H & 2 CH=CH), ^13^C NMR (125 MHz, DMSO‐*d6*): δ21.24, (CH_3_) 42.88, (CH), 60.48 (CH of thiazolidinone), 119.87, 120.32, 121.59, 122.04, 123.98, 124.47, 125.96, 128.62, 129.40, 130.01, 138.38, 144.09, 144.41, 155.60, 156.86, 178.56, (C=O); HRMS (ESI) m/z: calcd for C_21_H_15_Cl_2_N_3_OS_2_ [M + H]^+^ 460.0034 found 460.0031 [M + 2]^+^461.0001; anal. calcd for C, 54.79; H, 3.28; N, 9.13; found: C, 54.87; H, 3.37; N, 9.18.

#### 5-Methyl-3-(4-nitrophenyl)-2-(6-phenylimidazo[2,1-b] thiazol-5-yl) thiazolidin-4-one (4e)

Off-white powder, yield: :72%; m.p. 187–189 °C; IR (KBr, cm-1): 1684 (C=O), 1571 (C = N), ^1^H NMR (500 MHz, DMSO‐*d6*) δ (ppm): 1.49 (3H, d, J = 5.2 Hz, CH_3_), 4.12 (1H, q, J = 5.1,Hz CH), 5.02 (1H, s, CH), 7.49- 8.27 (11H, complex m, 9Ar-H & 2 CH=CH), ^13^C NMR (125 MHz, DMSO‐*d6*): 21.67 (CH_3_), 47.62 (CH) 63.25 (CH of thiazolidinones), 114.87, 116.81, 117.92, 119.25, 119.50,120.49, 125.97, 128.11, 128.27, 128.34, 128.57, 129.13, 138.21, 144.24, 150.02, 175.14 (C = O) HRMS (ESI) m/z: Calcd. for C_21_H_16_N_4_O_3_S_2_ [M + H]^+^ 437.0664 found 437.0667; anal. calcd for C, 57.78; H, 3.69; N, 12.84; found: C, 57.89; H, 3.76; N, 11.71.

#### 5-Methyl-2-(6-phenylimidazo[2,1-b] thiazol-5-yl)-3-*p*-tolyl) thiazolidin-4-one(4f)

Yellow solid, yield: 67%, m.p. 155–157 °C; IR (KBr, υmax cm^−1^): 1685 (C=O), 1565 (C=N), ^1^H NMR (500 MHz, DMSO-*d*_*6*_) δ (ppm): 1.47 (3H, d, J = 5.5 Hz, CH_3_), 2.61 (3H, s, CH_3_), 4.11 (1H, q, J = 5.5 Hz, CH), 5.02 (1H, s, CH), 7.42–8.23 (11H, complex m, 9Ar-H & 2 CH=CH), ^13^C NMR (125 MHz, DMSO-*d*_*6*_) δ 17.39, (CH_3_) 21.25 (CH_3_) 47.29, (CH) 66.81, (CH of thiazolidinone) 110.52, 112.89, 115.60 120.95, 121.10, 121.42, 124.10, 125.44, 125.95, 128.61, 129.39, 129.84, 138.34, 145.82, 152.56, 173.47: HRMS (ESI) m/z: Calcd for C_20_H_19_N_3_OS_2_ [M + H]^+^406.0970 found 406.0987; anal. calcd for C,65.16; H, 4.72; N, 10.36; found: C, 65.29; H, 4.84; N, 10.43.

#### 3-(4-Methoxyphenyl)-5-methyl-2-(6-phenylimidazo[2,1-b] thiazol-5-yl) thiazolidin-4-one (4g)

Yellow solid, yield: 67%, m.p. 162–164 °C; IR (KBr, υmax cm^−1^): 1690 (C=O), 1573 (C=N), ^1^H NMR (500 MHz, DMSO-*d*_*6*_) δ (ppm): 1.12 (1H, d, J = 7.5 Hz, CH_3_), 3.45 (1H, q, J = 7.5 Hz, CH) 3.56 (3H, s, OCH_3_), 6.05 (1H, s, CH),6.92 –7.97 (11H, complex m, 9Ar-H & 2 CH=CH), ^13^C NMR (125 MHz, DMSO-*d*_*6*_) 20.91, (CH_3),_ 42.91 (CH), 60.62 (CH of thiazolidinone), 63.24(CH_3_), 114.12, 118.81, 119.02, 120.06, 120.30, 122.88, 125.96, 128.59, 128.96, 130.25, 130.61, 133.90, 144.11, 145.61, 159.81, 173.31: HRMS (ESI) m/z: Calcd for: C_22_H_19_N_3_O_2_S_2_ [M + H]^+^422.0919 found 422.0915_;_ anal. calcd for C, 62.69; H, 4.54; N, 9.97; found: 62.51; H, 4.67; N, 10.08.

#### General procedure for the synthesis of compounds (5a-d)

An equimolar solution of Schiff base (**3a-g**) (10 mmol) and thioglycolic acid (20 mmol) was dissolved in 1,4-dioxane (25 ml). Catalytic amount of anhydrous zinc chloride was added to it. and the reaction mixture was stirred at room temperature for 16 to 20 h. The progress of the reaction was monitored by TLC. Upon completion, the reaction mixture was poured into crushed ice. The precipitate, thus obtained was filtered, washed with water, dried and recrystallized from ethanol.

#### 3-(4-Chlorophenyl)-2-(6-phenylimidazo[2,1-b] thiazol-5-yl) thiazolidin-4-one (5a)

Yellow solid, yield: 78%, m.p. 177–179 °C; IR (KBr, υmax cm^−1^): 1694 (C=O), 1590 (C=N), ^1^H NMR (500 MHz, DMSO‐*d6*): δ (ppm): 3.48 (1H, d J = 6 Hz, thiazolidinone CH_2_), 3.51 (1H, d, J = 6 Hz, thiazolidinone CH_2_), 5.03 (1H, s, CH), 7.31- 8.05 (11H, complex m, 9Ar-H & 2 CH=CH), ^13^C NMR (125 MHz, DMSO*‐d6*): 33.04 (CH_2_) 56.50 (CH), 118.33, 120.02, 120.67, 121.40, 123.33, 124.61, 125.76, 127.10, 127.53, 127.89, 129.95, 130.86, 139.94, 142.29, 153.94, 174.37, HRMS (ESI) m/z: Calcd. for C_20_H_14_ClN_3_OS_2_ [M + H]^+^412.0267 found 412.0283 [M + 2]^+^413.0304; anal. calcd for C, 58.32; H, 3.43; N, 10.20; found: C, 58.45; H, 3.32; N, 10.31.

#### 3-(4-Bromophenyl)-2-(6-phenylimidazo[2,1-b] thiazol-5-yl) thiazolidin-4-one (5b)

Yellow solid, yield: 68%, m.p. 159–161 °C; IR (KBr, υmax cm^−1^): 1686 (C=O), 1581 (C=N), ^1^H NMR (500 MHz, DMSO*‐d6*): δ (ppm): 3.42 (1H, d, J = 7.5 Hz, thiazolidinone CH_2_), 3.45 (1H, d, J = 7.5 Hz, thiazolidinone CH_2_), 5.04 (1H, s, CH), 7.30- 7.68 (11H, complex m, 9Ar-H & 2 CH=CH), ^13^C NMR (125 MHz, DMSO*‐d6*): 34.27 (CH_2_) 55.19 (CH), 116.63, 117.77, 120.11, 121.60, 122.40, 123.99, 125.98, 128.61, 129.34, 132.74, 133.19, 138.32, 145.85, 148.92, 149.34, 178.59, HRMS (ESI) m/z: calcd. for C_20_H_14_BrN_3_OS_2_ [M + H]^+^ 455.9762 found 455.9758 [M + 2]^+^456.9746; anal. calcd for C, 52.64; H, 3.09; N, 9.21; found: C, 52.41; H, 3.17; N, 9.31.

#### 2-(6-Phenylimidazo[2,1-b] thiazol-5-yl)-3-*p*-tolyl) thiazolidin-4-one (5c)

Yellow solid, yield: 67%, m.p. 153–155 °C; IR (KBr, υmax cm^−1^): 1678 (C=O), 1591 (C=N), ^1^H NMR (500 MHz, DMSO*‐d6*) 2.08 (1H, s, CH_3_), 3.37 (1H, d, J = 5.5 Hz, thiazolidinone CH_2_), 3.41 (1H, d, J = 5.5 Hz, thiazolidinone CH_2_), 5.03 (1H, s, CH), 7.10- 7.55 (11H, complex m, 9 Ar–H & 2 CH=CH), ^13^C NMR (100 MHz, DMSO‐*d6*): δ 27.25, (CH_3_) 33.34, (CH_2_) 55.54 (CH), 118.34, 120.69, 121.40, 125.78, 127.09, 127.35, 127.47, 128.87, 129.39, 129.58, 130.86, 136.09, 139.38, 145.00, 153.94, 174.38: HRMS (ESI) m/z: Calcd. for C_21_H_17_N_3_OS_2_ [M + H]^+^ 392.0813 found 392.0816; anal. calcd for C: 64.43; H, 4.38; N, 10.73; found: C, 64.57; H, 4.43; N, 10.85.

#### 3-(4-Methoxyphenyl)-2-(6-phenylimidazo[2,1-b] thiazol-5-yl) thiazolidin-4-one (5d)

Brown solid, yield: 65% m.p. 160–162 °C; IR spectra (KBr, υmax cm^−1^): 1689 (C=O), 1594 (C=N), ^1^H NMR (500 MHz, DMSO*‐d6*)) 3.40 (3H, s, OCH_3_), 3.47 (1H, d, J = 7 Hz, thiazolidinone CH_2_), 3.50 (1H, d, J = 7 Hz, thiazolidinone CH_2_), 6.05 (1H, s, CH), 7.12 -7.69 (11H, complex m, 9 Ar–H & 2 CH=CH), ^13^C NMR (125 MHz, DMSO*‐d6*) 33.36 (CH_2_) 46.78 (CH_3)_ 56.23 (CH)_,_ 111.92, 115.11, 123.30, 127.21, 127.35, 127.66, 128.35, 128.59, 128.69, 128.85, 129.85, 131.99, 137.13, 138.47, 143.90, 172.84. HRMS (ESI) m/z: Calcd. for C_21_H_17_N_3_O_2_S_2_ [M + H]^+^ 408.0762 found 408.0759; anal. calcd for C, 61.90; H, 4.21; N, 10.31; found: C, 61.79; H, 4.35; N, 10.46.

## Pharmacological activity assays

### In vitro anticancer assay

Cytotoxicity study of the newly synthesized derivatives **4a-g** and **5a-d** was performed by MTT colorimetric assay method. MTT assay is one of the explored and reliable methods to evaluate the cytotoxicity of compounds; it reveals that cell viability increases with an increase in absorbance whereas inhibition of cells decreases. Compounds were tested against selected cancer cell lines namely A549 (Lung), MCF-7(Breast) HCT116 (Colon), normal human embryonic kidney cell line (HEK293) and procured from National Centre for Cell Science Complex (NCCS) Pune University, India. Into a 96 well plate, cells were seeded into each well (3 × 10^3^ cells/ per well), the plate were incubated for 24 h, after that the culture medium was discarded and replaced with fresh medium along with our newly synthesized test compounds in different concentration (10, 20, 40 and 80 µM) followed by incubation for 72 h. Further, the media of each well of the plates were aspirated with the addition of 100 µL MTT solution followed by incubation for the next 5 h at 37 °C. Finally, each well is treated with DMSO (100 µL) and swirled to dissolve the dark crystals of formazan. ELISA reader was used to measure the absorbance at 570 nm. The experiment was repeated three times; Graph pad prism and excel are used to calculate the IC_50_ values^51^.

### In vitro EGFR kinase assay

For all the synthesized compounds, the overall methodology of a spectrophotometric assay for the inhibition of EGFR tyrosine kinase activity was evaluated in human A549 cells, which expressed high levels of EGFR protein. A549 cells (5 × 10^3^ cells/well) were seeded on a 24 well culture plate and cultivated with or without substances at various concentrations (i.e., 500, 100, 25, 10, 5, 1, and 0.2 M for the dose–response study) in triplicate. Cells were then washed with cold PBS, lysed in 0.2 ml/well of HNTG buffer (50 mM HEPES [(4‐(2‐hydroxyethyl)‐piperazineethanesulfonic acid; pH 7.5], 150 mM NaCl, 1.5 mM MgCl, 10% glycerol, 1% Triton X‐100, 0.5 mM sodium fluoride, 2 g/ml aprotinin, 2 g/ml pepstatin A, 1 mM sodium orthovanadate, 2 g/ml leupeptin, 1 mM phenyl methyl sulfonyl fluoride) for 10 min, and the lysates were collected as the supernatant fraction after a 12‐min centrifugation at 15,000 *g*. The specific EGFR concentration was measured by ELISA assay. Briefly, the wells of ELISA plates were coated with supernatant fraction and incubated at 4 °C overnight After washing three times with buffer (20 mM PBS, pH 7.2 containing 0.05% Tween‐20), the wells were blocked with 1% BSA for 2–3 h at room temperature. Further, the plate was washed and then mouse serum at 1,000‐fold dilution was added, followed by washing and incubation with phospho‐EGFR antibody (Invitrogen) at 4 °C overnight. The wells were washed and incubated with substrate solution (o‐phenylenediamine dihydrochloride), for 30 min, and the absorbance read on an ELISA plate reader at 490 nm^52^.

$$\% {\text{ Inhibition }} = \, \left( {{\text{mean OD of untreated group }} - {\text{ mean OD of treated group}}} \right)/{\text{mean OD of untreated group}}) \, \times { 1}00.$$

### Wound healing assay

Wound healing was performed with A549 cells. On six-well plates, cells were seeded at a density of 5 × 10^5^ cells per well and cultured as a confluent monolayer for 24 h. A 2001 pipette sterile tip was used to make an artificial scratch or wound on the monolayer of A549 cells after a 24-h period, and well plates were then washed with PBS to eliminate any non-adherent cells. The therapy was then administered at the appropriate concentrations of chemicals 4c and 4b. A Nikon phase contrast microscope was used to take pictures of the movement of cells over the scratched region at 0 and 24 h in two or three randomly chosen fields^53^.

### Nuclear staining

Cellular nuclear morphology was evaluated by fluorescence microscopy following DAPI staining. The exponentially growing 3 × 104 cells were grown on coverslips in 24‐well tissue culture plates supplemented with complete growth medium (Dulbecco's modified Eagle's media (DMEM)with 10% fetal bovine serum (FBS) making a total volume of 2 ml per well. After 24 h the cells were treated with different drugs with IC_50_ dose and kept in an incubator for 48 h. The cells were washed three times with PBS and subsequently fixed using 4% paraformaldehyde for10 min followed by washing gently with PBS. After washing, the cells were incubated in the dark with freshly prepared DAPI (dissolved in PBS) for 10 min. After incubation, the cells were again gently washed with PBS and the coverslips were mounted on sterile slides using a DPX mounting medium. The prepared slides were observed under a fluorescence microscope (Nikon) having a blue or cyan filter at × 40 magnification^54^.

### Acridine orange‐ethidium bromide staining

A549 cell lines were cultured in six-well plates at 4 × 104 cells/well and then these cells were treated with 1.56 and 12.5 μg mL^−1^ of GO-AuNPs for 48 h respectively. Further, Cells were harvested and stained with AO-EtBr dye mix (1:1 v/v from 100 μg mL^−1^ in PBS) and studied using a fluorescent microscope^55^.

### Animals

All the *in-vivo* experiments were conducted with Albino Wistar rats (female, 150–200 g) procured from the central animal house facility, Jamia Hamdard, New Delhi. Animals were caged individually and maintained at the animal house facility under controlled conditions at a temperature of 23 ± 2 °C in 12 h light and dark cycle with free access to food and water. Euthanasia (sacrifice) of animals was performed by dislocation of the cervical. Animal care, sacrifice protocols, and experiments performed were carried out with the approval of the Institutional Animal Ethics Committee (IAEC), Jamia Hamdard, New Delhi vide Registration No. 173/GO/ReBi/S/2000/CPCSEA **(**Committee for the Purpose of Control and Supervision on Experiments on Animals) and serial no 1806. All methods were carried out in accordance with relevant guidelines and regulations of Organization for Economic Co-operation and Development (OECD). The study is reported in accordance with ARRIVE (Animal Research: Reporting of In Vivo Experiments) guidelines.

### Acute toxicity study

Animals (Wistar rats) procured were divided into three groups randomly, with six rats in each group. Group I and II were used for the single dose treatment of synthesized compounds **4b** and **4c** at a dose of 500 mg/kg of their body weight. Group III is taken as control; all the animals were treated with 0.5 ml of sunflower oil per rat. All the animals were under observation, and after 24 h, mortality of treated animals was recorded. Behaviour, body weight, movement, and survival of the rats were observed for 14 days^56^. All the experiments performed were carried out with the approval of the IAEC, Jamia Hamdard, New Delhi.

### Cardio and hepatotoxicity studies

All the animals procured are randomly divided into four groups, Group I, II, III, and IV. Each rat of Group I and II were treated with synthesized compound **4b** and **4c** orally by using oral gavage with a single dose of 500 mg/kg of body weight. Animals of Group III were treated with the standard drug doxorubicin with a dose of 2 mg/kg of body weight for 5 days. Group IV is taken as control, and a single dose of sunflower oil (vehicle) was administered orally. All the animals were further observed for a period of 14 days, then killed by cervical dislocation method. Vital organs namely the heart, and liver, of each animal were taken out and preserved in 40% freshly prepared formalin. Further transverse sections of each organ were prepared and stained with hematoxylin and eosin. All the prepared slides were observed under an Olympus microscope at × 100 and × 400 magnifications^57^.

## Biological screening

### In-vitro studies

#### BSA denaturation inhibition assay

All the synthesized compounds were evaluated for anti-inflammatory activity by the inhibition of the albumin denaturation technique^58^. The standard drug (Diclofenac sodium) and synthesized compounds were properly dissolved in DMF and then diluted with phosphate buffer saline (pH 7.4). All the test solutions (1 mL, 100 μg/mL) were then mixed with 1 mL of albumin solution (1%) and phosphate buffer saline and then incubated for around 15 min at 27 ± 1 °C. Further, the denaturation of protein was induced by keeping the reaction mixture on the water bath for 10 min and maintaining the temperature at 60 °C. Once the solution gets cooled, the turbidity was measured using UV–Visible spectrophotometer at 660 nm. The experiment was repeated thrice, and then the Percentage inhibition of denaturation was calculated using the formula given below

$$\% {\text{ Inhibition of denaturation }} = \, \left[ {\left( {{\text{A}}_{{\text{t}}} /{\text{A}}_{{\text{c}}} } \right) \, - {1}} \right] \, \times { 1}00$$

where; A_t_ is the mean absorption of the test compound and A_c_ is the mean absorption of the control.

### In-vivo studies

#### Animals

Ten to twelve weeks old adult female Wistar rats, weighing 150–200 g were issued from the Central Animal House Facility of Jamia Hamdard, New Delhi. All the animals were housed in large polypropylene cages and ventilated rooms at 25 ± 2 °C under a 12 h light/dark cycle. Animals were allowed to acclimatize for one week before the study and provided free access to food and water ad libitum. Animal facilities and other areas in contact with laboratory animals were cleaned and disinfected. Experiments performed were carried out with the approval of the Institutional Animal Ethics Committee (IAEC), Jamia Hamdard, New Delhi vide Registration No. 173/GO/ReBi/S/2000/CPCSEA and serial no 1806. All methods were carried out in accordance with relevant guidelines and regulations as per the guidelines of the Committee for the Purpose of Control and Supervision on Experiments on Animals (CPCSEA), national regulatory body for experiments on animals, Ministry of Environment & Forests, India. The study is reported in accordance with ARRIVE (Animal Research: Reporting of In Vivo Experiments) guidelines.

### Anti-inflammatory activity

The anti-inflammatory efficacy of the newly synthesised compounds was assessed using a carrageenan-induced rat hind paw edema technique The animals were divided into groups of six at random, fasted for 24 h before to the experiment, and given free access to water. 0.5% CMC solution was given to the control group only. All the test compounds as well as the reference drug diclofenac sodium were given orally at a dose of 0.0314 mmol/kg. One hour after the administration of the test compounds and the reference drug, 0.1 mL of a 1% carrageenan solution in saline was subcutaneously injected into the subplantar area of the right hind paw of each rat. The right hind paw volume was measured using a Digital Plethysmometer PLM-01 Plus (Orchid Scientifics and Innovatives India Pvt. Ltd., Mumbai, India) at 3 and 4 h after carrageenan therapy. The following calculation was used to compute the percent edema inhibition from the mean effect in the treated and control animals:

$${\text{Percent edema inhibition }} = \, \left( {{\text{Vc}} - {\text{Vt}}/{\text{Vc}}} \right) \, \times { 1}00$$

where Vc denotes the mean increase in paw volume in the control group of rats and Vt denotes the mean increase in paw volume in rats treated with test chemicals. After a 15-day washout period, the same group of rats utilised for the anti-inflammatory activity were employed for the ulcerogenic activity^59^.

### Ulcerogenic activity

The ulcerogenic activity of six most potent compounds and diclofenac sodium were evaluated by using the reported method^60^. Animals were divided into different groups consisting of 6 rats per group. Following oral administration of the test compounds and the standard drug (diclofenac sodium) at a dose of 0.0942 mmol/kg, ulcerogenic activity was assessed. The control group received 0.5% of the carboxymethylcellulose (CMC) solution. Water but not the food was discontinued 24 h before the test compounds were administered. After receiving the medication, the rats were given regular food for 17 h and then sacrificed. The stomach was taken out and opened along its greater curvature before being gently cleansed by dipping it in normal saline and then washing it with distilled water. The mucosal injury/damage was examined thoroughly using a magnifying glass. The mucosal injury in each stomach was scored using the following score system: 0.5: Redness; 1.0: Spot Ulcers; 1.5: Hemorrhagic Streaks; 2.0: Ulcers > 3 but 5, 3.0: Ulcers > 5. The severity index (SI) of stomach mucosal injury was calculated as the mean score of each treated group minus the mean score of the control group.

### Lipid peroxidation

Lipid peroxidation in the gastric mucosa was determined using the reported method by Ohkawa H. et al.^61^. The gastric mucosa of the glandular area was scraped off using glass slides after the stomach had been examined for ulcers. Gastric mucosa was weighed (100 mg) and homogenised in 1.8 cm^3^ of ice-cold solution of KCl (1.15%). The homogenate is further added with 0.2 cm^3^ of 8.1% sodium dodecyl sulphate (SDS), 1.5 cm^3^ of acetate buffer, and 1.5 cm^3^ of 0.8% thiobarbituric acid. After adding all buffers, the mixture was kept for incubation for 60 min over water bath, maintaining the temperature of the water bath at 95ºC. Later, the mixture was allowed to cool and then extracted with a freshly prepared mixture of n-butanol and pyridine (15:1 v/v), the organic layer obtained was centrifuged at 3000 rpm for about 10 min. The organic layer was separated out and visualised on UV–visible spectrophotometer. Concentration was measured using the following formula:

$${\text{Concentration }} = \, \left( {{\text{Absorbance }} \times {\text{ volume }} \times { 1}0{9}} \right) \, \div \, \left( {{1}.{56 } \times { 1}0^{{5}} \times { 1}000} \right)$$

Results obtained were expressed as nmol MDA/100 mg tissue and extinction coefficient (1.56 × 10^5^) cm^-1^ M^-1^.

### Molecular docking studies

Imidazothiazole-thiazolidinone hybrids showed inhibitory potential against the A549 cancer cell line. The 3D Crystal Structure of the protein i.e. epidermal growth factor receptor (EGFR) having PDB id is 1M17, which is retrieved from the protein data bank with a resolution of 2.60 Å for molecular docking through Glide (version 6.9, *Glide, Schrödinger, LLC, New York, NY, 2017)* followed by the ligand as well as protein preparation using Lig-Prep (version 3.6, *Lig-Prep, Schrödinger, LLC, New York, NY, 2017)* and protein preparation wizard, and respectively module of Schrodinger suite of Drug discovery of the version 2017–2 **(***Maestro, Schrödinger, LLC, New York, NY, 2017)*. Pre-preparation of Structure Based Computational studies (Docking and Binding Energy Calculations). The synthesized compounds (ligands) and EGFR protein were prepared, minimized, and optimized using protein preparation wizard using force field OPLS_2005^62^. The target structure (PDB: 1M17) was prepared using addition of hydrogens, missing side chains as well as loops along with the removal of water molecules which were above than 5 Å in chain A. Further, the energy of crystal structure of protein was minimized and optimized. The active site of protein was assigned using the Glide at the co-crystalized ligand i.e. Erlotinib and the 3D grid was generated.

### Binding energy calculations

The relative binding energy of compounds was computed from structural information in bimolecular complexes using Prime module (version 4.2, *Prime, Schrödinger, LLC, New York, NY, 2017)* of Schrödinger software. Prime MMGBSA (Molecular Mechanics, the Generalized Born model and Solvent Accessibility) is a physics-based method that computes the force field energies in implicit solvent of the bound and unbound molecules involved in the binding process. The MM-GBSA binding energy includes protein–ligand van der Waals, electrostatic interactions, ligand desolvation, and internal strain (protein and ligand) energies using OPLS_2005 force field. Synthesized compounds from docking on EGFR (IM17) were subjected to the MM-GBSA analysis separately. During the prediction of binding energies, the active site of protein was set to adjust up to a distance of 10 Å for ligand accordingly. This operation imports pose viewer file of XP docking of protein and ligand complex, which results in ranking of the ligands based on the calculated binding energies^63^.

### Molecular dynamics simulation study

Molecular dynamics simulation studies of the protein–ligand complex were done for the exploration of stability of potential compounds within the cavity, i.e., 4a, 4c, 4f and also compared with the reference compound i.e. erlotinib. The IM17 with erlotinib, 4a, 4c and 4f complexes were solvated using explicit SPC (simple point charge) water molecules in orthorhombic box. The complex with erlotinib, 4a, 4c, 4f was neutralized by adding 51 Na^+^ & 46 Cl^-^ counterions to stabilize the net charge of the all systems. After addition of the solvent and counterions, the prepared system of compounds with protein i.e. 1M17-Erlotinib, 1M17-4c, 1M17-4a and 1M17-4f completed for minimization step that contained 55,092, 55,168, 55,189 and 55,180 atoms respectively. MD simulation of all complexes was performed for a period of 100 ns with a time-step of 2 fs in NPT (constant number of atoms N, pressure P, and temperature T) ensemble at 300 K temperature and 1 bar pressure. Temperature and pressure conditions were maintained by Nose-Hover chain thermostat and Martyna-Tobias-Klein barostat, respectively. MD simulation was accomplished using multistep practice present in Desmond software (version 3.8, *Desmond, Schrödinger, LLC, New York, NY, 2017)*. Recording interval of 1.2 ps was used for energy and 4.8 ps were used for trajectory for every complex. The structural stability and protein–ligand interactions were analysed over a period of 100 ns MD run. Further, for the post processing of MD simulation using *thermal_mmgbsa.py* script was performed of the complexes and the trajectory file that was obtained from the simulation is used by the script, which divides it into several snapshots before running MM-GBSA on each frame and producing the average binding energies of ligand with the protein i.e. 1M17 individually, which consist of Ewald tolerance of 1 × 10^9^ for the long-range smooth particle grid and a short-range limit radius of 9.0 were used to describe columbic interactions^64,65^.

### Supplementary Information

Supplementary Information.

## Data Availability

All data generated or analysed during this study are included in this published article and its Supplementary Information files.

## References

[1] De Silva F, Alcorn J (2022). A tale of two cancers: A current concise overview of breast and prostate cancer. Cancers.

[2] Melosky B, Kambartel K, Hantschel M, Bennetts M, Nickens D, Brinkmann J, Kayser A, Moran M, Cappuzzo F (2022). Worldwide prevalence of epidermal growth factor receptor mutations in non-small cell lung cancer: A meta-analysis. Mol. Diagn. Ther..

[3] Haider K, Rehman S, Pathak A, Najmi AK, Yar MS (2021). Advances in 2-substituted benzothiazole scaffold-based chemotherapeutic agents. Arch. Pharm..

[4] Haider K, Shrivastava N, Pathak A, Dewangan RP, Yahya S, Yar MS (2022). Recent advances and SAR study of 2-substituted benzothiazole scaffold based potent chemotherapeutic agents. Results Chem..

[5] Nerdy N, Lestari P, Fahdi F, Putra ED, Amir SA, Yusuf F, Bakri TK (2022). In silico studies of sesquiterpene lactones from Vernonia amygdalina delile on the expression of EGFR and VEGFR as a new anticancer potential. Pharmacogn. J..

[6] Zaraei SO, Sbenati RM, Alach NN, Anbar HS, El-Gamal R, Tarazi H, Shehata MK, Abdel-Maksoud MS, Oh CH, El-Gamal MI (2021). Discovery of first-in-class imidazothiazole-based potent and selectiveErbB4 (HER4) kinase inhibitors. Eur. J. Med. Chem..

[7] Abourehab MA, Alqahtani AM, Youssif BG, Gouda AM (2021). Globally approved EGFR inhibitors: Insights into their syntheses, target kinases, biological activities, receptor interactions, and metabolism. Molecules.

[8] Dhiwar PS, Purawarga Matada GS, Pal R, Singh E, Ghara A, Maji L, Sengupta S, Andhale G (2023). An assessment of EGFR and HER2 inhibitors with structure activity relationship of fused pyrimidine derivatives for breast cancer: a brief review. J. Biomol. Struct. Dyn..

[9] Merlino GT, Xu YH, Richert N, Clark AJ, Ishii S, Banks-Schlegel S, Pastan I (1985). Elevated epidermal growth factor receptor gene copy number and expression in a squamous carcinoma cell line. J. Clin. Investig..

[10] Cohen P, Cross D, Jänne PA (2021). Kinase drug discovery 20 years after imatinib. Nat. Rev. Drug Discov..

[11] Nawaz F, Alam O, Perwez A, Rizvi MA, Naim MJ, Siddiqui N, Pottoo FH, Jha M (2020). 3′-(4-(Benzyloxy)-phenyl)1′-phenyl-5-(heteroaryl/aryl)3,4-dihydr3, bipyrazole,2-carboxamides as EGFR kinase inhibitors: Synthesis, anticancer evaluation, and molecular docking studies. Arch. Pharm..

[12] Zhang H, Berezov A, Wang Q, Zhang G, Drebin J, Rurali R, Greene MI (2007). ErbB receptors: From oncogenes to targeted cancer therapies. J. Clin. Investig..

[13] Yewale C, Baradia D, Vhora I, Patil S, Misra A (2013). Epidermal growth factor receptor targeting in cancer: A review of trends and strategies. Biomaterials.

[14] Davinder S, Bhupinder A, Kaur G, Jitender B (2016). Review on EGFR inhibitors: Critical updates. Mini-Rev. Med. Chem..

[15] Killock D (2015). Lung cancer: A new generation of EGFR inhibition. Nat. Rev. Clin. Oncol..

[16] Harari PM (2004). Epidermal growth factor receptor inhibition strategies in oncology. Endocr. Relat. Cancer.

[17] Scaltriti M, Baselga J (2006). The epidermal growth factor receptor pathway: A model for targeted therapy. Clin. Cancer Res..

[18] Zhang Y, Zhang W, Hou J, Wang X, Zheng H, Xiong W, Yuan J (2017). Combined effect of tris(2-chloroethyl) phosphate and benzo (a) pyrene on the release of IL-6 and IL-8 from HepG2 cells via theEGFR-ERK1/2 signaling pathway. RSC Adv..

[19] Kalinowski A, Galen BT, Ueki IF, Sun Y, Mulenos A, Osafo-Addo A, Clark B, Joerns J, Liu W, Nadel JA, Cruz CD (2018). Respiratory syncytial virus activates epidermal growth factor receptor to suppress interferon regulatory factor 1-dependent interferon-lambda and antiviral defense in airway epithelium. Mucosal Immunol..

[20] Xu X, Steere RR, Fedorchuk CA, Pang J, Lee JY, Lim JH, Xu H, Pan ZK, Maggirwar SB, Li JD (2011). Activation of epidermal growth factor receptor is required for NTHi-induced NF-kappa B dependent inflammation. PloS One.

[21] Huang BR, Chen TS, Bau DT, Chuang IC, Tsai CF, Chang PC, Lu DY (2017). EGFR is a pivotal regulator of thrombin-mediated inflammation in primary human nucleus pulposus culture. Sci. Rep..

[22] Fang WL, Zhong P, Chen L, Wang L, Zhang Y, Wang J, Wang X, Li Y, Wang J (2016). Liangd EGFR inhibition blocks palmitic acid-induced inflammation in cardiomyocytes and prevents hyperlipidemia-induced cardiac injury in mice. Sci. Rep..

[23] Sbenati RM, Semreen MH, Semreen AM, Shehata MK, Alsaghir FM, El-Gamal MI (2021). Evaluation of imidazo[2,1-b] thiazole-based anticancer agents in one decade (2011–2020): Current status and future prospects. Bioorg. Med. Chem..

[24] Jaitak V, Kaur K (2022). Thiazole and related heterocyclic systems as anticancer agents: A review on synthetic strategies, mechanisms of action and SAR Studies. Curr. Med. Chem..

[25] Andreani A, Cavalli A, Granaiola M, Leoni A, Locatelli A, Morigi R, Rambaldi M, Recanatini M, Garnier M, Meijer L (2000). Imidazo[2,1 -b]thiazolylmethylene- and indolylmethylene-2- indolinones: A new class of cyclin-dependent kinase inhibitors. Design, synthesis, and CDK1/cyclin B inhibition. Anticancer Drug Des..

[26] Ahmed A, Molvi KI, Patel HM, Ullah R, Bari A (2020). Synthesis of novel 2, 3, 5-tri-substituted thiazoles with anti-inflammatory and antibacterial effect causing clinical pathogens. J. Infect. Public Health.

[27] Shareef MA, Sirisha K, Sayeed IB, Khan I, Ganapathi T, Akbar S, Kumar CG, Kamal A, Babu BN (2020). Synthesis of new triazole fused imidazo[2,1-b] thiazole hybrids with emphasis Staphylococcus aureus virulence factors. Bioorg. Chem. Lett..

[28] Kassab RM, Gomha SM, Muhammad ZA, El-Khouly AS (2021). Synthesis, biological profile, and molecular docking of some new bis- imidazole fused templates and investigation of their cytotoxic potential as anti-tubercular and/or anticancer prototypes. Med. Chem..

[29] Koudad M, El Hamouti C, Elaatiaoui A, Dadou S, Oussaid A, Abrigach F, Pilet G, Benchat N, Allali M (2020). Synthesis, crystal structure, antimicrobial activity and docking studies of new imidazothiazole derivatives. Indian Chem. Soc..

[30] Hautkr Z (1979). Corrigendum notice. Mar. Biol..

[31] Clegg W, Jamieson C (2005). Pifithrin-β. Acta Crystallogr. Sect. E Struct. Rep. Online.

[32] Thienpont D, Vanparijs OFJ, Raeymaekers AHM, Vandenberk J, Demoen PJA, Allewijn FTN, Marsboom RPH, Niemegeers CJE, Schellekens KHL, Janssen PAJ (1966). Tetramisole, (R 8299), a new, potent broad spectrum anthelmintic. Nature.

[33] Zhou F, Ge Z, Chen B (2019). Quizartinib (AC220): A promising option for acute myeloid leukemia. Drug Des. Dev. Ther..

[34] Kampa-Schittenhelm KM, Heinrich MC, Akmut F, Döhner H, Döhner K, Schittenhelm MM (2013). Quizartinib (AC220) is a potent second-generation class III tyrosine kinase inhibitor that displays a distinct inhibition profile against mutant-FLT3, -PDGFRA and -KIT isoforms. Mol. Cancer.

[35] Minor RK, Baur JA, Gomes AP, Ward TM, Csiszar A, Mercken EM, Abdelmohsen K, Shin YK, Canto C, Scheibye- Knudsen M, Krawczyk M, Irusta PM, Martín-Montalvo A, Hubbard BP, Zhang Y, Lehrmann E, White AA, Price NL, Swindell WR, Pearson KJ, Becker KG, Bohr VA, Gorospe M, Egan JM, Talan MI, Auwerx J, Westphal CH, Ellis JL, Ungvari Z, Vlasuk GP, Elliott PJ, Sinclair DA, de Cabo R (2011). SRT1720 improves survival and healthspan of obese mice. Sci. Rep..

[36] Pacholec M, Bleasdale JE, Chrunyk B, Cunningham D, Flynn D, Garofalo RS, Griffith D, Griffor M, Loulakis P, Pabst B, Qiu X, Stockman B, Thanabal V, Varghese A, Ward J, Withka J, Ahn K (2010). SRT1720, SRT2183, SRT1460, and resveratrol are not direct activators of SIRT1. J. Biol. Chem..

[37] Deng X, Tan X, An T, Ma Q, Jin Z, Wang C, Meng Q, Hu C (2019). Synthesis, characterization, and biological activity of a novel series of Benzo [4, 5] imidazo [2, 1-b] thiazole derivatives as potential epidermal growth factor receptor inhibitors. Molecules.

[38] Zaraei SO, Sbenati RM, Alach NN, Anbar HS, El-Gamal R, Tarazi H, Shehata MK, Abdel-Maksoud MS, Oh CH, El-Gamal MI (2021). Discovery of first-in-class imidazothiazole-based potent and selective ErbB4 (HER4) kinase inhibitors. Eur. J. Med. Chem..

[39] Gadekar PK, Urunkar G, Roychowdhury A, Sharma R, Bose J, Khanna S, Damre A, Sarveswari S (2021). Design, synthesis and biological evaluation of 2,3 dihydroimidazo [2,1b] thiazoles as dual EGFR and IGF1R inhibitors. Bioorg. Chem..

[40] Shetty NS, Khazi IA (2010). Synthesis, anthelmintic and anti-inflammatory activities of some novel imidazothiazole sulfides and sulfones. Bull. Korean Chem. Soc..

[41] Kryshchyshyn-Dylevych A, Radko L, Finiuk N, Garazd M, Kashchak N, Posyniak A, Niemczuk K, Stoika R, Lesyk R (2021). Synthesis of novel indole-thiazolidinone hybrid strictures as promising scaffold with anticancer potential. Bioorg. Med. Chem..

[42] Ye X, Zhou W, Li Y, Sun Y, Zhang Y, Ji H, Lai Y (2010). Darbufelone, a novel anti-inflammatory drug, induces growth inhibition of lung cancer cells both in vitro and in vivo. Cancer Chemother. Pharmacol..

[43] Jie P, Wang Y, Xiaoyan W, Xixi W, Gaozhi C, Gaozhong C, Xueqian S, Xiuhua Z, Qinqin T (2013). Synthesis and biological evaluation of novel thiazolidinone derivatives as potential anti-inflammatory agents. Eur. J. Med. Chem..

[44] Alexiou P, Pegklidou K, Chatzopoulou M, Nicolaou I, Demopoulos VJ (2009). Aldose reductase enzyme and its implication to major health problems of the 21st century. Curr. Med Chem..

[45] Balzarini J, Orzeszko B, Maurin JK, Orzeszko A (2009). Synthesis and anti-HIV studies of 2-adamantyl-substituted thiazolidin-4-ones. Eur. J. Med. Chem..

[46] Osczczenko P, Holota S, Szewczyk KO, Dudchak R, Bielawski K, Bielawska A, Lesyk R (2022). Thiazolidinone-bearing hybrid molecules in anticancer drug design. Int. J. Mol. Sci..

[47] Liaras K, Fesatidou M, Fesatidou G (2018). Thiazoles and thiazolidinones as COX/LOX inhibitors. Molecules.

[48] Joshi H, Pal T, Ramaa CS (2020). A new dawn for the use of thiazolidinediones in cancer therapy. Expert Opin. Investig. Drugs.

[49] Szychowski KA, Leja ML, Kaminskyy DV, Binduga UE, Pinyazhko OR, Lesyk RB, Gmiński J (2017). Study of novel anticancer 4-thiazolidinone derivatives. Chem. Biol. Interact..

[50] Saliyeva L, Holota S, Grozav A, Yakovychuk N, Litvinchuk M, Slyvka N, Vovk M (2021). Synthesis, the anti- exudative and antimicrobial activity of 6-arylidene substituted imidazo[2,1-b] thiazoles. Appl. Chem..

[51] Haider K, Ahmad K, Najmi AK, Das S, Joseph A, Yar MS (2022). Design synthesis, biological evaluation, and in silico studies of 2-aminobenzothiazole derivatives as potent PI3Kα inhibitors. Arch. Pharm..

[52] Nawaz F, Alam O, Perwez A, Rizvi MA, Naim MJ, Siddiqui N, Pottoo FH, Jha M (2020). 3′-(4-(Benzyloxy)phenyl)-1′-phenyl-5-(heteroaryl/aryl)-3,4-dihydro-1′H,2H–3, bipyrazole -2-carboxamides as EGFR kinase inhibitors: Synthesis, anticancer evaluation, and molecular docking studies. Arch. Pharm..

[53] Haider K, Sharma S, Pokharel YR, Das S, Joseph A, Najmi AK, Ahmad F, Yar MS (2022). Synthesis, biological evaluation, and in silico studies of indole-tethered pyrazoline derivatives as anticancer agents targeting topoisomerase IIα. Drug Dev. Res..

[54] Mishra A, Mehdi SJ, Irshad M, Ali A, Sardar M (2012). Effect of biologically synthesized silver nanoparticles on human cancer cells. Sci. Adv. Mater..

[55] Banerjee PP, Bandyopadhyay A, Maloy PM, Mondal K, Chowdhury P, Chakraborty A, Sudarshan M, Bhattacharyam S, Chattopadhya A (2019). Cytotoxic effect of graphene oxide-functionalized gold nanoparticles in human breast cancer cell lines. Nculeus.

[56] Abu-Zaied MA, El-Telbani EM, Elgemeie GH, Nawwar GA (2011). Synthesis and in vitro anti-tumor activity of new oxadiazole thioglycosides. Eur. J. Med. Chem..

[57] Orphanos GS, Ioannidis GN, Ardavanis AG (2009). Cardiotoxicity induced by tyrosine kinase inhibitors. Acta Oncol..

[58] Mizushima Y, Kobayashi M (1968). Interaction of anti-inflammatory drugs with serum proteins, especially with some biologically active proteins. J. Pharm. Pharmacol..

[59] Winter CA, Risley EA, Nus GN (1962). Carrageenin-induced edema in hind paw of the rat as an assay for anti-inflammatory drugs. Exp. Biol..

[60] Cioli V, Putzolu S, Rossi V, Sorza B, Corradino P (1979). the role of direct tissue contacts in the production of gastrointestinal ulcers by anti-inflammatory drugs in rats. Toxicol. Appl. Pharm..

[61] Ohkawa H, Ohishi N, Yagi K (1979). Assay for lipid peroxides in animal tissues by thiobarbituric acid reaction. Anal. Biochem..

[62] Nath V, Paul KR, Kumar N, Kumar V (2022). Identification of behenic acid as medicinal food for the diabetes mellitus: Structure-based computational approach and molecular dynamics simulation studies. J. Mol. Model..

[63] Ugwu ID, Okoro UC, Ukoha PO, Gupta A (2018). Novel anti-inflammatory and analgesic agents: Synthesis, molecular docking and in vivo studies. J. Enzyme Inhibit. Med. Chem..

[64] Nath V, Ramchandani M, Kumar N, Agarwal R, Kumar V (2020). Computational identification of potential dipeptidyl peptidase (DPP)-IV) inhibitors: Structure based virtual screening, molecular dynamics. Simulation and knowledge-based SAR studies. J. Mol. Struct..

[65] Kumar KB, Faheem, Sekharb KVGC, Ojhac R, Kumar VP, Pai A, Murugesana S (2022). Pharmacophore based virtual screening, molecular docking, molecular dynamics and MM-GBSA approach for identification of prospective SARS-CoV-2 inhibitor from natural product databases. J. Biomol. Struct. Dyn..

