# Supplementary material for: Design, synthesis, biological assessment and molecular modeling studies of novel imidazothiazole-thiazolidinone hybrids as potential anticancer and anti-inflammatory agents
Source: Sci Rep. 2024 Apr 11;14:8457. doi: 10.1038/s41598-024-59063-x (PMC11009276; doi:10.1038/s41598-024-59063-x)
Supplement: Supplementary file 1 — Supplementary Information. [file 41598_2024_59063_MOESM1_ESM.pdf]

Supplemental Material

Design, synthesis, biological assessment and molecular modeling studies of novel imidazothiazole-thiazolidinone hybrids as potential anticancer and anti-inflammatory agents

Payal Kamboj,[1] Anjali,[1] Khalid Imtiyaz[2], Moshahid A. Rizvi,[2] Virendra Nath,[3] Vipin Kumar[3], Asif husain[1], Mohd. Amir[1]

[1]Department of Pharmaceutical Chemistry, School of Pharmaceutical Education & Research, Jamia Hamdard, India

[2]Genome Biology Lab, Department of Biosciences, Jamia Millia Islamia, New Delhi, India

[3]Department of Pharmacy, Central University of Rajasthan, Ajmer

***Corresponding author –** Prof. Mohd. Amir, Department of Pharmaceutical Chemistry, School of Pharmaceutical Education and Research, Jamia Hamdard, New Delhi-110062, Email: mamir@jamiahamdard.ac.in

Table of content

1. General procedure for the synthesis of carbaldehyde **1-2**
2. General procedure for the synthesis of N-(substituted phenyl)-1-(6-phenylimidazo [2, 1-b] thiazol-5-yl) methanimine (3a-g) **2-3**
3. Supplementary figures ^{1}H-NMR, ^{13}C-NMR, HRMS spectrum of the compounds (S1-S32) **3-23**

General procedure for the synthesis of 6-phenylimidazo [2, 1-b] thiazole-5-carbaldehyde (2)

Compound **1** (0.03 mol) was dissolved in $CHCl_3$ (25 ml) and DMF (0.06 mol) in a round bottom flask with a magnetic stirrer and $POCl_3$ (0.06 mol) was added to it drop wise with continuous stirring at 0-5°C for 30 min and then the reaction mixture was refluxed for 3-4 hr. Completion of reaction was monitored by TLC in ethyl acetate/petroleum ether (8:2) solvent system. Upon completion, the solvent was removed. Under reduced pressure. The oily product thus obtained was washed with water and extracted with dichloromethane (DCM). Upon removal of DCM under reduced pressure solid product was obtained which was dried and recrystalize from ethanol.[52]

Yield: 80%, m.p. 147^{0}C; ^{1}H NMR (400 MHz, $CDCl_3$) δ (ppm): 7.08 (1H, d, J=4.4 CH) 7.51-7.55 (3H, m, Ar-H),7.81-7.83(2H, t, Ar-H), 8.41 (1H, d, J=4.4, CH), 9.92 (s, 1H, CHO).

General procedure for the synthesis of N-(substituted phenyl)-1-(6-phenylimidazo [2, 1-b] thiazol-5-yl) methanimine (3a-g)

6-Phenylimidazo [2, 1-b] thiazole-5-carbaldehyde **2** (0.001mol) was dissolved in toluene (25 ml) in a round bottom flask and substituted anilines (0.001mol) was added to it. The solution was thoroughly mixed and *p*-toluene sulfonic acid (0 .0001mol) was added as a catalyst. The reaction mixture was refluxed for 5-8 hr. The progress of the reaction was monitored by TLC. Upon completion, the solvent was removed by rotavapor, the solid thus obtained was washed with diethyl ether, air dried and recrystalized with ethanol.

N-(4-Fluorophenyl)-1-(6-phenylimidazo [2, 1-b] thiazol-5-yl) methanimine (3a)

Yield: 67%, m.p. 153-155^{0}C; ^{1}H NMR (400 MHz, $CDCl_3$) δ (ppm): 6.88 (1H, d, J=4.4 Hz, CH), 7.28-7.82 (5H, m, Ar-H), 7.30 (2H, d, J=7.5 Hz, Ar-H), 8.40 (2H, d, J=7.5 Hz, Ar-H), 8.53 (1H, d, J=4.4 CH), 8.62 (1H, s, CH=N).

N-(4-Chlorophenyl)-1-(6-phenylimidazo [2, 1-b] thiazol-5-yl) methanimine (3b)

Yield: 75%, m.p. 187-189^{0}C; ^{1}H NMR (400 MHz, $CDCl_3$) δ (ppm): 6.85 (1H, d, J=4.4 Hz, CH), 7.40-759 (5H, m, Ar-H), 7.23 (2H, d, J=7.5 Hz), 8.16 (2H, d, J=7.5 Hz), 8.21 (1H, d, J=4.4 Hz, CH), 8.36 (1H, s, CH=N).

N-(4-Bromophenyl)-1-(6-phenylimidazo [2, 1-b] thiazol-5-yl) methanimine (3c)

Yield: 71%, m.p. 168-170^{0}C; ^{1}H NMR (400 MHz, $CDCl_3$) δ (ppm): 6.81 (1H, d, J=4.4 Hz, CH), 7.28-7.82 (5H, m, Ar-H), 7.19 (2H, d, J=7.5 Hz, Ar-H), 8.13 (2H, d, J=7.5 Hz, Ar-H), 8.17 (1H, d, J=4.4 CH), 8.29 (1H, s, CH=N).

N-(2,4-Dichlorophenyl)-1-(6-phenylimidazo[2,1-b] thiazol-5-yl) methanimine (3d)

Yield 64%, m.p. 150-152^{0}C; ^{1}H NMR (400 MHz, $CDCl_3$): δ (ppm): 6.99 (1H, d, J=4.5 Hz, CH), 7.03 (1H, dd, J=7.5 Hz, 2.5 Hz, Ar-H), 7.29 (1H, d, J=7.5 Hz, Ar-H), 7.42-7.52 (5H, m, Ar-H), 7.73 (1H, d, J=2.5 Hz, Ar-H), 8.24 (1H, d, J=4.5Hz, CH), 8.41 (s,1H, CH=N).

N-(4-Nitrophenyl)-1-(6-phenylimidazo [2, 1-b] thiazol-5-yl) methanimine (3e)

Yield 77%, m.p. 167-169^{0}C; ^{1}H NMR (400 MHz, $CDCl_3$) δ (ppm): 7.05 (1H, d, J= 4.5 Hz, CH), 7.28- 7.54 (5H, m, Ar-H), 7.78 (2H, d, J=7 Hz, Ar-H), 8.28 (2H, d, J=7 Hz, Ar-H), 8.64 (1H, d, J=4.5 Hz, CH), 8.62 (1H, s, CH=N).

1-(6-Phenylimidazo [2, 1-b] thiazol-5-yl)-N-(*p*-tolyl) methanimine (3f)

Yield 73%, m.p. 170-174^{0}C; ^{1}H NMR (400 MHz, $CDCl_3$) δ (ppm): 2.30 (3H, s, CH_3), 6.95 (1H, d, J= 4.5Hz, CH), 6.98- 7.24 (5H, m, Ar-H), 7.48 (2H, d, J=7 Hz, Ar-H), 7.96 (2H, d, J=7 Hz, Ar-H), 8.17 (1H, d, J=4.5 Hz, CH), 8.58 (1H, s, CH=N).

N-(4-Methoxyphenyl)-1-(6-phenylimidazo [2, 1-b] thiazol-5-yl) methanimine (3g)

Yield = 72%; m.p. 172-174^{0}C; ^{1}H NMR (400 MHz, $CDCl_3$) δ (ppm): 3.84 (s, 3H, OCH_3), 6.93 (1H, d, J= 4.5 Hz, CH), 6.96-7.19 (5H m, Ar-H), 7.20 (2H, d, J=7 Hz, Ar-H), 7.41 (2H, d, J=7 Hz, Ar-H), 7.58 (1H, d, J= 4.5 Hz, CH), 8.66 (1H, s, CH=N).

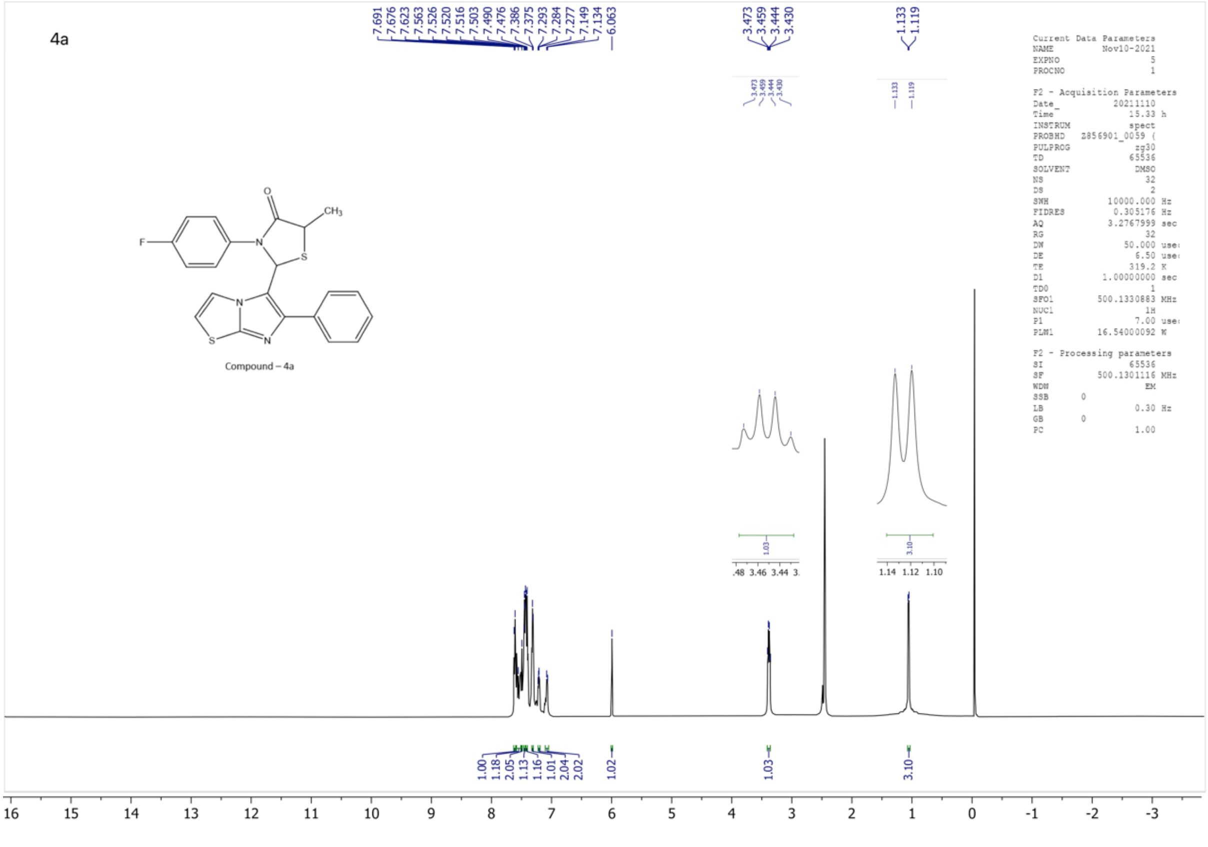

Figure S1. ^{1}H NMR spectra of compound **4a**

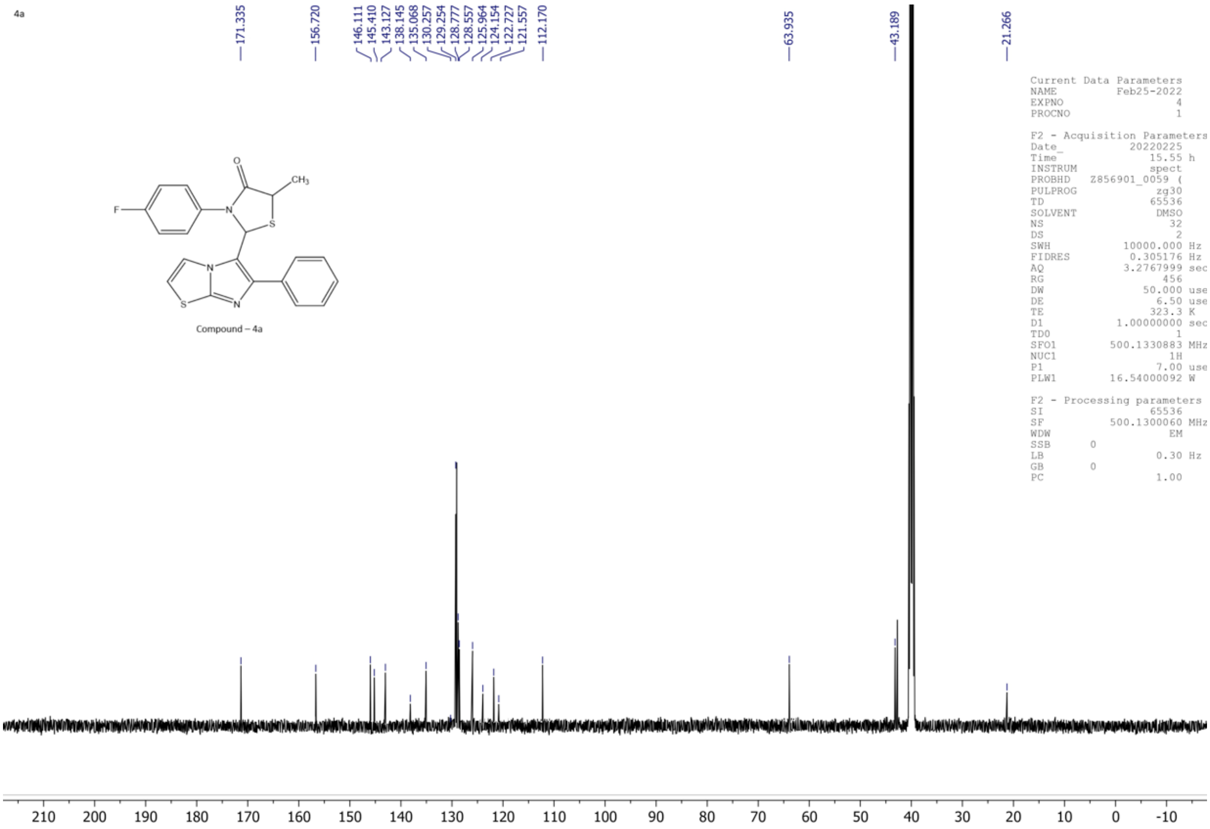

Figure S2. ^{13}C NMR spectra of compound **4a**

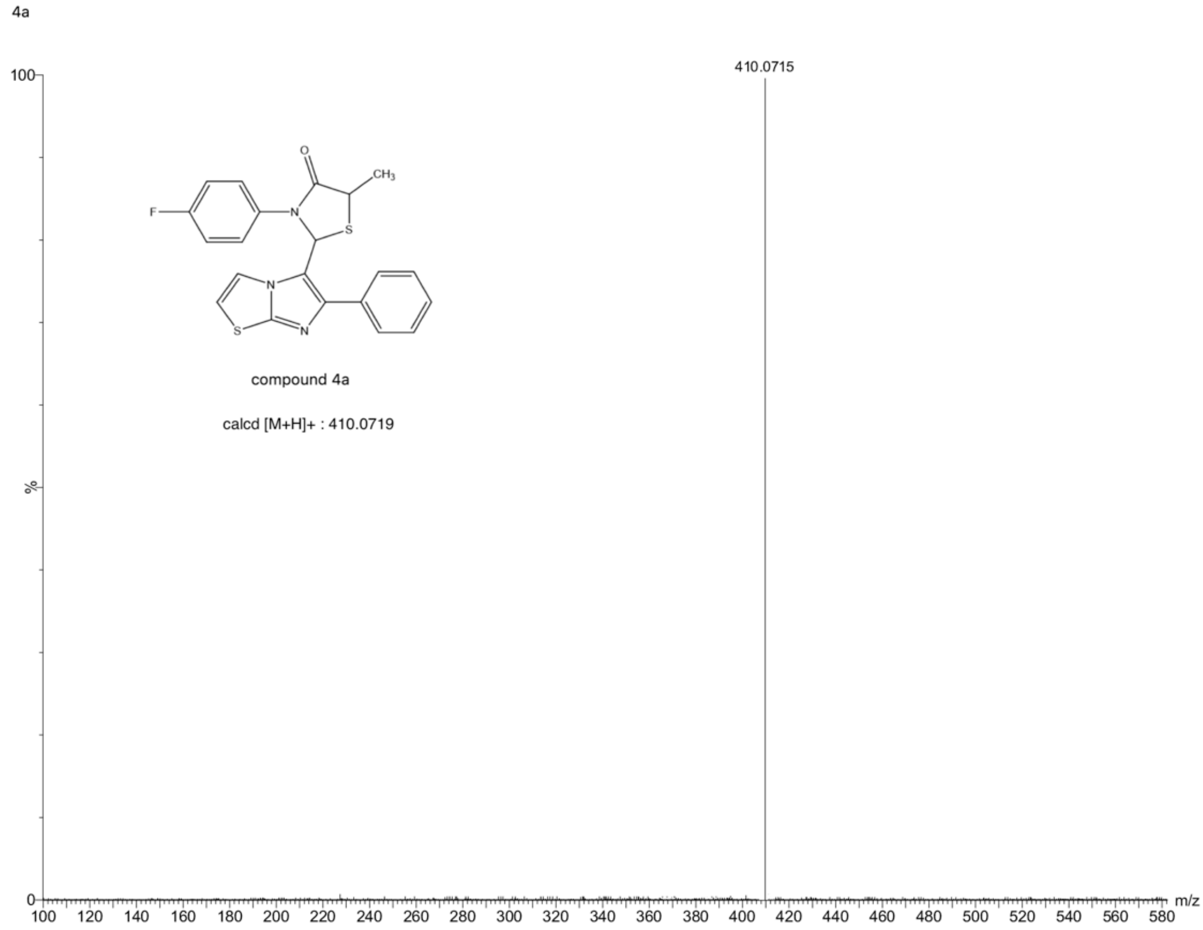

Figure S3. HRMS spectra of compound **4a**

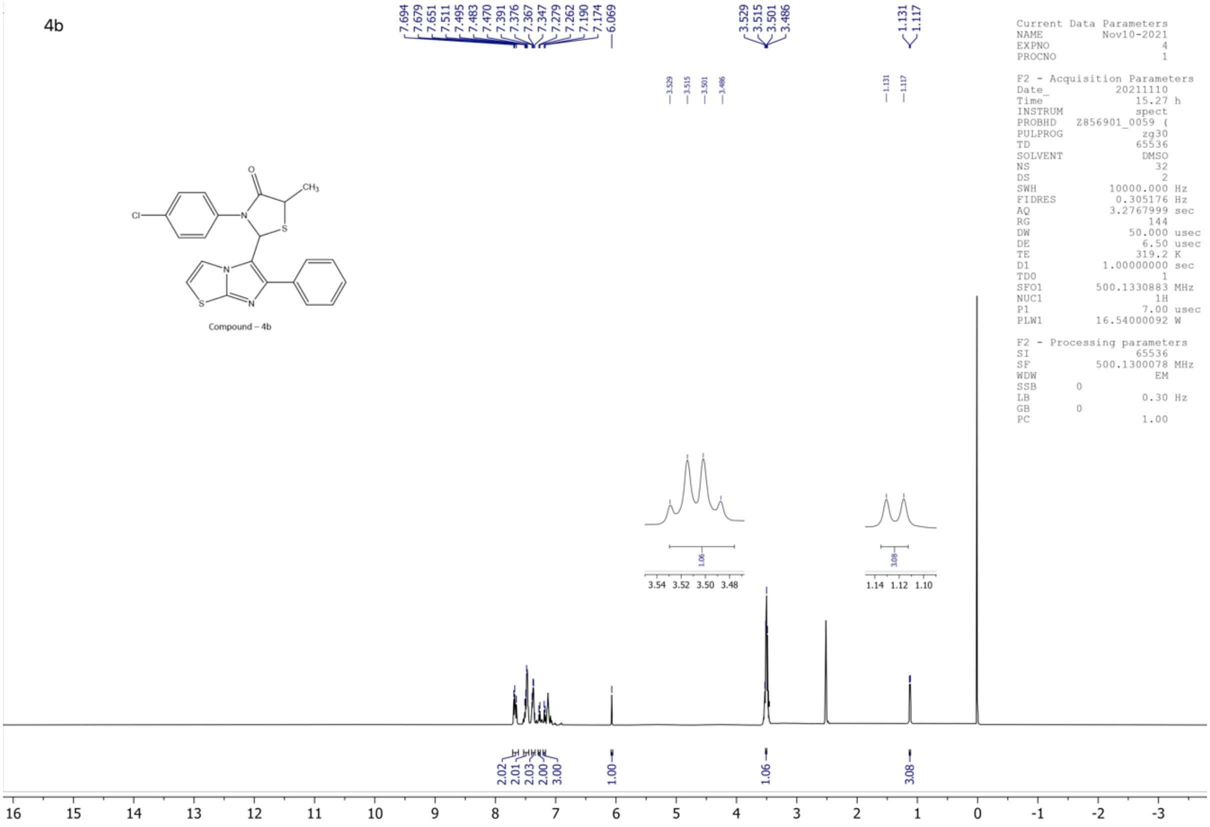

Figure S4. ^{1}H NMR spectra of compound **4b**

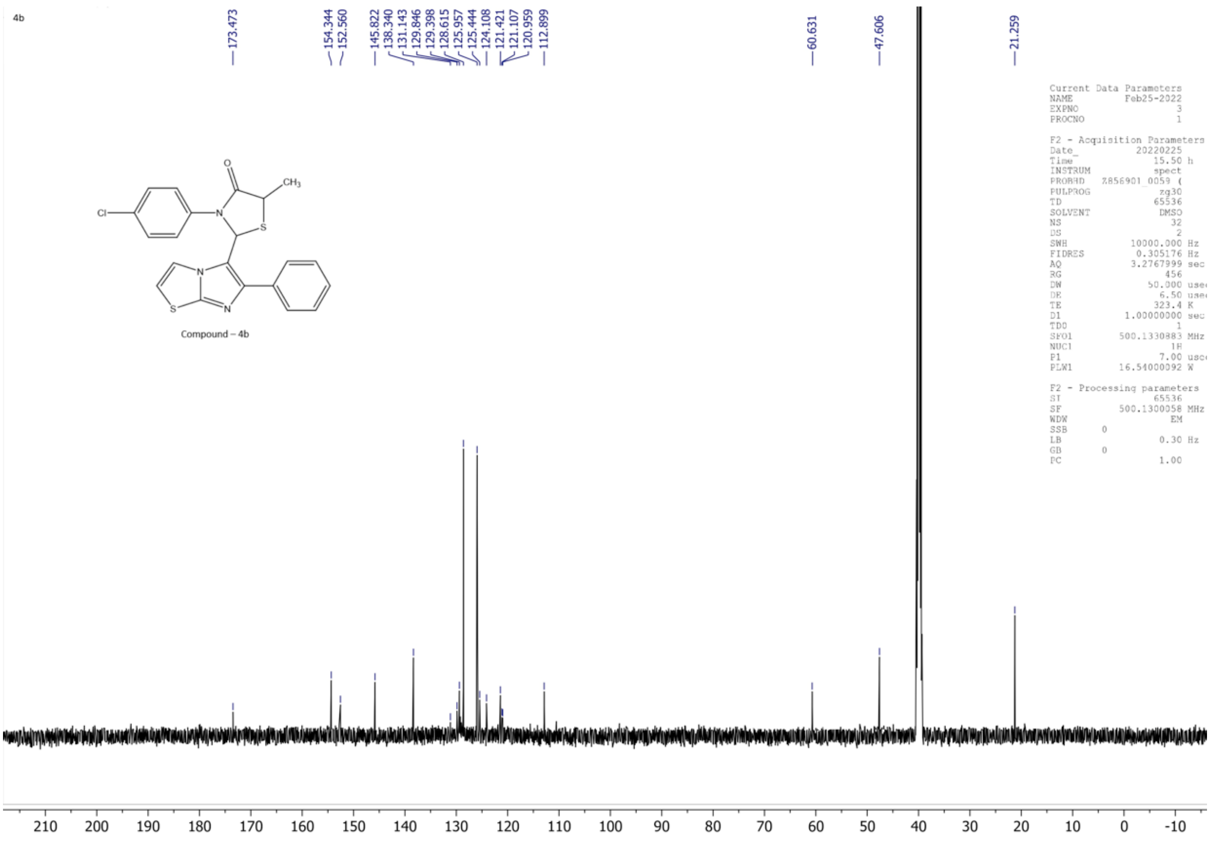

Figure S5. ^{13}C NMR spectra of compound **4b**

4b

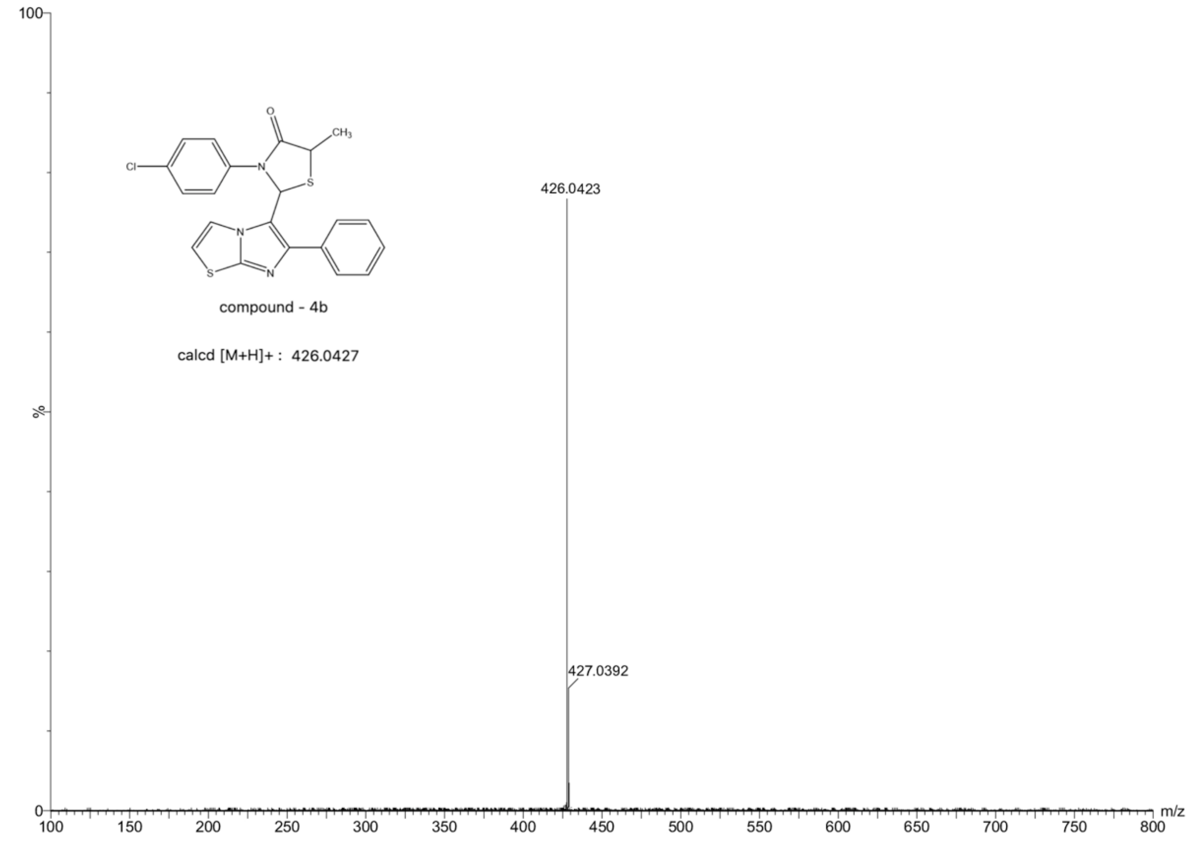

Figure S6. HRMS spectra of compound **4b**

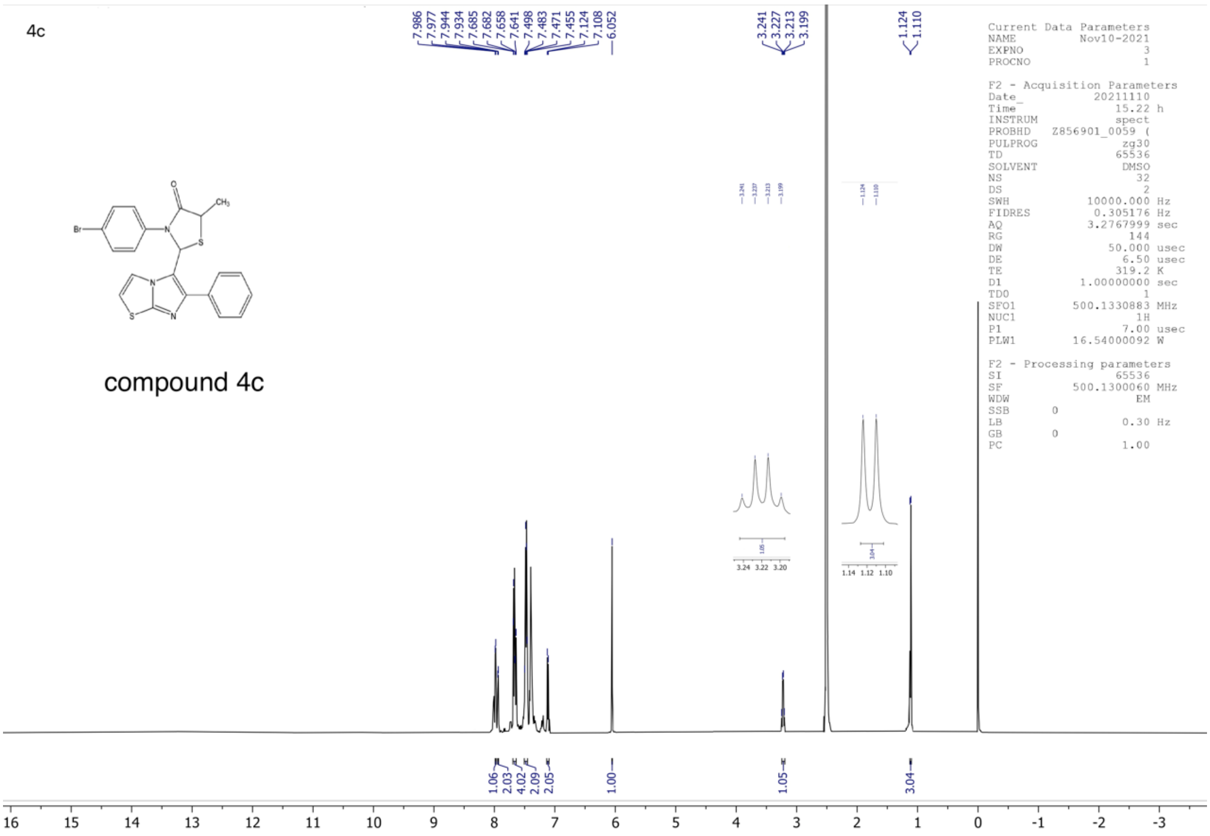

Figure S7. ^{1}H NMR spectra of compound **4c**

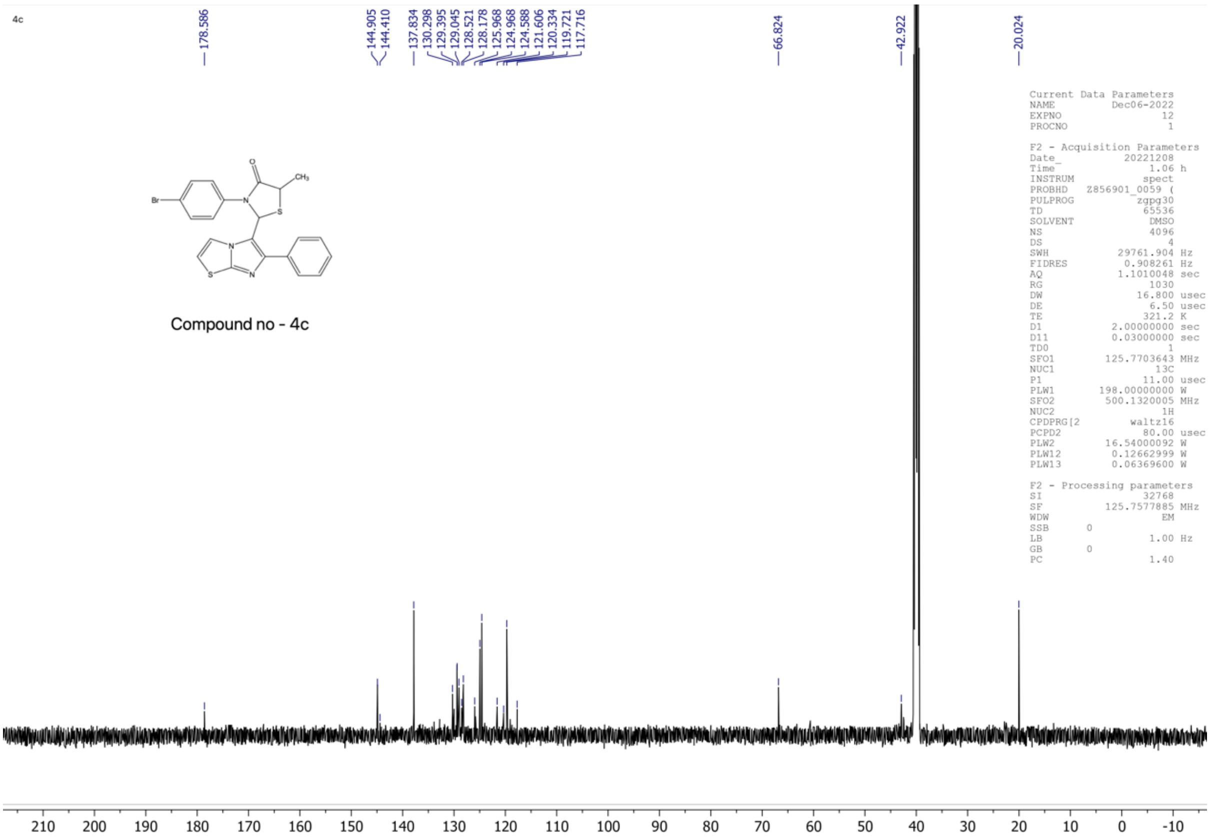

Figure S8. ^{13}C NMR spectra of compound **4c**

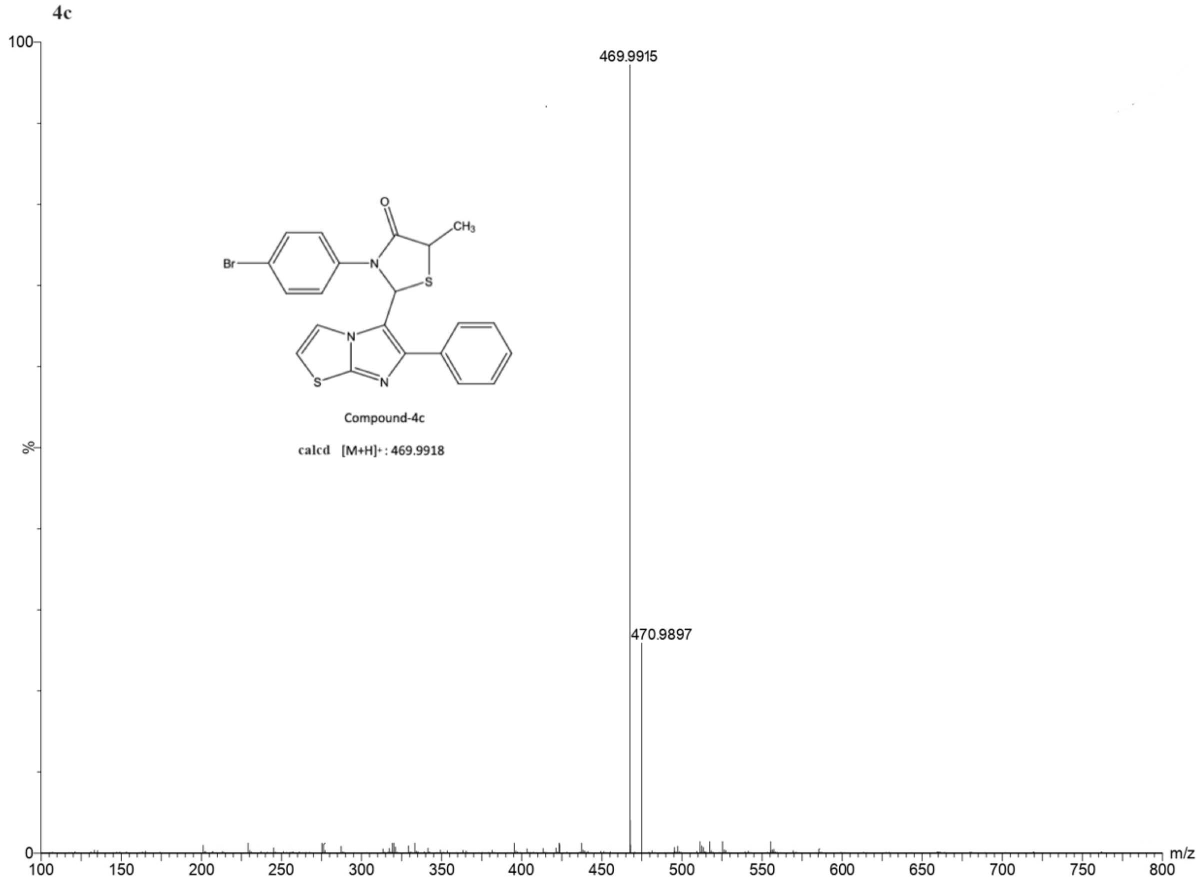

Figure S9. HRMS spectra of compound **4c**

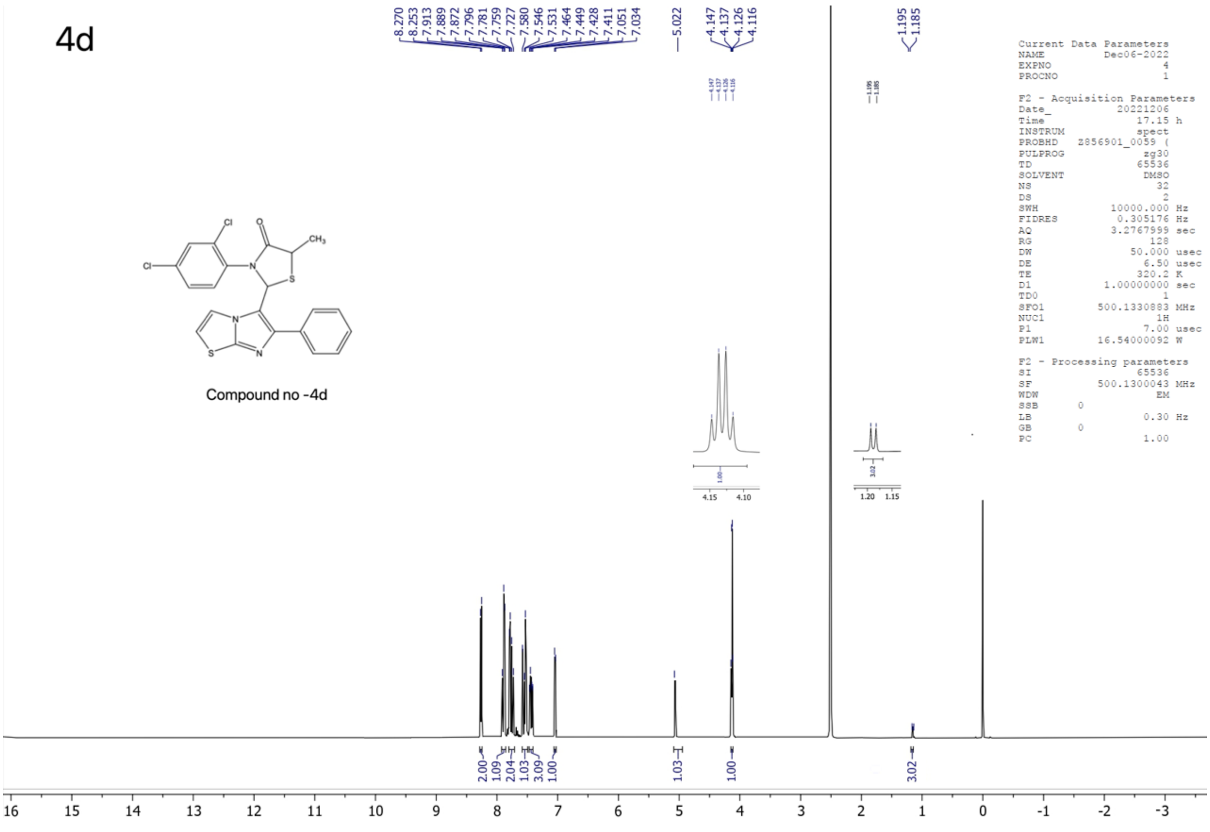

Figure S10. ^{1}H NMR spectra of compound **4d**

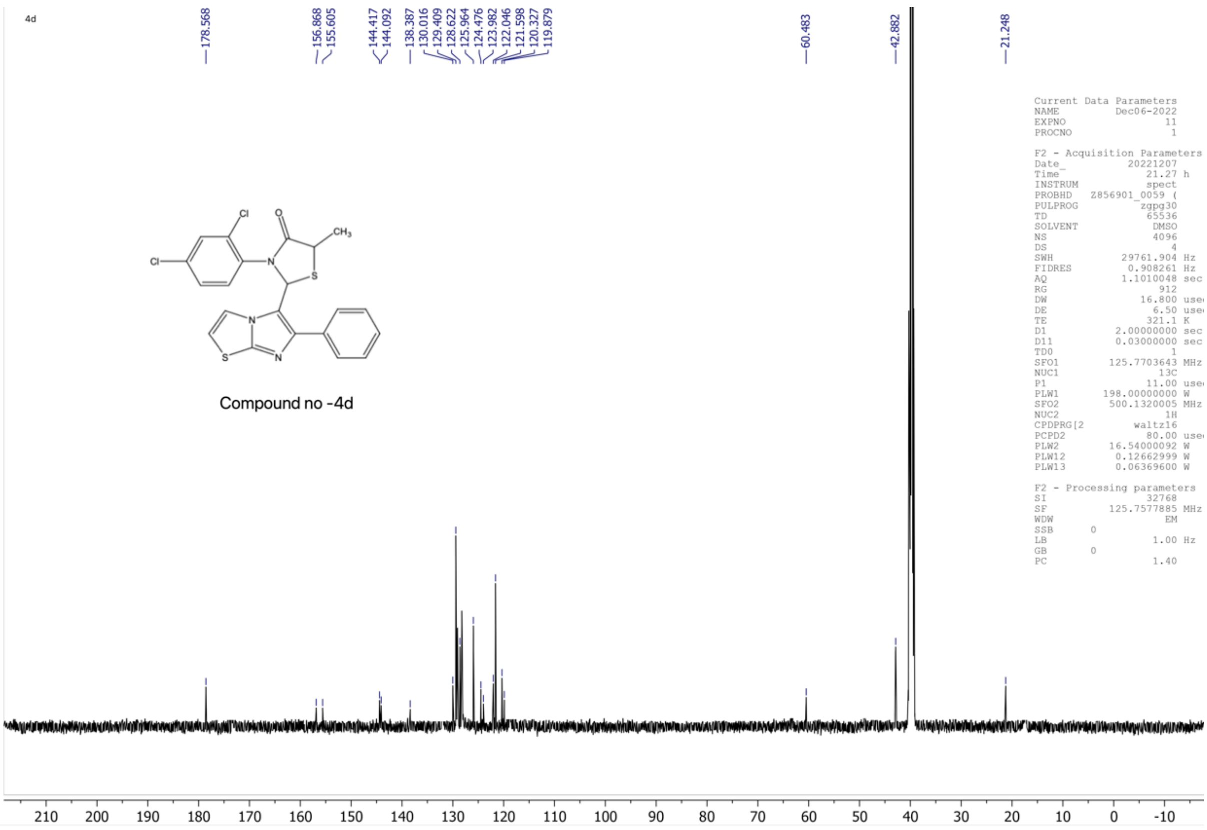

Figure S11. ^{13}C NMR spectra of compound **4d**

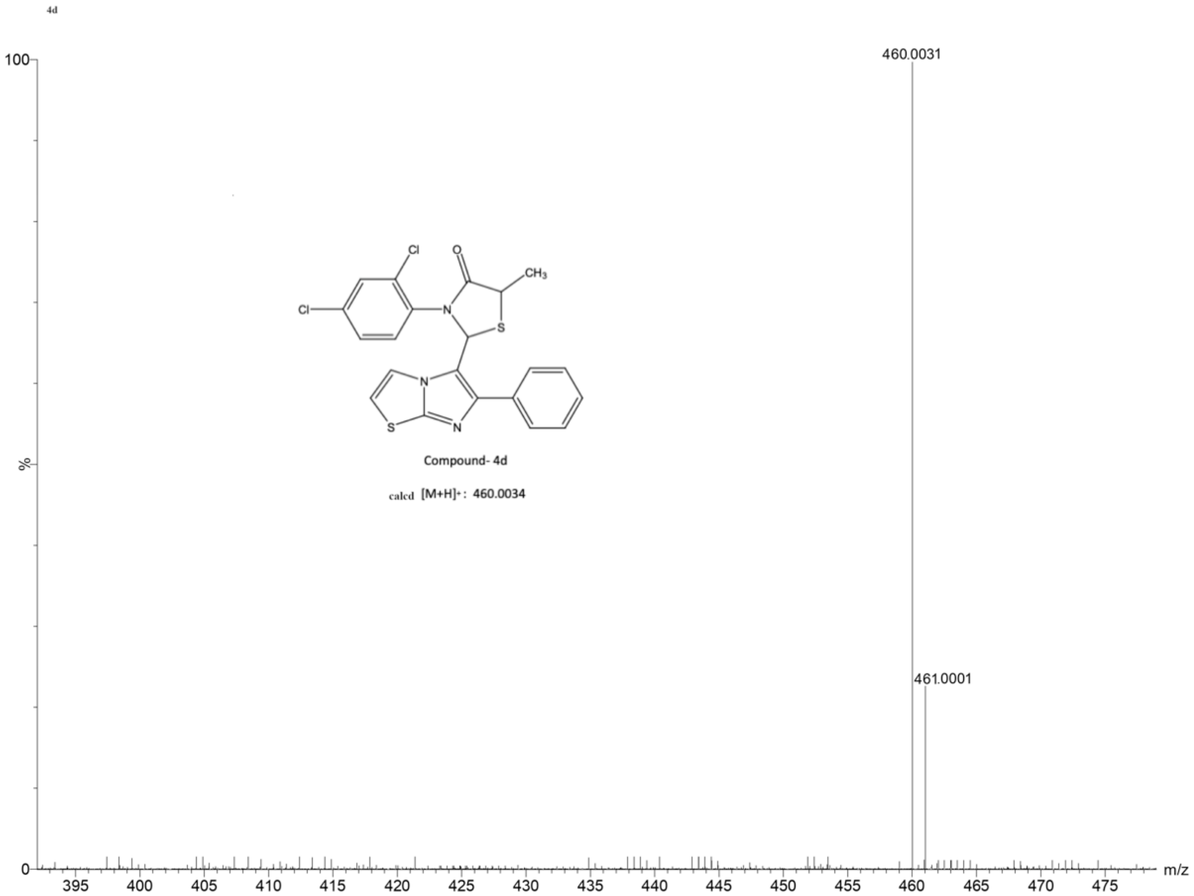

Figure S12. HRMS spectra of compound **4d**

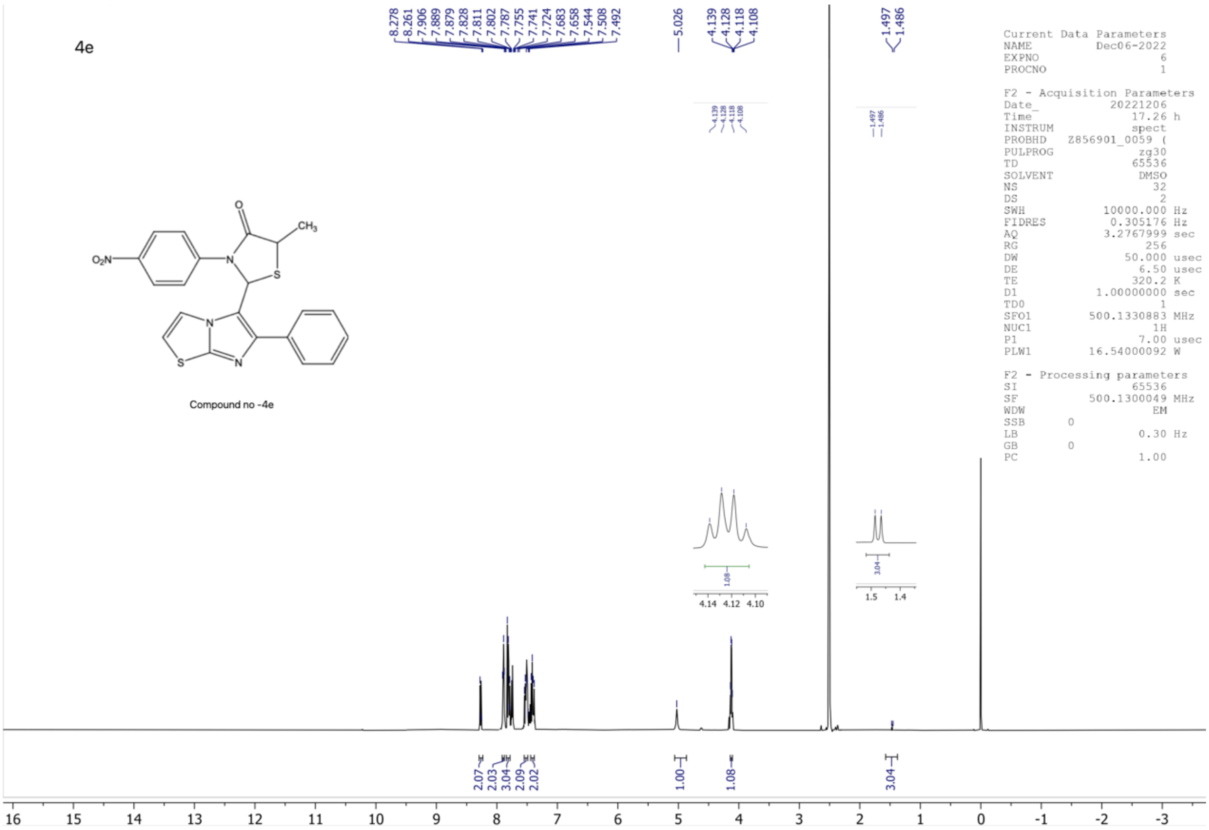

Figure S13. ^{1}H NMR spectra of compound **4e**

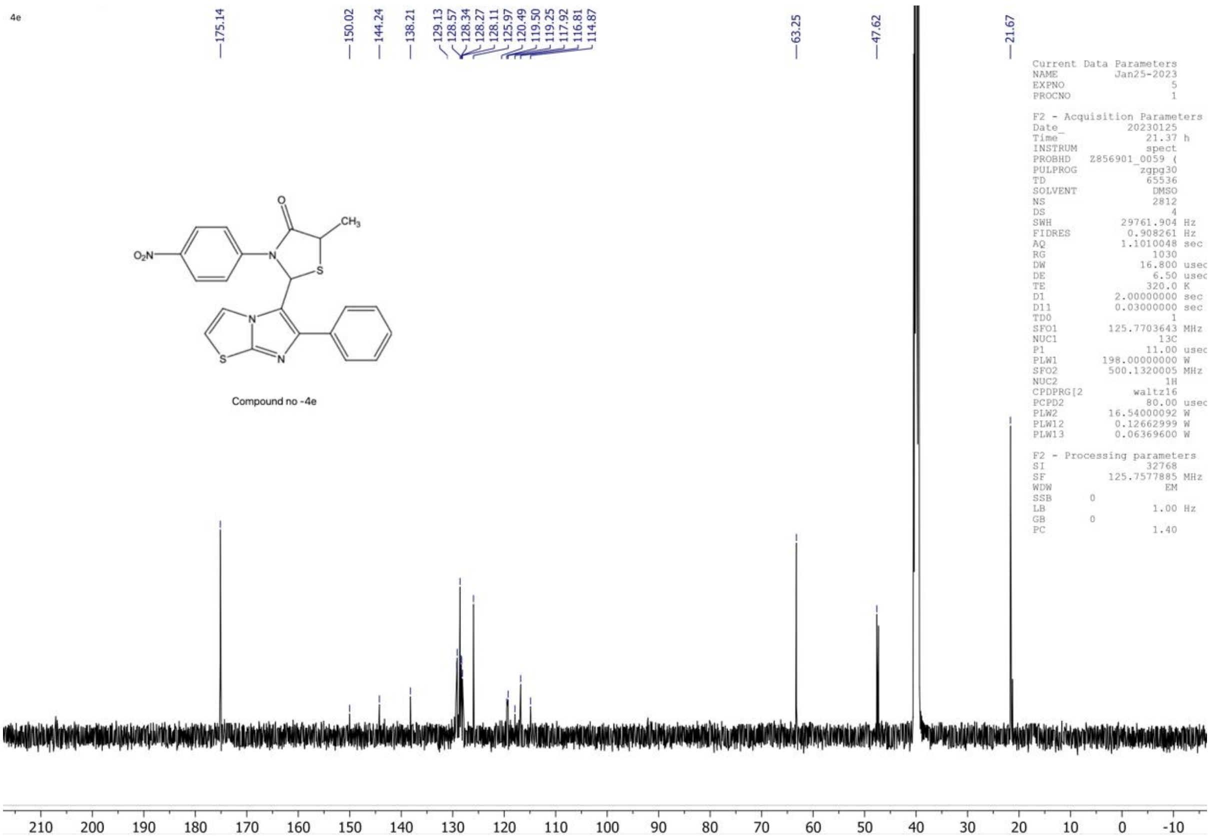

Figure S14. ^{13}C NMR spectra of compound **4e**

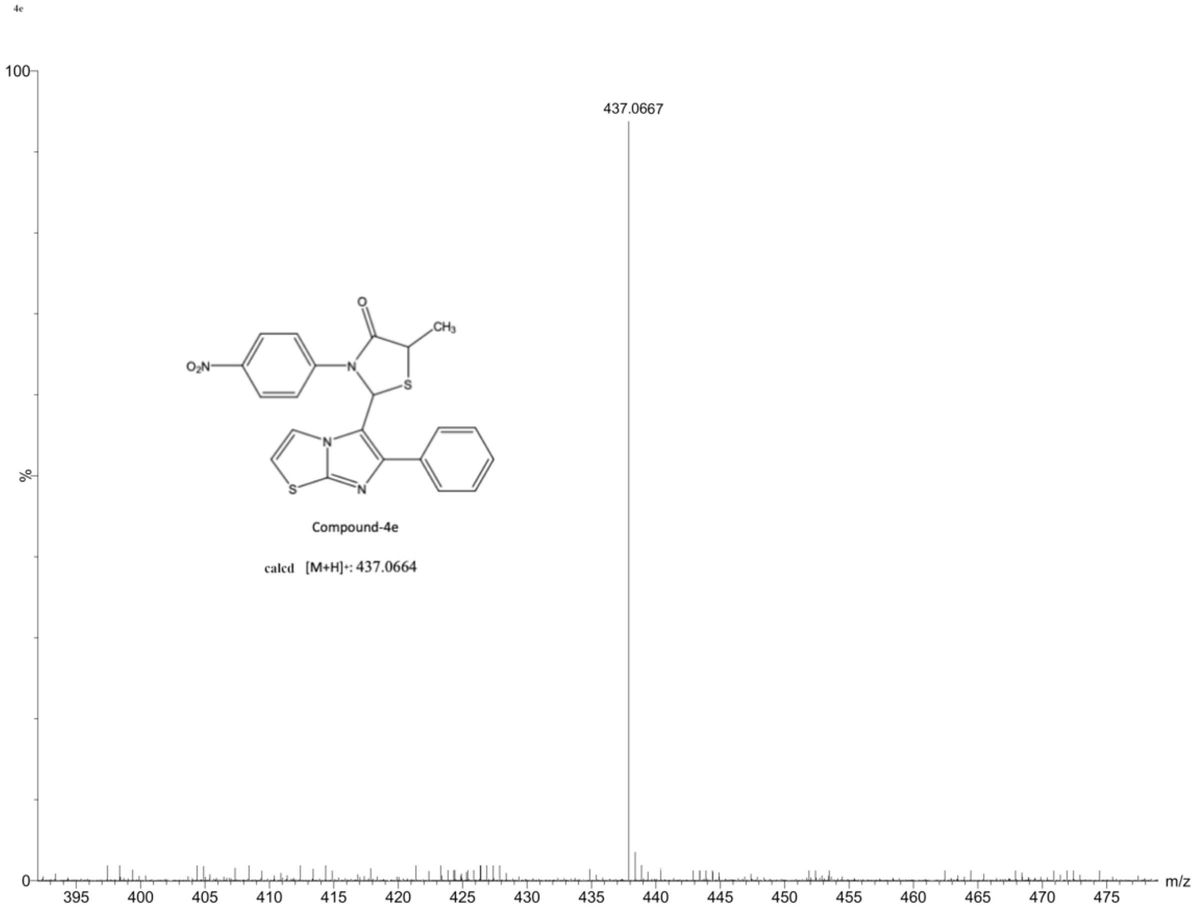

Figure S15. HRMS spectra of compound **4e**

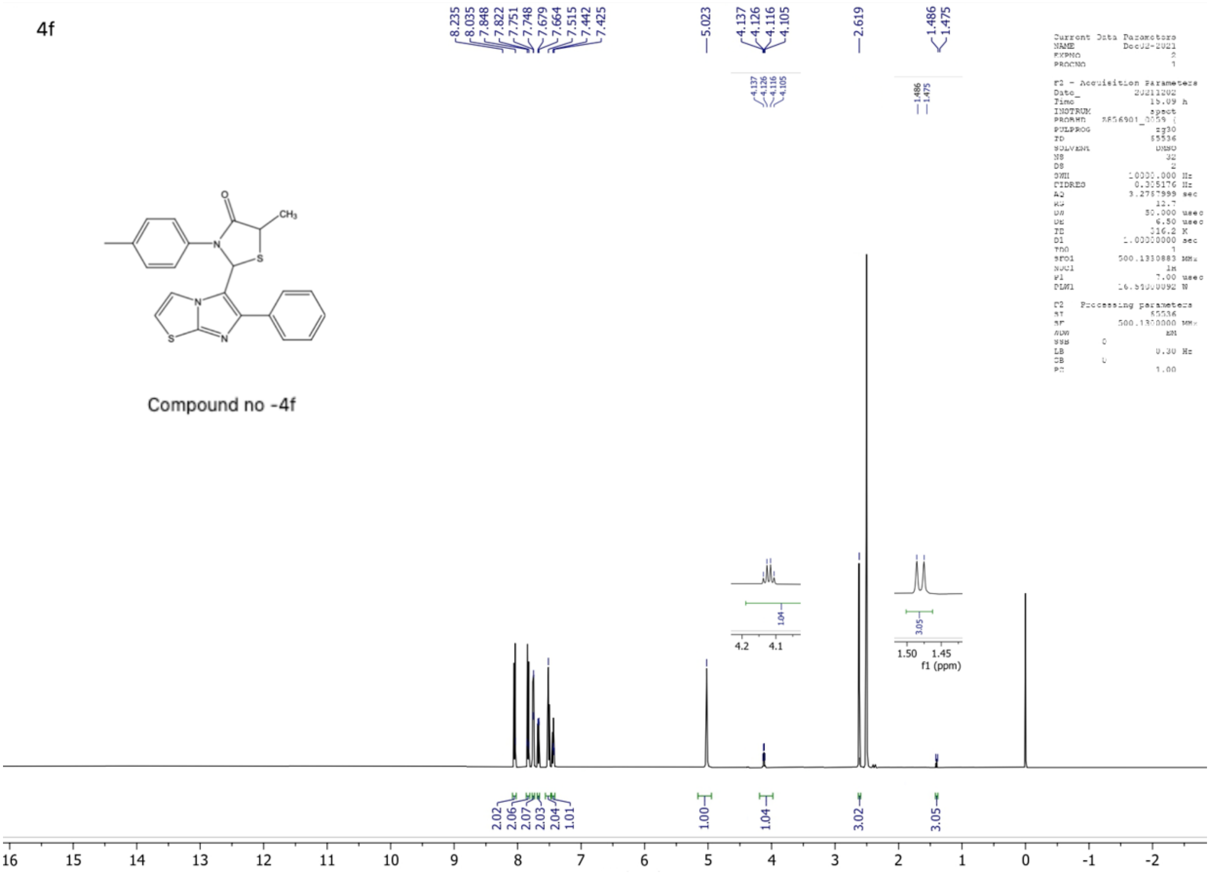

Figure S16. ^{1}H NMR spectra of compound **4f**

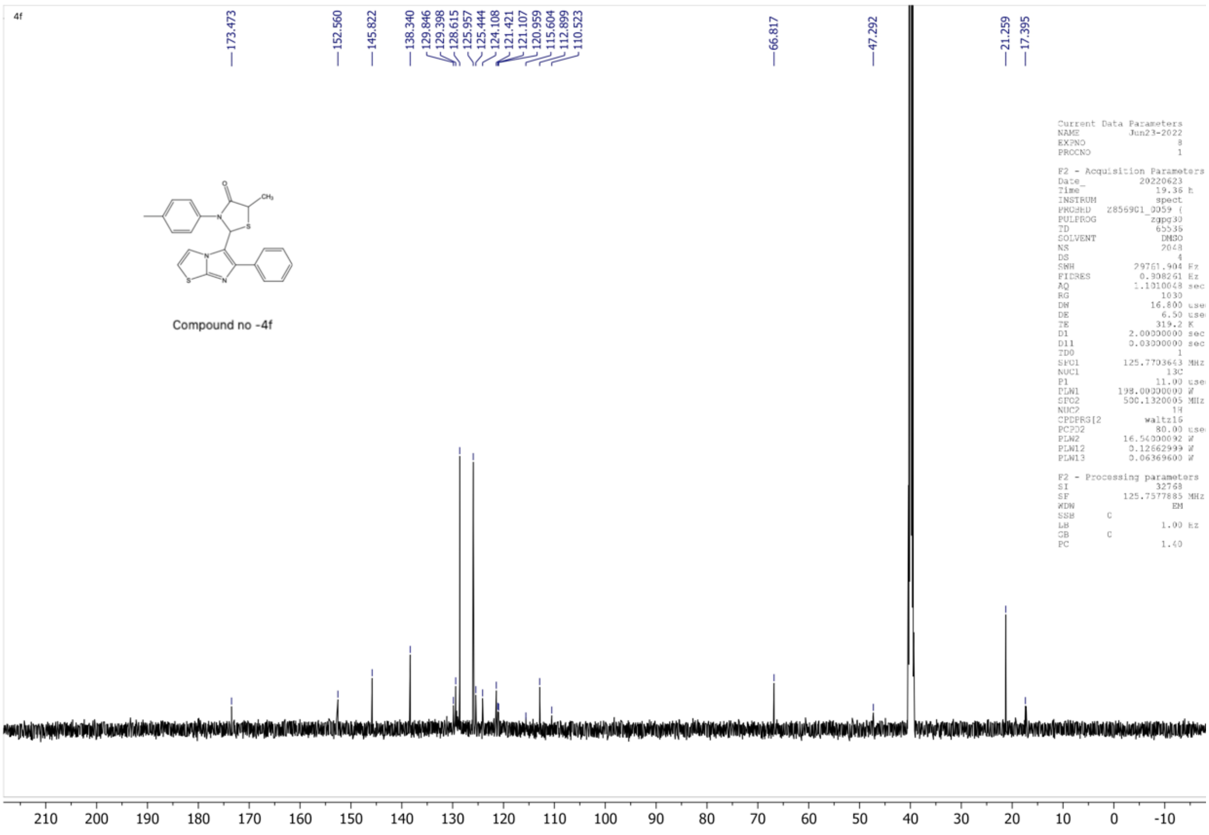

Figure S17. ^{13}C NMR spectra of compound **4f**

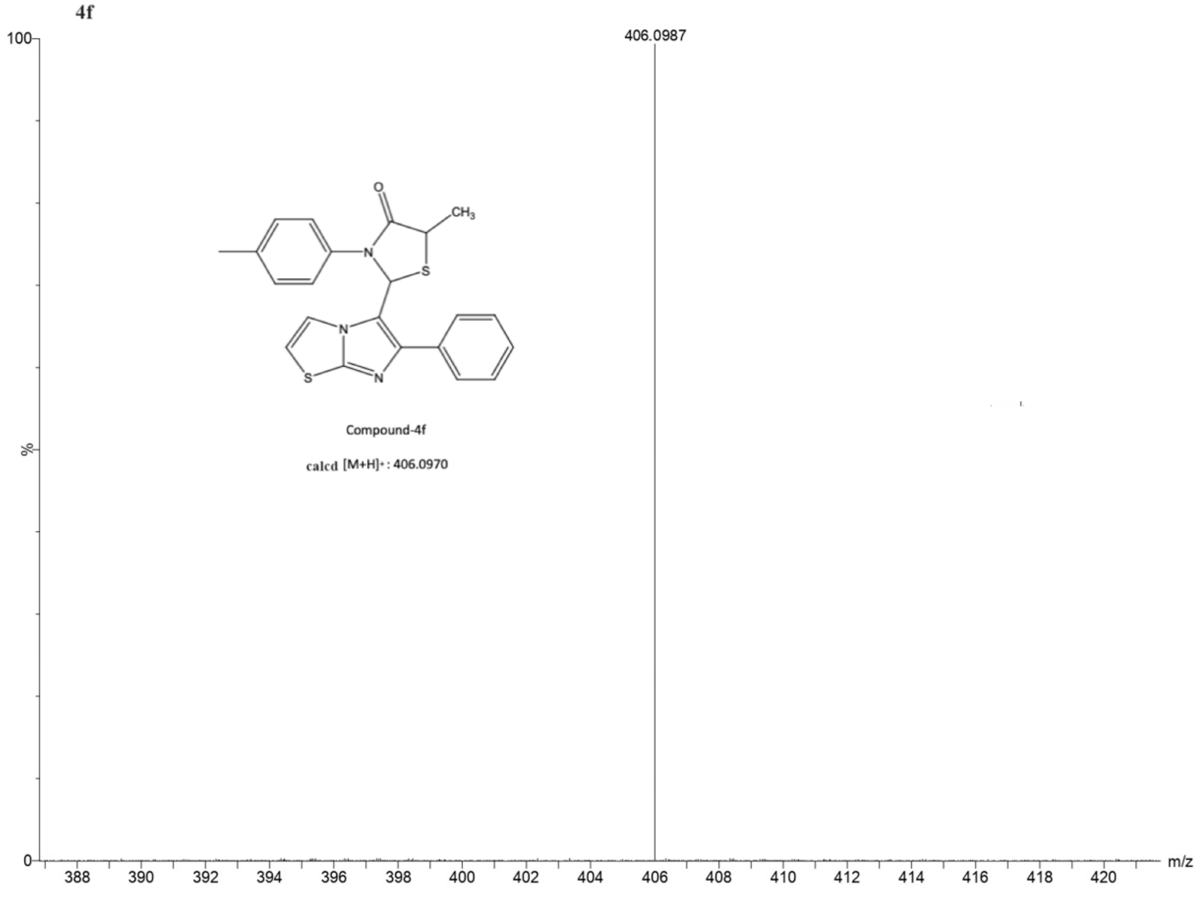

Figure S18. HRMS spectra of compound **4f**

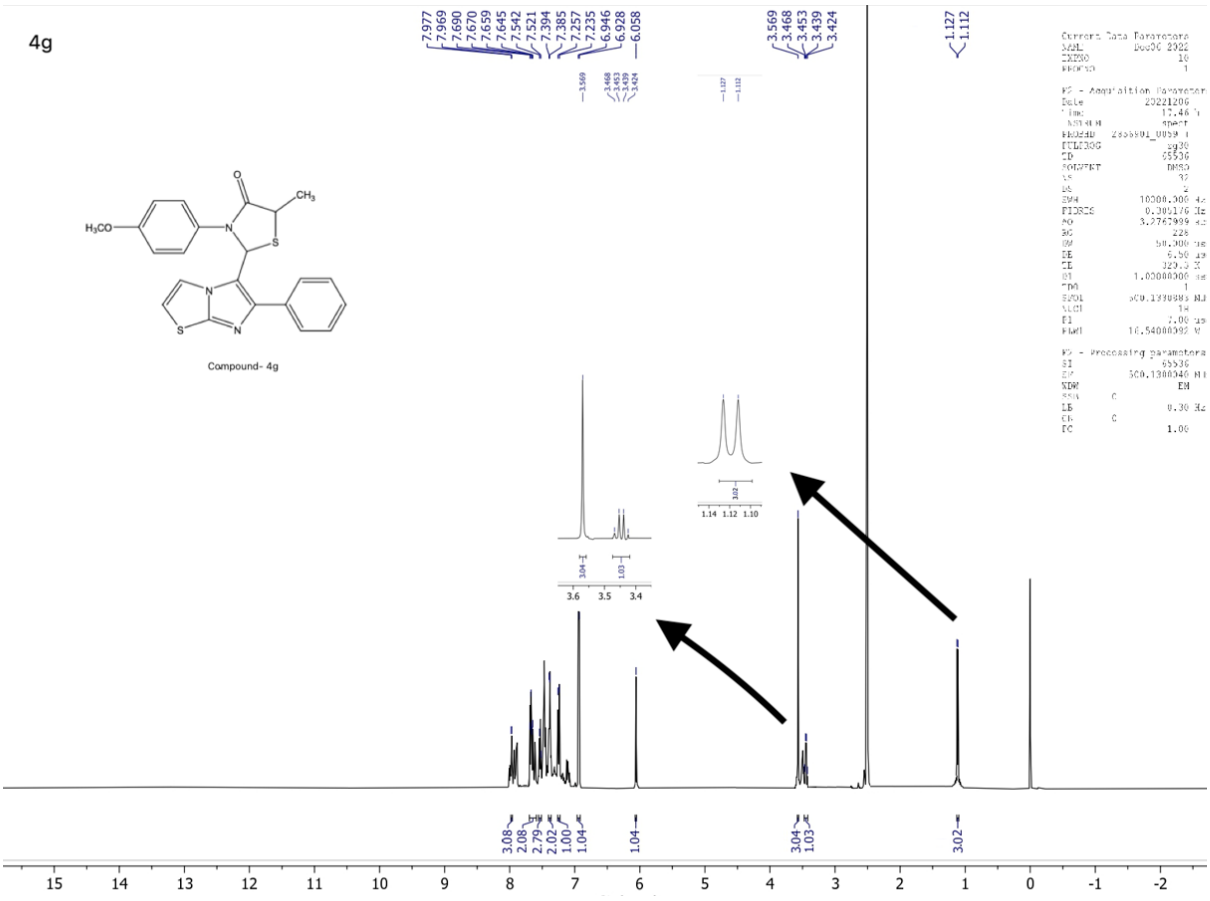

Figure S19. ^{1}H NMR spectra of compound **4g**

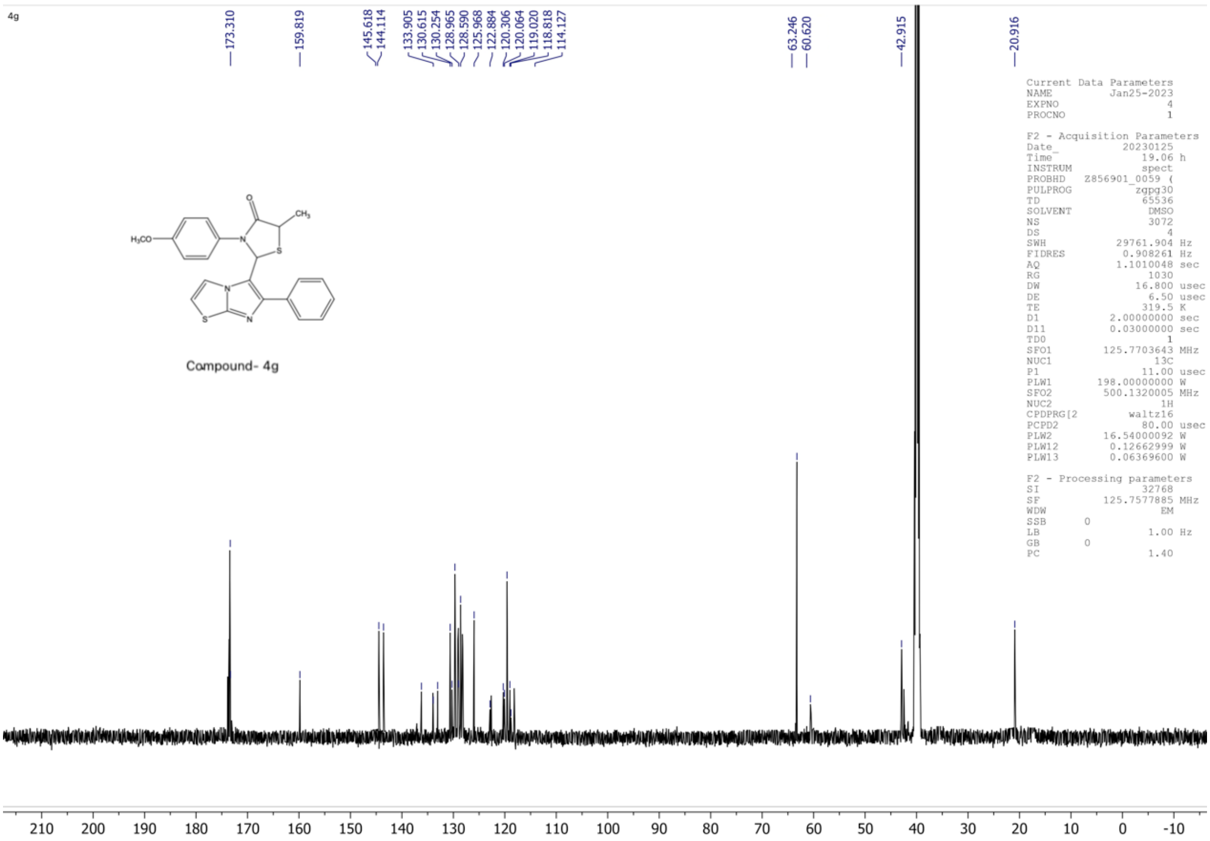

Figure S20. ^{13}C NMR spectra of compound **4g**

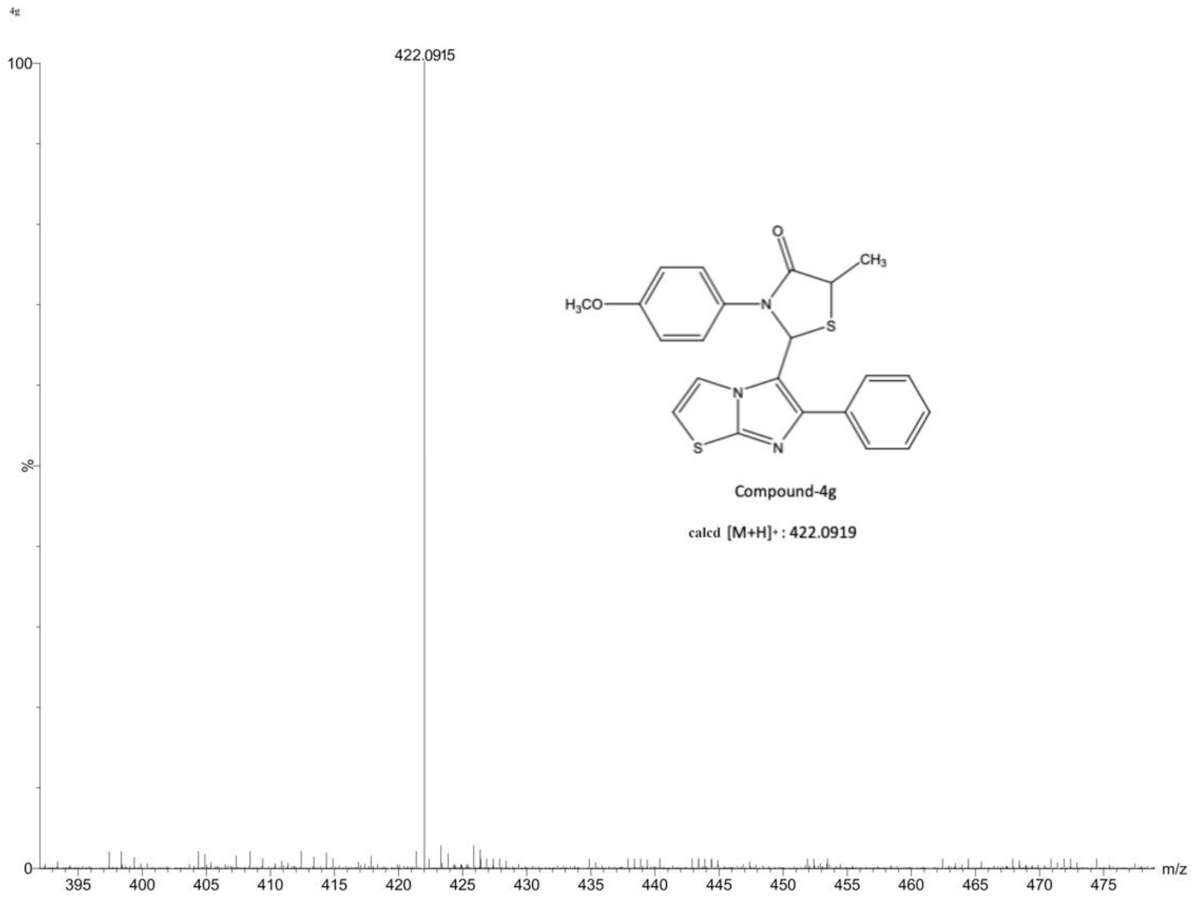

Figure S21. HRMS spectra of compound **4g**

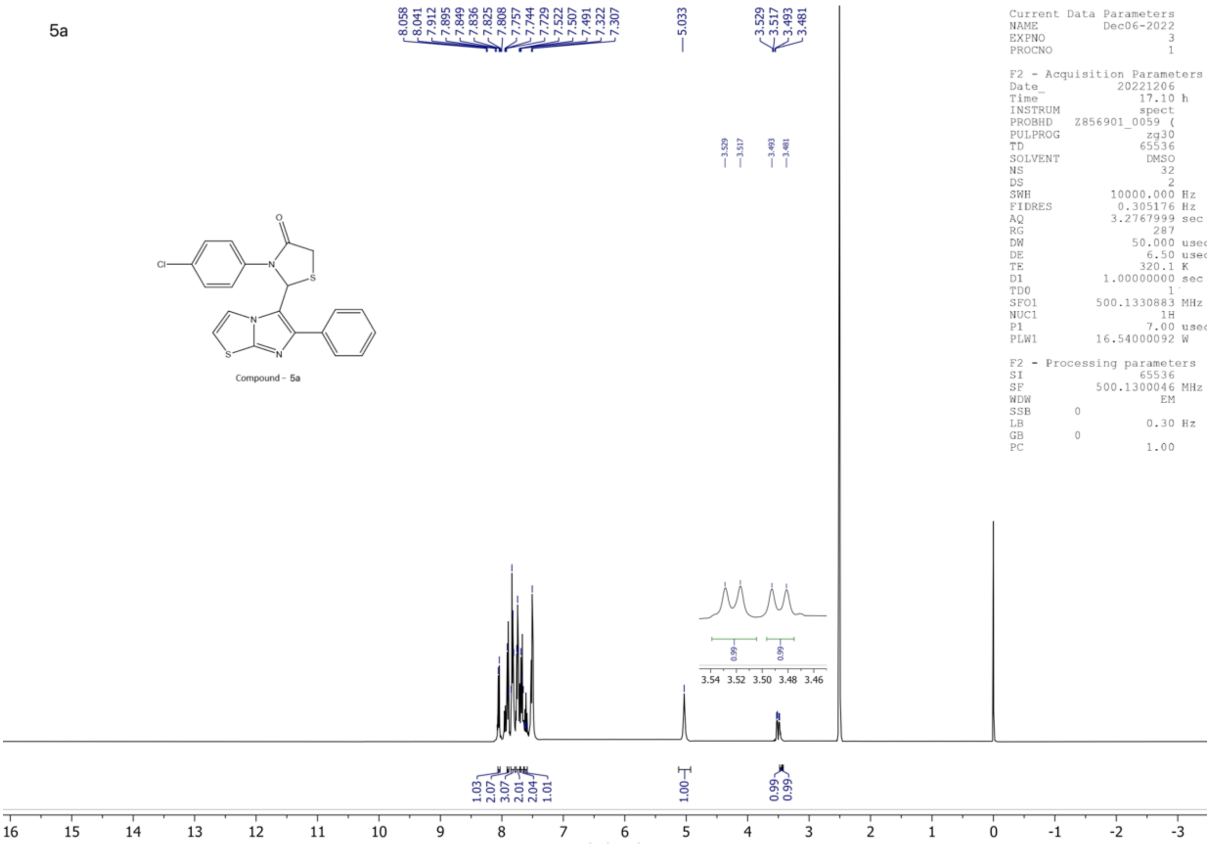

Figure S22. ^{1}H NMR spectra of compound **5a**

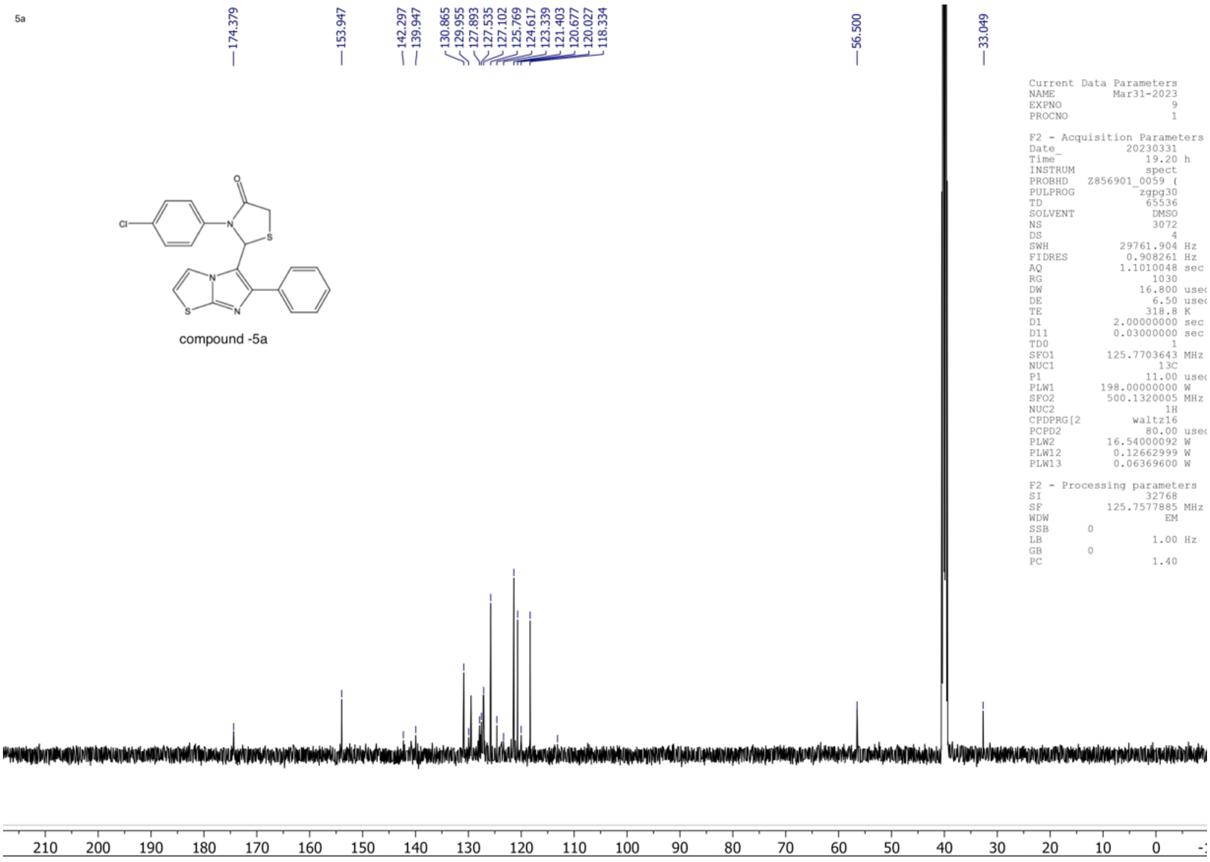

Figure S23. ^{13}C NMR spectra of compound **5a**

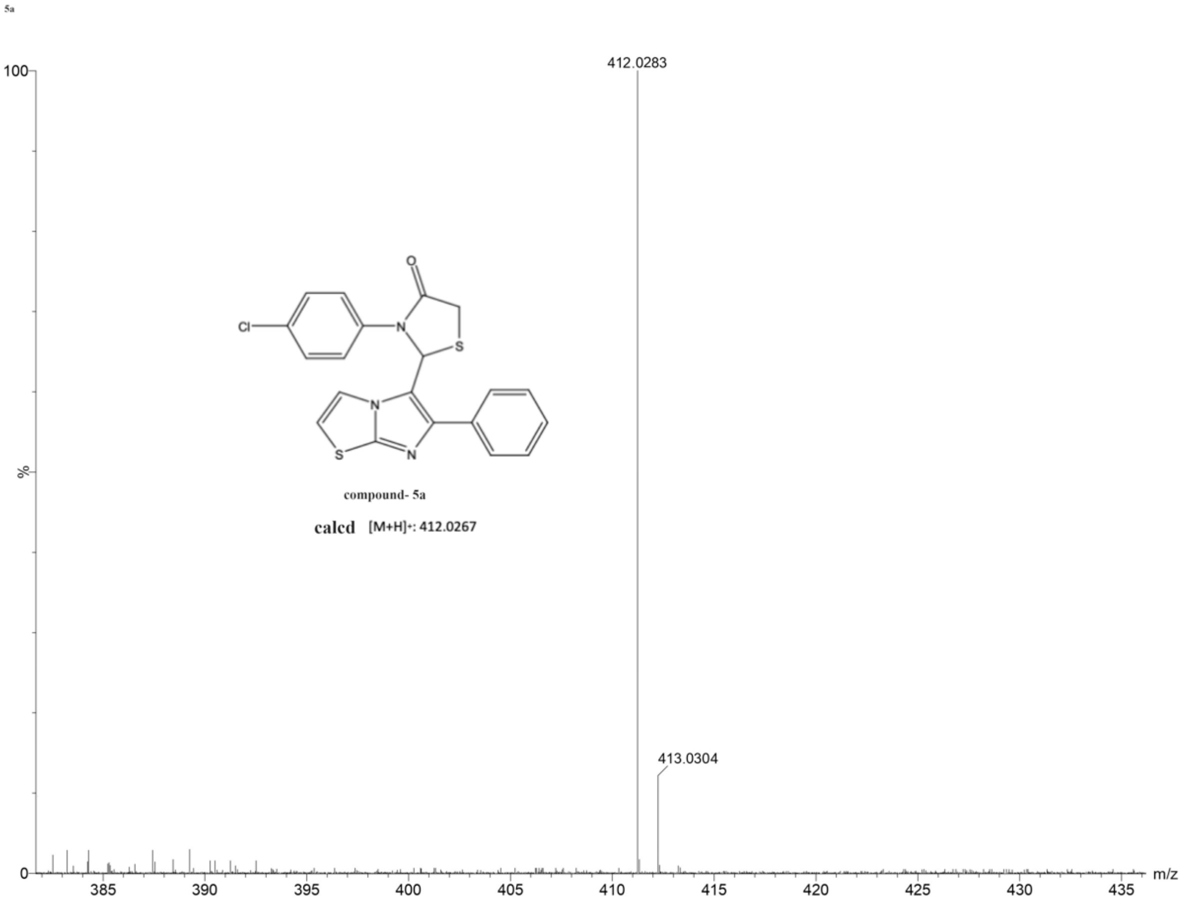

Figure S24. HRMS spectra of compound 5a

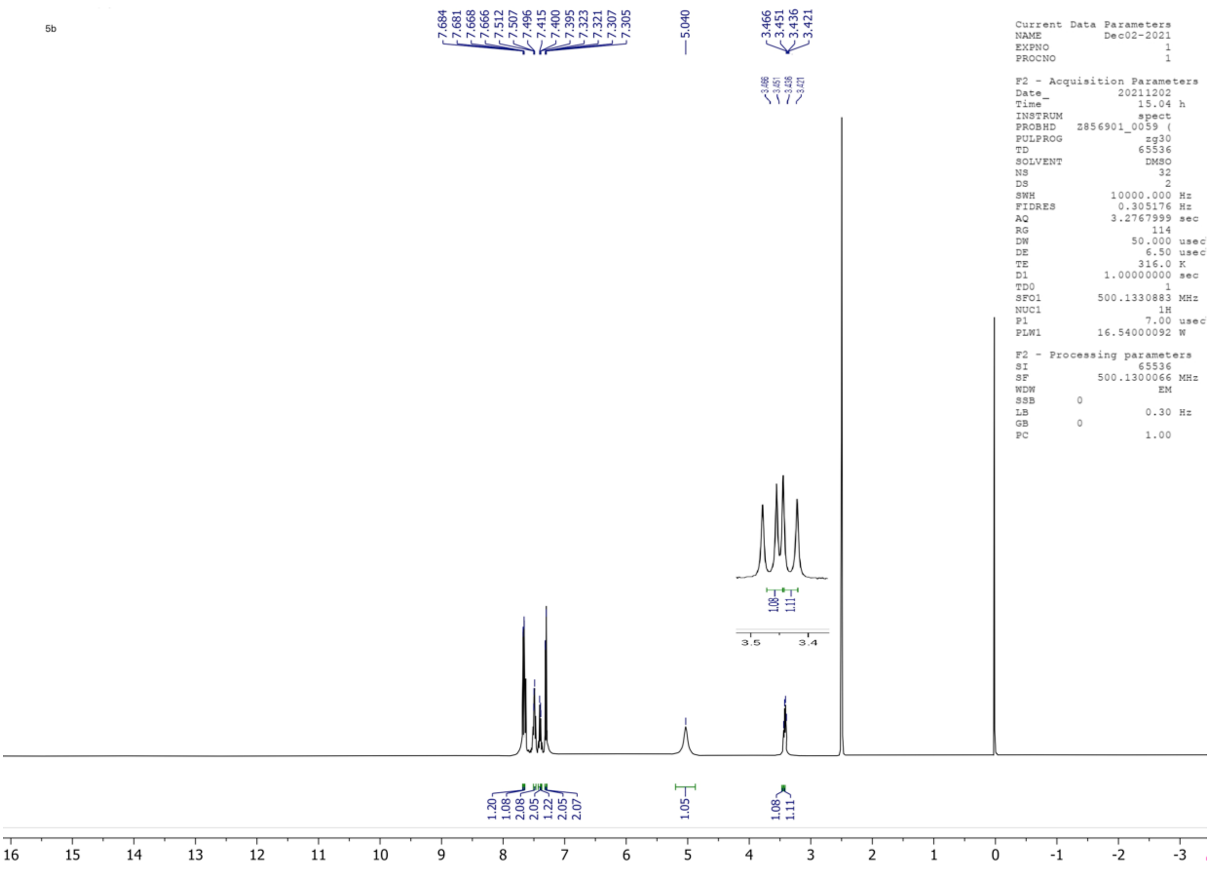

Figure S25. ^{1}H NMR spectra of compound **5b**

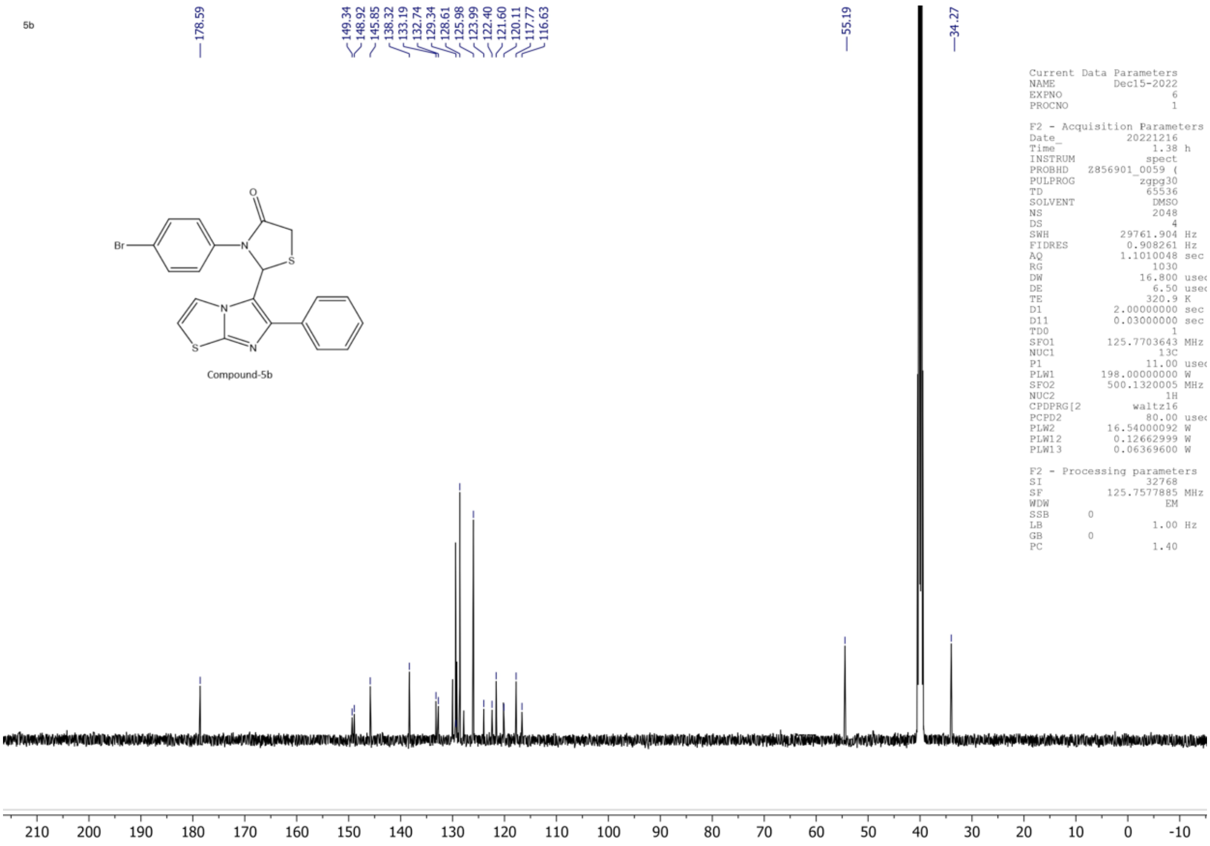

Figure S26. ^{13}C NMR spectra of compound **5b**

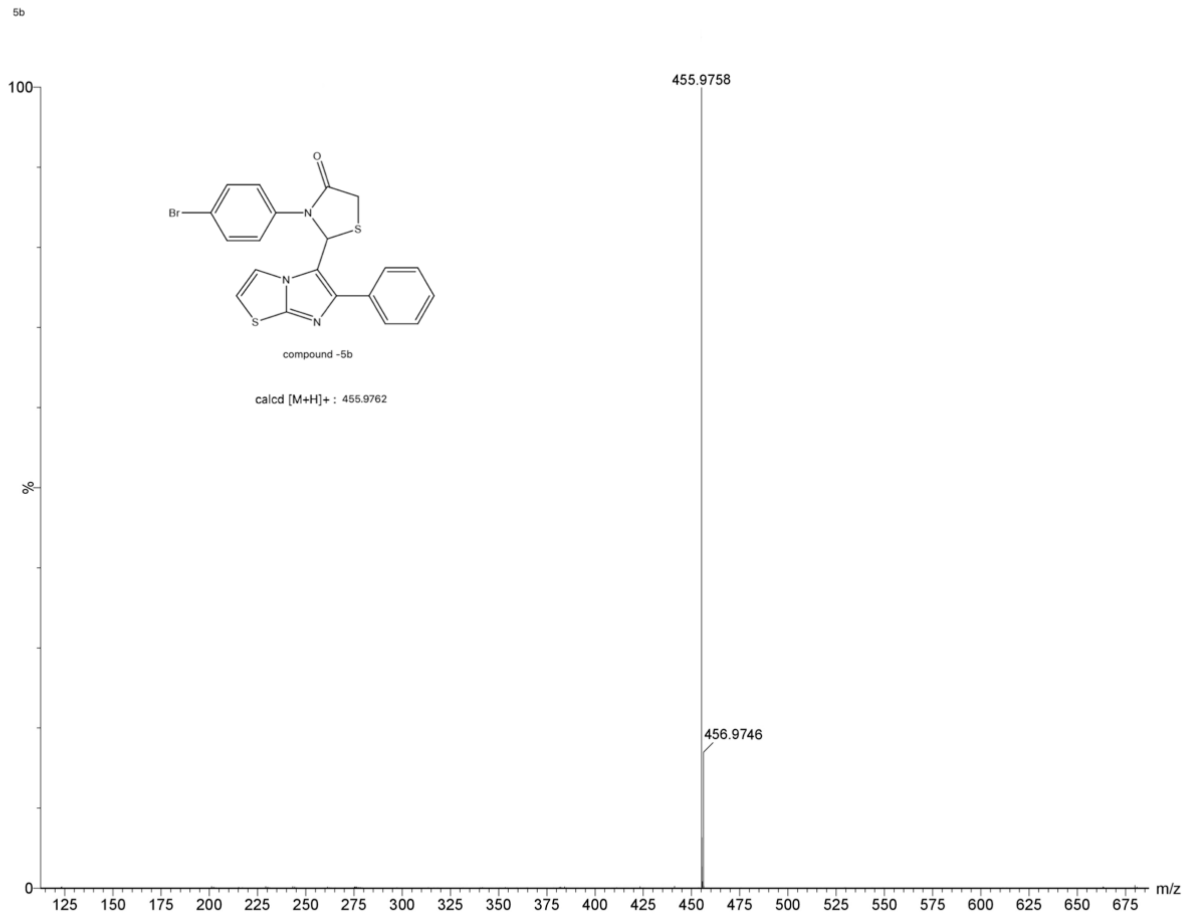

Figure S27. HRMS spectra of compound **5b**

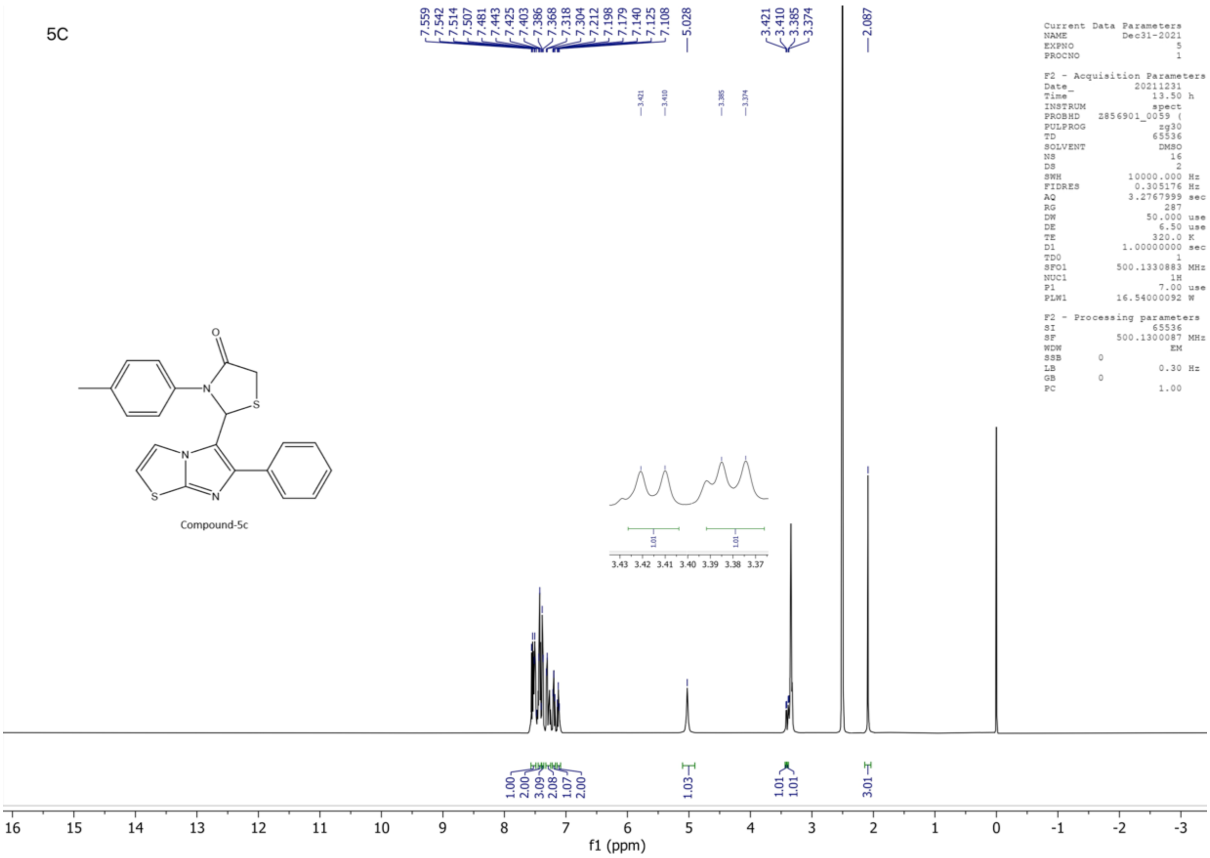

Figure S28. ^{1}H NMR spectra of compound **5c**

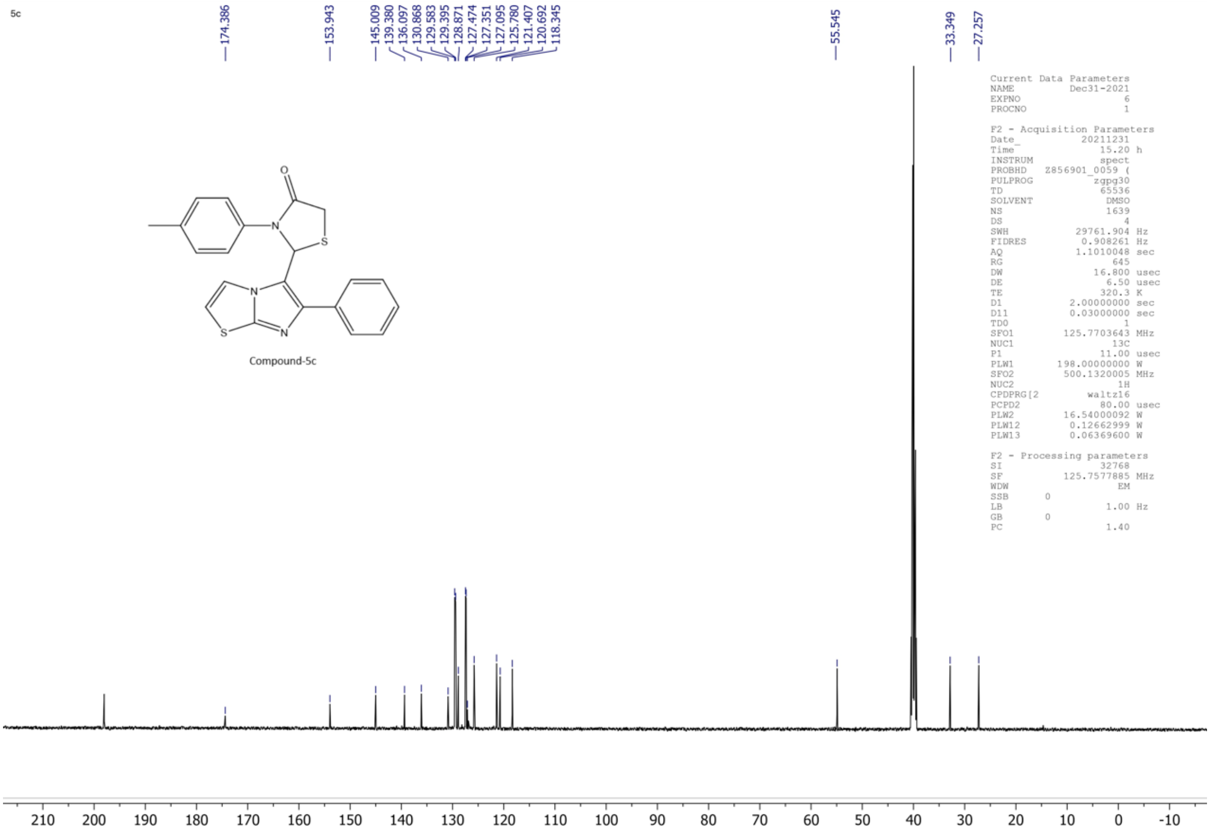

Figure S29. ^{13}C NMR spectra of compound **5c**

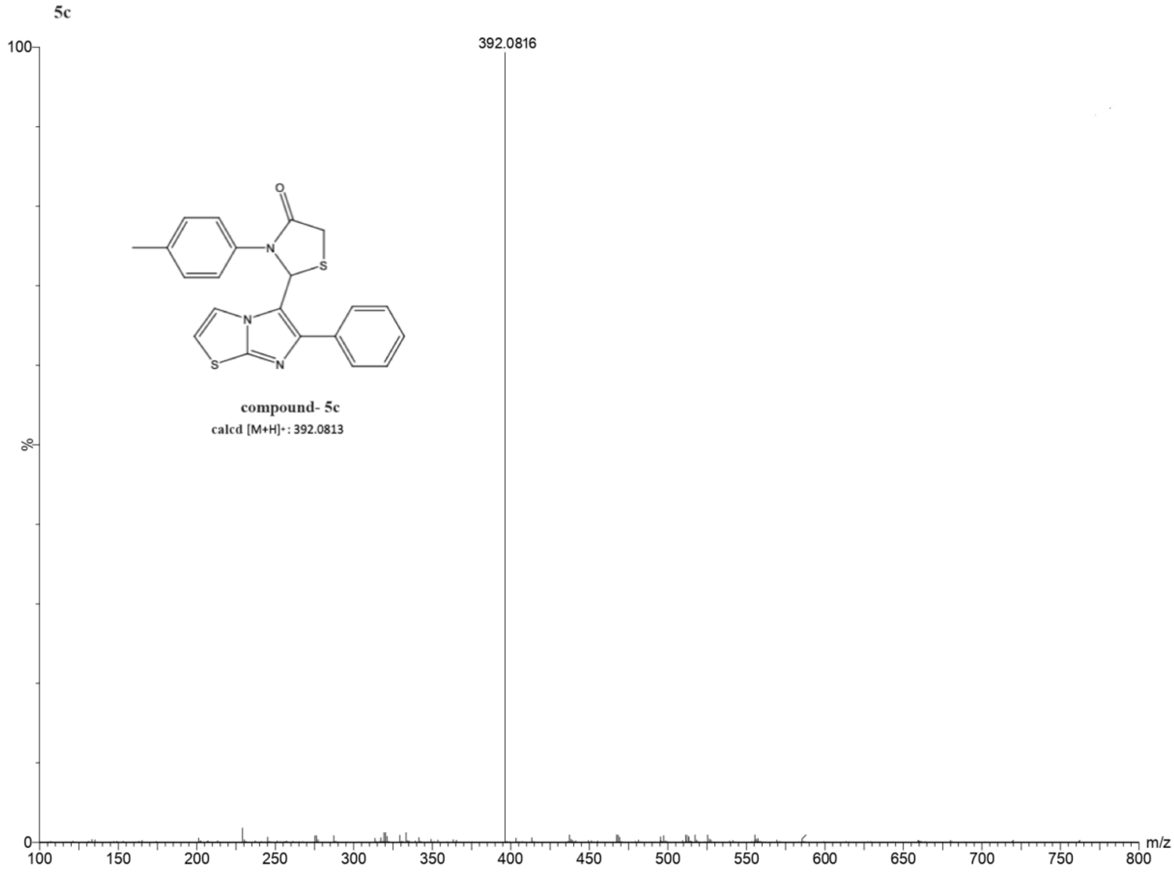

Figure S30. HRMS spectra of compound **5c**

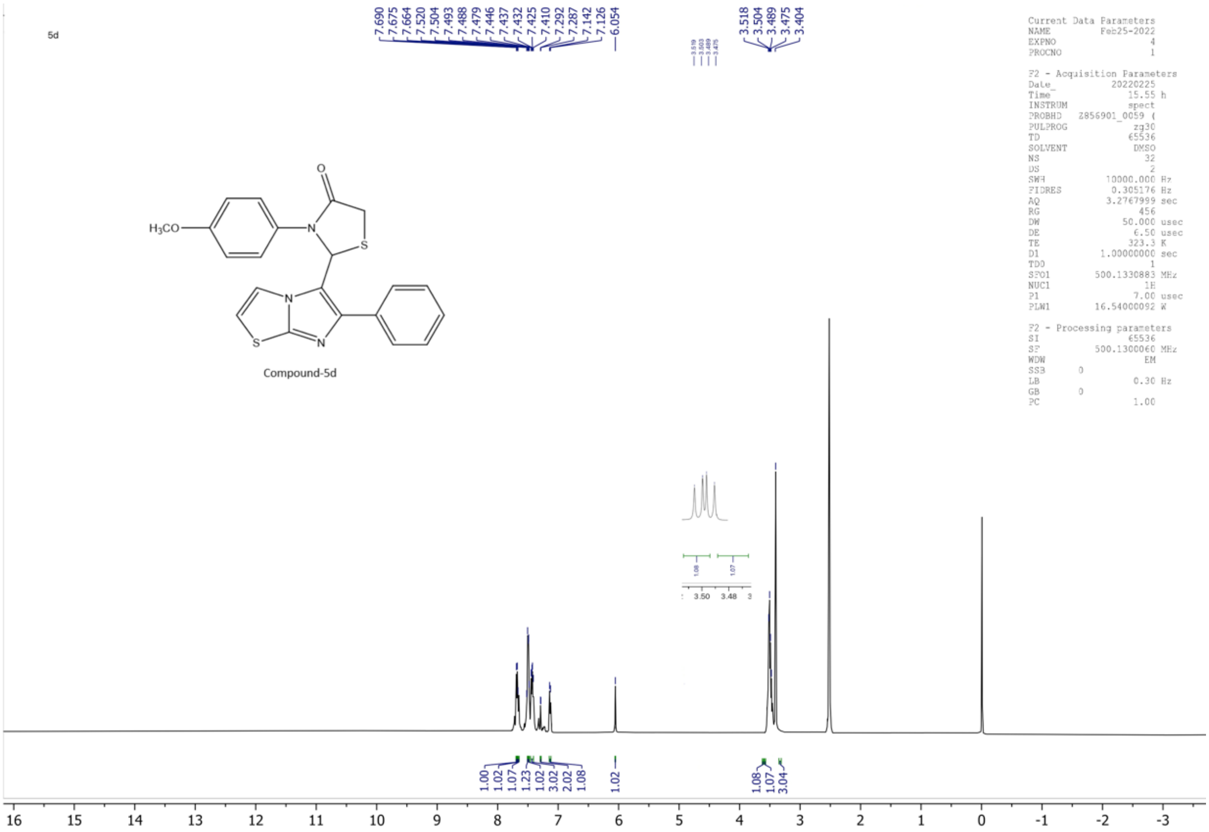

Figure S31. ^{1}H NMR spectra of compound **5d**

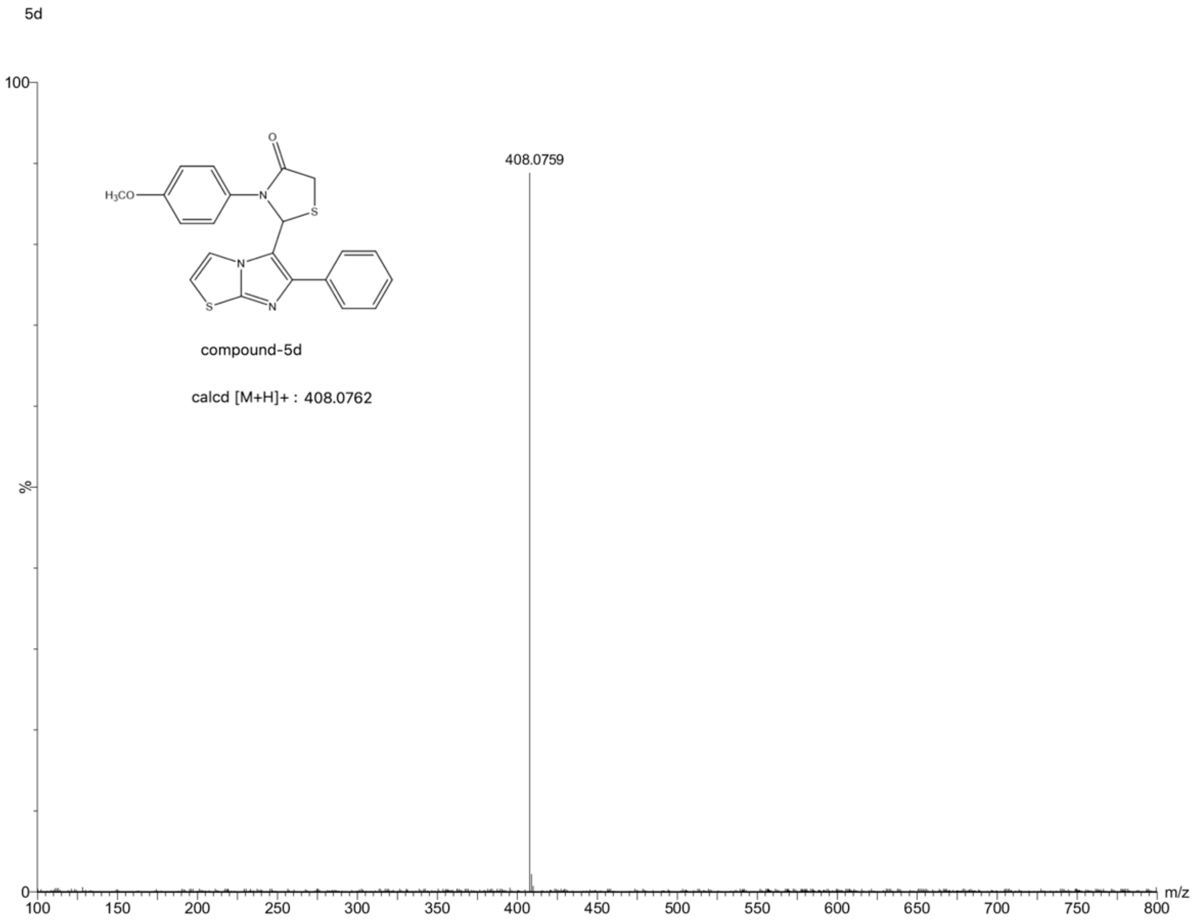

Figure S32. HRMS spectra of compound **5d**
